# Clinical practice guidelines for endometriosis in Japan (The 3rd edition)

**DOI:** 10.1111/jog.15416

**Published:** 2022-09-26

**Authors:** Tasuku Harada, Fuminori Taniguchi, Michio Kitajima, Jo Kitawaki, Kaori Koga, Mikio Momoeda, Taisuke Mori, Takashi Murakami, Hisashi Narahara, Yutaka Osuga, Ken Yamaguchi

**Affiliations:** ^1^ Department Obstetrics and Gynecology Tottori University; ^2^ Department Obstetrics and Gynecology Nagasaki University; ^3^ Department Obstetrics and Gynecology Kyoto Prefectural University of Medicine; ^4^ Department Obstetrics and Gynecology The University of Tokyo; ^5^ Department Obstetrics and Gynecology Aiiku Hospital; ^6^ Department Obstetrics and Gynecology Shiga University of Medical Science; ^7^ Department Obstetrics and Gynecology Oita University; ^8^ Department of Gynecology and Obstetrics Kyoto University Graduate School of Medicine

## Introduction

More than 10 years following the publication of “The General Rules for Clinical Management of Endometriosis: The second edition” in January 2010, the long‐awaited third edition has arrived. The second edition represents the bible for endometriosis treatment and has become the go‐to reference in clinical practice in Japan; it was published after the introduction of low‐dose estrogen–progestin (LEP) therapy and the novel progestin dienogest on the market. Looking back, this was the turning point from the era when laparoscopic surgery + GnRH agonist therapy was the predominant treatment and introduced an era of novel treatments. With the development of lightweight, high‐resolution endoscopes that allow for more delicate and precise surgical operations, endometriosis surgery has transitioned from laparotomy to laparoscopy. Conversely, despite their potency in alleviating the symptoms of endometriosis, GnRH agonists could only be used for up to 6 months due to adverse effects associated with estrogen deficiency. Thus, the predominant treatment was centered on laparoscopic surgery, with GnRH agonists used as an adjunct. Following the publication of the second edition, the use of LEP and dienogest became widespread. With both agents have been shown to be effective and safe, long‐term medical therapy has become possible. Owing to technical advancements in laparoscopic surgery, surgery is now also performed for deep endometriosis or less common and rare site endometriosis. However, for ovarian endometriotic cysts, ova, and normal ovarian tissues should be resected along with the endometrial tissue, thus, diminishing the ovarian reserve. Therefore, surgery must be performed carefully for young women and women wishing to have children in the future. We are now in an era in which we take advantage of long‐term medical therapy and laparoscopic surgery, as we consider treatment tailored to the life stages of individual women while attempting to preserve ovarian functions.

In this revised edition, we refer to the second edition for fundamental knowledge in clearly elaborating current perspectives on the review of the diagnosis and treatment, in addition to the advance over the last 10 years. Therapeutic strategies are explained with detailed flow charts. The clinical questions (CQs) in the latter half of this edition are intended as a useful guide for decision‐making in clinical practice. The authors are all physicians working tirelessly at the frontiers of endometriosis research in Japan, and this edition has undergone multiple generous revisions amid their busy schedules. Integration of the overall structure, revision of inconsistencies and duplication among CQs, and other onerous editing tasks were undertaken with tremendous dedication by the secretaries on the Japan Society of Endometriosis, Drs. Fuminori Taniguchi, Michio Kitajima, Kaori Koga, Taisuke Mori, and Ken Yamaguchi. Once again, I would like to offer them my deepest gratitude.

I hope that this revised edition would be helpful in the treatment of women with endometriosis not only in Japan but worldwide.

Tasuku Harada

**Table of Contents**

**Introduction**

**Chapter 1** Review

1. Diagnosis
2. Treatment

**Chapter 2** Treatment flowchart

1. Pain
2. Infertility

**Chapter 3** Treatment guidelines (Clinical questions)

CQ 1 How should adolescents with suspected endometriosis be treated?

CQ 2 How should ovarian endometriotic cysts be managed in view of their potential for malignant transformation?

CQ 3 Do endometriosis and adenomyosis increase the risk of obstetric complications?

CQ 4 How should adenomyosis‐associated pain be managed?

CQ 5 How should adenomyosis‐associated infertility be managed?

CQ 6 Is endometriosis a risk factor for cardiovascular events?

CQ 7 Does endometriosis affect quality of life?

CQ 8 What to be aware of when performing conservative surgery for ovarian endometriotic cyst?

CQ 9 Is surgery for ovarian endometriotic cysts effective in improving fertility?

CQ 10 Is surgery for deep rectovaginal endometriosis effective in improving fertility?

CQ 11 Is surgery effective in improving fertility in women with peritoneal lesions?

CQ 12 Is assisted reproductive technology (ART) effective for endometriosis‐associated infertility?

CQ 13 Are hormone therapies effective for endometriosis‐associated infertility?

CQ 14 Is surgery effective for endometriosis‐associated pain (excluding deep lesions)?

CQ 15 Is surgery effective for deep rectovaginal endometriosis‐associated pain?

CQ 16 Are oral contraceptive/low dose estrogen‐progestin (OCs/LEPs) effective for endometriosis‐associated pain?

CQ 17 Are GnRH agonists effective for endometriosis‐associated pain?

CQ 18 Are oral progestins effective for endometriosis‐associated pain?

CQ 19 Is the levonorgestrel intrauterine system (LNG‐IUS) effective for endometriosis‐associated pain?

CQ 20 Is there evidence of a superior efficacy between oral contraceptive/low dose estrogen‐progestin (OCs/LEPs), GnRH agonists, and progestins for endometriosis‐associated pain?

CQ 21 Is medical therapy effective for deep endometriosis‐associated pain?

CQ 22 Are complementary and alternative therapies effective for endometriosis‐associated pain?

CQ 23 Are pre‐operative or post‐operative medical therapies effective at surgery for endometriosis‐ associated pain?

CQ 24 Is medical therapy following conservative surgery effective in preventing the recurrence of ovarian endometriotic cysts?

## Chapter 1: Review

1 Diagnosis
  Overview of Diagnosis
1 **Clinical significance of endometriosis diagnosis**
  A pathologically benign disease, endometriosis is unlike other malignancies that are diagnosed by the detection of lesions. In the absence of symptoms, the diagnosis of microlesions has almost no clinical value, as they often disappear naturally and do not necessarily enlarge. In contrast, when symptoms are present, the lesions can be identified to establish a diagnosis that then becomes the basis for treatment. However, even in asymptomatic cases, lesions of a certain size can cause various symptoms and require therapeutic intervention. Diagnostic therapy is considered in endometriosis. When symptoms such as dysmenorrhea strongly indicate endometriosis, the patient's response to hormone therapy is considered. However, endometriosis can be difficult to distinguish from functional disorders, such as functional dysmenorrhea; this line of thinking is acceptable in certain circumstances. The relationship between lesions and symptoms in endometriosis patients remains largely unknown, while some aspects of the etiology of the lesions are vague. Consequently, the clinical significance of the diagnosis may change in the future.
2 **Methods for diagnosing endometriosis**
  Endometriosis is characterized by the presence and growth of endometrial tissue in locations other than the uterine cavity. While a definitive diagnosis should be made histologically, alternative diagnostic methods, such as laparoscopy are performed. The American Society of Reproductive Medicine (ASRM) staging system has become the most common and international system, and diagnosis based on laparoscopic findings has been recognized as the standard. However, laparoscopic findings have various limitations and flaws and therefore cannot necessarily serve as a perfect alternative to histological diagnoses. Ultrasonography and magnetic resonance imaging (MRI) have later been incorporated into the diagnosis of endometriosis. As diagnostic imaging has improved in quality, invasive laparoscopic diagnosis has somewhat diminished in relative value, and the exclusive use of laparoscopy for diagnosis has become much less common. Subjective and objective findings and biochemical tests are all useful only when combined with the results of diagnostic imaging and laparoscopy; none are used separately for diagnosis.
3 **Procedure for diagnosing endometriosis**
  The first steps in diagnosing endometriosis are an interview and a pelvic examination, as with a typical gynecological examination. Patients are asked in detail about the information related to endometriosis, such as family history, history of illness, menstrual history, and pregnancy/delivery history. If the history of the present illness includes pain, the chronological changes, properties, and sites of the pain are important. In vaginoscopy, observation of the posterior vaginal fornix is particularly important. If endometrial lesions are present, other methods can be used in addition to vaginoscopy to determine whether the lesions extend only to the vaginal wall or if they are continuous with deep endometriosis in the rectovaginal pouch. Subsequently, in the pelvic examination, important findings consist not only of the size, properties, and motility of the uterus and ovaries but also pain during examination and induration surrounding the rectouterine pouch. In a typical vaginal ultrasound during pelvic examination, asking the patient during observation whether the ultrasound probe cause any pain can be useful in the identification of lesions. If rectal endometriosis is suspected, a rectal examination may also be proactively performed to confirm the status of the bowel wall. However, when pelvic and rectal examinations are difficult, such as in the cases of young women and women who have not had sexual intercourse, a diagnosis should be established according to the results of other tests such as MRI, which yields even more detailed information regarding lesions. The finding of a serum CA 125 test can be used in combination with other tests as necessary, for differentiation with malignancy and to assess lesion activity.
**Details of diagnosis**
1 Subjective findings
  Commonly cited symptoms of endometriosis include menstrual pain (abdominal pain and low back pain) and infertility. In a survey of patients definitively diagnosed with endometriosis by the Japan Endometriosis Association, 88% of the patients experienced menstrual pain, and nonmenstrual abdominal pain, low back pain, dyspareunia, and dyschezia. Approximately half of the patients who responded reported infertility. Although uncommon, when lesions spread beyond the pelvic organs, symptoms may manifest elsewhere, such as in the gastrointestinal tract, urinary tract, or respiratory system (Table 1).
2 **Findings of the pelvic examination and rectal examination**
  Adhesions secondary to endometrial lesions often develop in the rectovaginal pouch and around the adnexa and are a characteristic finding of endometriosis in pelvic examinations. The findings indicative of endometriosis include limited uterine motility, uterine retroflexion, tenderness, and induration of the rectouterine pouch. Rectal examination is crucial in the diagnosis of rectouterine endometriosis. Rectovaginal pouch lesions that have formed small masses may become fibrotic and be palpable as indurations with tenderness. However, these lesions are evidently palpable on rectal examination.
3 **Biochemical tests**
  Although there are no definitive biomarkers to diagnose endometriosis, CA125 and CA19‐9 levels are often mildly elevated in patients with endometriosis.^1^ Although these biomarkers do not have high diagnostic accuracy individually, they are useful for diagnosis when combined with other findings. Sometimes, the levels of these biomarkers increase abruptly in the rupture and acute phase of hemorrhage of ovarian endometriotic cysts. CA125 and CA19‐9 levels seldom indicate positivity for early endometriosis and are useful only in advanced cases.^2^, ^3^ Serum HE4, in combination with serum CA125, is useful for differentiating between epithelial ovarian cancer and ovarian endometriotic cysts.^4^
4 **Diagnostic imaging**
  - Ultrasound findings in endometriosis
    Ultrasonography is emphasized as a means of diagnosing endometriosis and determining its course. Vaginal ultrasonography is generally superior to abdominal ultrasonography for visualizing pelvic lesions and is therefore preferred. However, for women who have not had sexual intercourse, either abdominal ultrasonography is performed or ultrasonography is performed rectally with a vaginal probe. If endometriosis is suspected, ultrasonography is performed in which the entire pelvis is observed first according to a set procedure and then the details. For example, briefly scan the uterus and ovaries to check for endometriosis and ovarian endometriotic cysts. Next, determine any adhesion associated with endometriosis but not with the endometrial lesions themselves. Check for site‐specific pain, ovarian mobility, and sliding between the uterus and surrounding organs. Lastly, evaluate the deep endometriosis in the region of the uterus, as necessary.
1 **Ultrasound findings in uteruses**

**TABLE 1:** Symptoms of endometriosis

・Pelvic pain
Menstrual pain (dysmenorrhea), dyspareunia, lower abdominal pain, low back pain
・Abnormal menstruation
Hypermenorrhea, irregular menstruation, abnormal vaginal bleeding
・Infertility
・Gastrointestinal symptoms
Abdominal pain, dyschezia, hematochezia, constipation, diarrhea
・Urinary symptoms
Pollakiuria, dysuria, hematuria
・Respiratory symptoms

In advanced endometriosis, adhesion between the rectum and the uterus causes rectovaginal pouch obliteration, which often results in retroversion and retroflexion of the uterus. In addition, deep endometriosis adjacent to the rectouterine pouch is occasionally continuous with the posterior uterine wall and adenomyosis, meaning that adenomyosis lesions can sometimes be used as clues to diagnose deep endometriosis.

2 **Ultrasound findings in ovarian endometriotic cysts**

In vaginal ultrasonography, resolution has improved not only for internal echoes but also for the margins and contours of cysts. Furthermore, wall thickness, wall irregularity, and adhesions to surrounding tissue can now be assessed more accurately. The accumulated blood in cysts is often old and sometimes develops irregular clots and hemosiderin deposits that require careful observation. Some ovarian endometriotic cysts are difficult to differentiate from mature ovarian teratomas and ovarian cancer and therefore cannot be reliably differentiated with ultrasonography alone.

**FIGURE 1:**
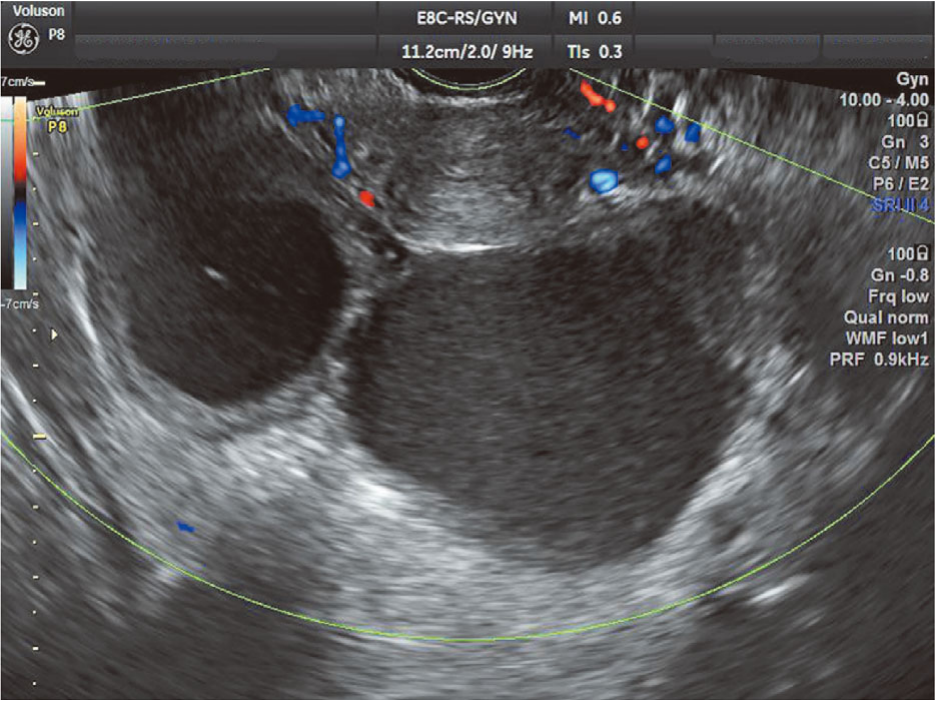
Transvaginal ultrasound coronal plane image. Bilateral ovarian endometrial cysts are localized in the rectovaginal pouch. Shown are cysts adherent to the ovarian tissue and to each other.

**FIGURE 2:**
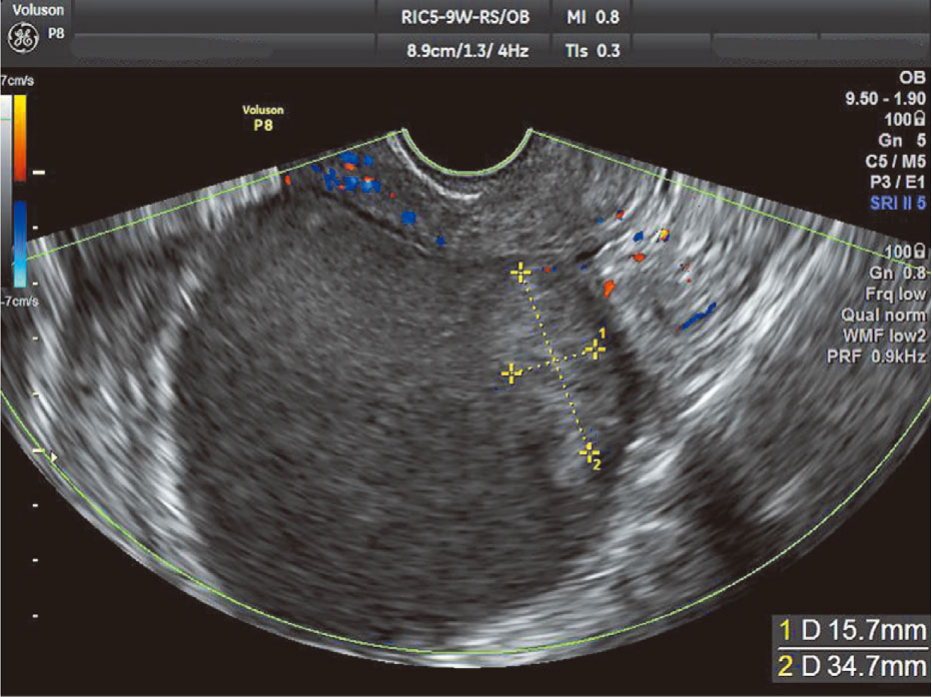
Transvaginal ultrasound sagittal plane image. Color Doppler enables confirmation that sites with solid echoes have no blood flow.

- Sites of cysts
  Ultrasound findings for ovarian endometriotic cysts present as nearly circular or elliptical unilocular or multilocular cysts that adhere to the uterus and are often located in the posterior aspect of the uterus or the rectovaginal pouch (Figure 1).
- Ultrasound findings of cysts
  1. Cyst wall
    The wall is seldom uniformly thin and often appears to be thickened. However, the images show blood clots and fibrin clots, not neoplastic thickening; papillary projections are also not observed in epithelial tumors.
  2. Internal echoes
    The inside of an ovarian endometriotic cyst is formed by accumulated blood and therefore presents with low, medium, or high intensity based on the density of free‐floating blood. Uniform−diffuse echoes or low‐intensity punctate−spotty echoes are often observed throughout the mass or in the inferior portion of the mass. When fibrin deposits are present, various hyperechoic regions and solid echoes are also observed. Fibrin clots demonstrate fluidity due to postural changes and present with a rippling motion when the cysts are vibrated from the abdominal wall manually or with a vaginal probe. Color Doppler can confirm the absence of blood flow in fibrin clots. The insides of the cysts occasionally present with relatively low‐intensity solid echoes. As cysts can be localized near the wall, it is important to differentiate ovarian mature teratomas with ovarian cancer (Figure 2). Hair shafts and sebum in ovarian mature teratomas and papillary projections in cancer are often hyperechoic. Although cysts are often unilocular, they sporadically have a few loculi, in which case septa are depicted. The surfaces of these septa are relatively smooth, and the septa do not present with dendritic or papillary projections. In the case of thick, irregular septa, ovarian cancer must be considered. In rare cases, mixed tumors comprising serous cysts or ovarian mature teratomas may develop in the ipsilateral ovary. When several various echo patterns are present, mixed tumors must be considered.

3 Ultrasound findings in deep endometriosis.^5^, ^6^
  Although deep endometriosis has conventionally been defined as endometriosis involving deep infiltration (5 mm) from the peritoneal surface, this view is now considered as outdated. Recently, lesions that arise with fibrous or myogenic tissue from adenomyosis‐like tissue (adenomyosis externa) have often been clinically addressed as deep endometriosis.
  Deep endometriosis most likely occurs in the vesicouterine pouch, the rectovaginal pouch, or the uterosacral ligaments. On ultrasonography, deep endometriosis is typically visualized as a hypoechoic area. To observe the vesicouterine pouch, a probe is inserted into the anterior vaginal fornix. To observe the bladder wall, storing urine in the bladder creates contrast, to visualize endometriosis; multiple small cystic echoes are often detected on the surface of the bladder. To observe the rectovaginal pouch or the uterosacral ligaments, a probe is inserted into the posterior vaginal fornix. Deep endometriosis generally involves rectovaginal pouch obliteration. The status of rectovaginal pouch obliteration can be determined based on the height of intraperitoneal effusion. In deep endometriosis of the bowel wall, the rectal serous membrane and mucosal surface are visualized as hyperechoic areas surrounding the hypoechoic deep endometriosis area, thus, confirming the diagnosis. While normal uterosacral ligaments are difficult to distinguish from the surrounding tissue in an ultrasound, deep endometriosis results in hypoechoic thickening, facilitating confirmation of the ligaments.

b MRI diagnosis

**FIGURE 3:**
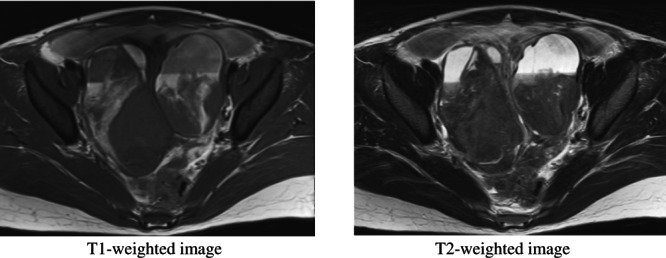
Pelvic MRI findings. In the early stage of a hemorrhage, ovarian endometriotic cysts contain different stages of clotting and therefore present with a variety of MRI signals.

**FIGURE 4:**
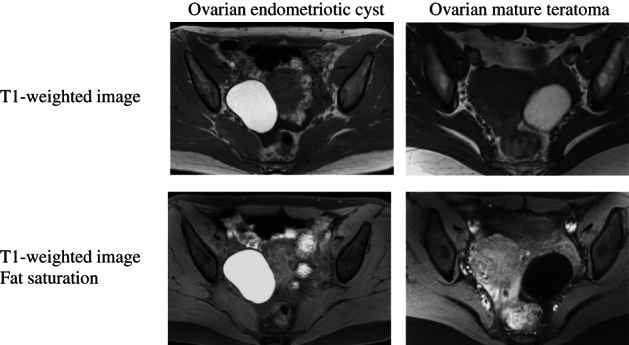
MRI findings. Ovarian mature teratomas are visualized with low signal intensity due to suppression of fat signals (lower right), whereas ovarian endometriotic cysts are not suppressed and continue to be visualized with high signal intensity (lower left).

**FIGURE 5:**
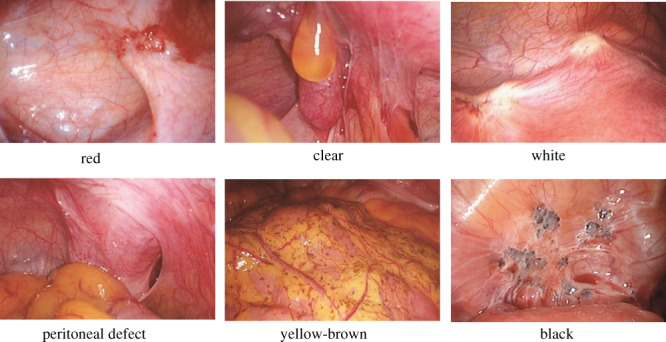
Representative photos corresponding to r‐ASRM classifications

1. The role of MRI diagnosis
  MRI can play a major role in diagnosing endometriosis because MRI “can specifically diagnose the blood flow based on signals.” Hematomas present with characteristic signals from bleeding that change over time, facilitating the deduction of timing and composition (Table 2, Figure 3).^7^ The values of sensitivity and specificity of MRI for differentiating between ovarian endometriotic cysts and adnexal masses are extremely high, 95% and 91%, respectively, making MRI a reliable cornerstone for deciding on a therapeutic strategy.^8^ In contrast, small implants in the peritoneal surface and membranous adhesion are difficult to diagnose. Thus, although MRI is inferior to laparoscopy in terms of the exclusion and staging of endometriosis, it is worthwhile as a non‐invasive method that can potentially reliably diagnose ovarian endometriotic cysts. Thus, the two types of tests are considered to complement each other. Due to the absence of other reliable methods for diagnosing deep/bladder/rectal endometriosis, MRI diagnosis contributes greatly to clinical practice.
2. Imaging methods
  T1‐weighted images and T2‐weighted images must be taken in the same plane. Fat suppression is useful for differentiating between fat and blood. In addition, contrast media are sometimes necessary to differentiate between clots in ovarian endometriotic cysts and solid tissue indicative of ovarian cancer. The images are taken in the transverse plane in principle, although sagittal T2‐weighted images are also often taken to observe adhesion between the uterus and ovaries as well as adhesion between the rectum and uterus. In addition, infusing water‐soluble gel into the vagina and rectum to create contrast improves the diagnosis of deep endometriosis adjacent to the rectovaginal pouch.^9^ Diffusion‐weighted imaging and apparent diffusion coefficient are useful for differentiating endometriosis from malignancy.^10^ Susceptibility‐weighted imaging is reported to be useful for detecting microbleeding in endometriosis.^11^
3. Imaging findings
  Malignant ovarian tumors must not be misdiagnosed as ovarian endometriotic cysts. Ovarian endometriotic cysts can be confirmed in one of two ways: presentation of high signal intensity similar to that of subcutaneous fat on T1‐weighted images and the presence of two or more cysts with signals not suppressed in fat‐suppressed imaging, or similar cysts presenting with heterogenous low signal intensity on T2‐weighted images (shading). The former demonstrates that the cysts are multiple cysts with blood, while the latter demonstrates that the cyst fluid is old blood that has become viscous, thereby, enabling differentiation with other hemorrhagic masses. In the images, only the pronounced adhesive changes, not the slight ones, can be identified, but not the slight adhesive changes. Adhesion must be suspected when the bilateral ovaries are in contact with each other, when the ovary is in contact with the uterus or pelvic wall over a wide area, or when the intestine appears to be pulled in a beak shape toward the uterine serosal surface or the ovary.
  Abdominal wall/deep/bladder/rectal endometriosis is depicted as fibrosis in MRI. Endometriosis is identified in the umbilical region, the groin, or scarring from gynecological operations, such as cesarean section or hysterectomy in T2‐weighted images as hypoechoic nodules and as hypertrophy limited to the bladder wall and bowel wall. However, in several cases, these findings are slight and easy to miss. The diagnosis is definitive if a 2–3‐mm section of tissue presenting with high signal intensity on T1‐ and T2‐weighted images is observed in the center of the endometriosis.
4. Differential diagnosis
  Blood and fat can present with markedly high signal intensity on T1‐weighted images. Therefore, although differentiating ovarian endometriotic cysts from ovarian mature teratomas is sometimes difficult, they are easily differentiated with fat saturation. Fat presents with low signal intensity due to signal suppression, whereas blood continues to present with high signal intensity (Figure 4). The presence of chemical shift artifacts and morphological characteristics also help with differentiation; however, fat suppression is the most reliable method.
  It is important not to make a diagnosis of ovarian endometriotic cysts based solely on a cyst presenting with high signal intensity in T1‐weighted images (i.e., signals indicating hematomas). This finding alone is not enough to differentiate an ovarian endometriotic cyst from a corpus luteum hemorrhage or ovarian tumoral hemorrhage. The corpus luteum is often 1–2 cm in size and disappears when observed during a menstrual cycle. Images of deposition of high‐signal intensity tissue only in the base of a cyst show the hematocrit effect and indicate functional intracystic hemorrhage, such as in the corpus luteum. With ovarian tumoral hemorrhage, due to the mixture of blood and other fluids in cysts, the fluid often does not present with a signal intensity as high as that of blood or fat.
  Carcinomas such as clear cell carcinoma and endometrioid carcinoma rarely develop from ovarian endometriotic cysts. Even when all findings suggest ovarian endometriotic cysts, ovarian carcinoma should be suspected when the mass has a solid portion inside it. Differentiation between small mural nodules and clots adhering to walls based on contrast‐enhanced MRI can also aid diagnosis. Clots have no blood flow and do not show contrast, whereas solid mural nodules show strong contrast. Although contrasted solid areas also appear in ovarian endometriotic cysts decidualization, such as that observed during pregnancy, diffusion‐weighted imaging is useful for differentiation.^10^
  Hydrosalpinx also presents with a variety of signals and can be difficult to differentiate from ovarian endometriotic cysts. However, a close examination of the hydrosalpinx often reveals it to have a tubular structure.
5. **Laparoscopic examinations**
  Laparoscopic examination is highly useful for endometriosis and is necessary for a definitive diagnosis. The r‐ASRM classification of endometriosis is widely used as a clinical staging system.^12^ This classification is used as an objective indicator to demonstrate the status of foci in laparoscopic visual examination and to determine severity according to a point‐summing scoring system. However, this classification system is not necessarily correlated with symptoms, such as pain or with pregnancy rates after treatment.^13^ The r‐ASRM classification system is superior to others in that, unlike other systems, it incorporates the recording of the colors of peritoneal lesions. Although the colors of peritoneal lesions have been reported to be associated with disease activity, red, black, and white lesions have also been indicated to potentially reflect an aging process.^14^
  Table 3 shows the r‐ASRM classification. Photos from actual cases corresponding to each classification of peritoneal lesion are also presented (Figure 5).

**TABLE 2:** Blood signals and changes by phase in MRI imaging

	T1‐weighted	T2‐weighted
Acute phase	Moderate	Low intensity
Subacute phase	High intensity	High intensity in margins, low intensity in center
Chronic phase	High intensity	High intensity

**TABLE 3:** R‐American Society of Reproductive Medicine (ASRM) classifications

		<1 cm	1–3 cm	>3 cm
Peritoneum	Superficial	1	2	4
	Deep	2	4	6
Ovary				
R	Superficial	1	2	4
	Deep	4	16	20
L	Superficial	1	2	4
	Deep	4	16	20
Adhesions		<1/3 Enclosure	1/3–2/3 Enclosure	>2/3 Enclosure
Ovary				
R	Filmy	1	2	4
	Dense	4	8	16
L	Filmy	1	2	4
	Dense	4	8	16
Tube				
R	Filmy	1	2	4
	Dense	4^a^	8^a^	16
L	Filmy	1	2	4
	Dense	4^a^	8^a^	16
Posterior culdesac obliteration	Partial	4		
	Complete	40		

*Note*: Stage I (Minimal): 1–5; Stage II (Mild): 6–15; Stage III (Moderate): 16–40; Stage IV (Severe): > 40. Denote appearance of superficial implant types as red [(R), red, red‐pink, flamelike, vesicular blebs, clear vesicles] (R), white [(W), opacifications, peritoneal defects, yellow‐brown], or black [(B). black, hemosiderin deposits, blue]. Denote percent of total described as R%, W%, and B%. Total should equal 100%.

^a^ If the fimbriated end of the fallopian tube is completely enclosed, change the point assignment to 16.

References1

Rokhgireh
S
, 
Kashi
AM
, 
Chaichian
S
, 
Delbandi
AA
, 
Allahqoli
L
, 
Ahmadi‐Pishkuhi
M
, et al. The diagnostic accuracy of combined enolase/Cr, CA125, and CA19‐9 in the detection of endometriosis. Biomed Res Int. 2020;2020:5208279–9.3306268110.1155/2020/5208279PMC75454352

Sasamoto
N
, 
DePari
M
, 
Vitonis
AF
, 
Laufer
MR
, 
Missmer
SA
, 
Shafrir
AL
, et al. Evaluation of CA125 in relation to pain symptoms among adolescents and young adult women with and without surgically‐confirmed endometriosis. PLoS One. 2020;15:e0238043.3283399810.1371/journal.pone.0238043PMC74448093

Kurdoglu
Z
, 
Gursoy
R
, 
Kurdoglu
M
, 
Erdem
M
, 
Erdem
O
, 
Erdem
A
. Comparison of the clinical value of CA 19‐9 versus CA 125 for the diagnosis of endometriosis. Fertil Steril. 2009;92:1761–3.1963131910.1016/j.fertnstert.2009.05.0224

Kadija
S
, 
Stefanovic
A
, 
Jeremic
K
, 
Radojevic
MM
, 
Nikolic
L
, 
Markovic
I
, et al. The utility of human epididymal protein 4, cancer antigen 125, and risk for malignancy algorithm in ovarian cancer and endometriosis. Int J Gynecol Cancer. 2012;22:238–44.2221496410.1097/IGC.0b013e318234f8525

Guerriero
S
, 
Condous
G
, 
van den Bosch
T
, 
Valentin
L
, 
Leone
FPG
, 
van Schoubroeck
D
, et al. Systematic approach to sonographic evaluation of the pelvis in women with suspected endometriosis, including terms, definitions and measurements: a consensus opinion from the international deep endometriosis analysis (IDEA) group. Ultrasound Obstet Gynecol. 2016;48:318–32.2734969910.1002/uog.159556

Collins
BG
, 
Ankola
A
, 
Gola
S
, 
McGillen
KL
. Transvaginal US of endometriosis: looking beyond the endometrioma with a dedicated protocol. Radiographics. 2019;39:1549–68.3149874610.1148/rg.20191900457

Taguchi
A
, 
Koga
K
, 
Osuga
Y
, 
Fujimoto
A
, 
Miyasaka
A
, 
Yano
T
, et al. Successful management of a ruptured endometrial cyst in acute leukemia. Fertil Steril. 2011;95:292.e1–3.10.1016/j.fertnstert.2010.04.082205616148

Foti
PV
, 
Farina
R
, 
Palmucci
S
, 
Vizzini
IAA
, 
Libertini
N
, 
Coronella
M
, et al. Endometriosis: clinical features, MR imaging findings and pathologic correlation. Insights Imaging. 2018;9:149–72.2945085310.1007/s13244-017-0591-0PMC58934879

Tong
A
, 
VanBuren
WM
, 
Chamié
L
, 
Feldman
M
, 
Hindman
N
, 
Huang
C
, et al. Recommendations for MRI technique in the evaluation of pelvic endometriosis: consensus statement from the Society of Abdominal Radiology endometriosis disease‐focused panel. Abdom Radiol (NY). 2020;45:1569–86.3219359210.1007/s00261-020-02483-w10

Takeuchi
M
, 
Matsuzaki
K
, 
Harada
M
. Computed diffusion‐weighted imaging for differentiating decidualized endometrioma from ovarian cancer. Eur J Radiol. 2016;85:1016–9.2713006510.1016/j.ejrad.2016.03.00911

Cimsit
C
, 
Yoldemir
T
, 
Guclu
M
, 
Akpinar
IN
. Susceptibility‐weighted magnetic resonance imaging for the evaluation of deep infiltrating endometriosis: preliminary results. Acta Radiol. 2016;57:878–85.2631583810.1177/028418511560214712
American Society for Reproductive Medicine
. Revised American Society for Reproductive Medicine classification of endometriosis: 1996. Fertil Steril. 1997;67:817–21.913088410.1016/s0015-0282(97)81391-x13

Andres
MP
, 
Borrelli
GM
, 
Abrão
MS
. Endometriosis classification according to pain symptoms: can the ASRM classification be improved?
Best Pract Res Clin Obstet Gynaecol. 2018;51:111–8.3002995910.1016/j.bpobgyn.2018.06.00314

Strehl
JD
, 
Hackl
J
, 
Wachter
DL
, 
Klingsiek
P
, 
Burghaus
S
, 
Renner
SP
, et al. Correlation of histological and macroscopic findings in peritoneal endometriosis. Int J Clin Exp Pathol. 2013;7:152–62.24427335PMC3885469

**2. Treatment**

Overview of treatment

The first step in the treatment of endometriosis is to design a regimen that targets the most important and urgent symptom in the patient based on an accurate diagnosis of clinical endometriosis. In most cases, the treatment factors are often broadly divided into infertility, pain, and ovarian endometriotic cysts, and patients sometimes have two or all three of the above. In light of these factors, based on a thorough discussion with the patient, the most suitable of the following therapeutic strategies is selected: medical therapy, surgical therapy, fertility treatment, or follow‐up without treatment.

These symptoms markedly decrease the quality of life (QOL) for endometriosis patients at all stages of life from the onset of symptoms in adolescence or later until menopause. In adolescence, dysmenorrhea is a risk factor for endometriosis later in life^1^; therefore, possible interventions are performed as necessary, and mild endometriosis should be monitored to avoid becoming severe.

Once sexual maturity is reached, ovarian endometriotic cysts should be sufficiently removed in terms of the possibility of recurrence after treatment and the risk of malignant transformation. However, conservative therapy intended to preserve the ovarian reserve to maintain fertility conflicts with the removal of ovarian endometriotic cysts. Therefore, a therapeutic strategy must be decided with sufficient consideration of these factors. Furthermore, as recent studies have demonstrated, endometriosis itself is a risk factor for obstetric complications, such as preterm birth and hemorrhage.^2^, ^3^ These risks must also be considered.

Other recent studies have determined endometriosis to be a risk factor for heart disease.^4^, ^5^ Endometriosis must no longer be considered as a disease limited to the pelvis but rather as a systemic chronic inflammatory disease that must be managed until middle and old age. Instead of only focusing on symptoms during a specific period, a three‐dimensional therapeutic strategy that also considers age should be considered for endometriosis patients.

Details of treatment

1. Medical therapy
  Medical therapy for endometriosis is broadly divided into symptomatic treatment and hormone therapy. Symptomatic treatment consists mainly of analgesics and Chinese herbal medicine used to relieve pain associated with endometriosis. Currently, the only treatment that works on endometriotic foci is hormone preparations.
  Hormone therapy in Japan began in 1965, when the progestin dydrogesterone was introduced as treatment in the market, and pseudopregnancy therapy with medium‐dose oral contraceptives (OCs) was performed. In 1983, danazol was released on the market as a therapeutic drug for endometriosis and hormone therapy began to develop. In 1988, buserelin, the first GnRH agonist, was commercially released, followed by a succession of other GnRH agonists, which are still used today as potent therapeutics. In 1999, long‐awaited low‐dose OCs were introduced to treat endometriosis‐associated pain due to their side benefit. After approximately 9 years without the introduction of novel drugs, the fourth‐generation progestin dienogest was released in January 2008. The launch of the ethinylestradiol/norethisterone combination, the first LEP formulation, was in July 2008. Although these are the same components as in OC, OCs are used for birth control and are not covered by insurance, while LEPs have been covered by insurance for the treatment of dysmenorrhea since they were released. Other LEPs have since been released on the market. Currently, LEPs and dienogest are the most widely used therapies for endometriosis. Furthermore, the levonorgestrel‐releasing intrauterine system (LNG‐IUS), which was initially not covered by medical insurance as an intrauterine contraceptive device, is now covered by it for the treatment of dysmenorrhea and hypermenorrhea and is consequently now also used for patients with endometriosis. Recently, oral GnRH antagonist is covered by medical insurance for treating pelvic pain associated with endometriosis (Table 4).

**TABLE 4:** Medical therapy for endometriosis

I. Symptomatic treatment
(1) Non‐steroidal anti‐inflammatory drugs (NSAIDs)
(2) Chinese herbal medicine
Shakuyakukanzoto, tokishakuyakusan, keishibukuryogan, tokakujokito, and others
II. Hormone therapy
(1) Estrogen/progestin (pseudopregnancy therapy)
(2) Low‐dose estrogen/progestin combination (OC/LEP)
(3) Progestins
a. Dienogest
b. Dydrogesterone
(4) Danazol
(5) GnRH agonists
(6) GnRH antagonists
(7) Levonorgestrel‐releasing intrauterine system (LNG‐IUS)
(8) Aromatase inhibitors

The basic views of medical therapy include the following:

1. Medical therapy is used to eliminate menstrual pain, dyspareunia, and other forms of endometriosis‐associated pain.
2. In patients who are infertile, medical therapy may be administered with the expectation of establishing pregnancy by post‐medical therapy improvement of foci and improvement of the peritoneal environment.
3. The purpose of medical therapy is to enhance the efficacy of surgery by eliminating or removing endometriotic foci, and therapeutics may be administered postoperatively.
4. Medical therapy is used to delay the progression of foci to prevent the recurrence of endometriosis.

Although a definitive diagnosis of endometriosis requires both laparoscopic surgery and laparotomy, performing both in all cases is difficult. In daily clinical practice, clinical endometriosis is diagnosed according to clinical symptoms and various other methods, such as pelvic/rectal examination, diagnostic imaging (ultrasonography and MRI), and biochemical testing (CA 125 levels), with medical therapy selected after a diagnosis is established.

The ESHRE guideline for the diagnosis and treatment of endometriosis^6^ recommends non‐steroidal anti‐inflammatory drugs (NSAIDs), oral contraceptive/low dose estrogen‐progestin (OC/LEP), progestin, danazol, and GnRH agonists. The choice of agents is difficult to standardize as it obviously varies according to different factors, such as the patient's age, symptoms, the severity of foci (disease stage), the patient's desire to bear children, and previous treatment. Although all of these agents reduce or eliminate the menstrual pain associated with endometriosis, they can differ in their effects on other symptoms and foci. Therefore, in the selection of agents, adverse effects and the economic burden imposed on patients should be considered. In addition, medical therapy alone is unlikely to completely cure endometriosis. A suitable therapeutic regimen involves the combination of medical therapy and surgical therapy, along with assisted reproductive technology (ART) for patients who are infertile.

- Symptomatic treatment
  1. Non‐steroidal anti‐inflammatory drugs
    The primary mechanism of action of NSAIDs is inhibition of prostaglandin synthesis. They are the first‐line treatment for severe menstrual pain in unmarried women from adolescence, when women first complain of pain, to their twenties.
  2. Chinese herbal medicine
    In Chinese herbal medicine treatment, dysmenorrhea is interpreted as “congestion” or “water poisoning” and is widely treated with formulations such as shakuyakukanzoto (peony and licorice decoction), tokishakuyakusan (angelica and peony powder), keishibukuryogan (cassia twig and tuckahoe pill), and tokakujokito (peach kernel purgative decoction). The reasons for selecting Chinese herbal medicine include relief of menstrual pain in unmarried and adolescent adult women and the reduction of adverse effects associated with other drugs.
- Hormone therapy
  GnRH agonists have become widely used as a first‐line option in hormone therapy since they first emerged. However, since the launch of dienogest and OCs/LEPs, GnRH agonists have ceded their first‐line status and are chosen only when OCs/LEPs are not sufficiently effective.^7^ LNG‐IUS, which specifically releases hormones into the uterine cavity, is sometimes also used. Danazol is known to be effective; however, due to the risks of adverse effects, namely virilization and thrombosis, its use has declined in the recent years. When medical therapy fails to sufficiently inhibit pain, surgical therapy is considered.
  Ovarian endometriotic cysts recur frequently after conservative surgery, and postoperative hormone therapy is considered a means of preventing decreased ovarian reserve resulting from repeated surgeries.^8^
  OCs/LEPs^9^ and dienogest are effective for preventing the postoperative recurrence of ovarian endometriotic cysts. Six months of GnRH agonist administration followed by continuous administration of OC/LEP or dienogest is effective in preventing postoperative recurrence.^10^

1. Oral contraceptive/low‐dose estrogen/progestin (OC/LEP)
  OC/LEP, which are administered to suppress ovulation, contain estrogen and progestin. The long‐term use of OC/LEP combinations inhibits follicle maturation, ovulation, and luteinization. Specifically, inhibition of periodic changes in endogenous estrogens and progesterone, in turn, inhibits endometrial growth. OC/LEP also inhibit endometrial growth in the ectopic endometrium (endometriotic foci), which has characteristics similar to those of the orthotopic endometrium. Inhibition of endometrial growth reduces prostaglandin production, thus improving menstrual pain.
  A study conducted in 2008 in Japan reported on a placebo‐controlled randomized controlled trial (RCT) of ethinylestradiol 0.035 mg plus norethisterone 1 mg for endometriosis,^11^ which is now commercially available and covered by insurance.
  The main adverse effects reported associated with OC/LEP include abnormal uterine bleeding, nausea, headache, oligomenorrhea, upper abdominal pain, breast pain, and breast discomfort. These reactions peak in the first period after administration but then gradually decline; therefore, the adverse effects diminish with long‐term use. Furthermore, deep vein thrombosis can occur as a serious adverse effect in rare cases and therefore requires caution.
2. Progestins

- Dienogest
  Dienogest (17‐hydroxy‐3‐oxo‐19‐nor‐17α‐pregna‐4,9‐diene‐21‐nitrile; 17α‐cyanomethyl‐17β‐hydroxyestra‐4,9[10]‐dien‐3‐one), a derivative of 19‐nortestosterone developed in Germany, is a fourth‐generation progestin. Characteristically, although it has a potent endometrial differentiation effect, its effects of inhibition of gonadotropin secretion and anti‐estrogen effect are weak, it has no androgen effect, and it has a relatively potent anti‐androgen effect. Its observed mechanisms of action against endometriosis are as follows: (1) inhibition of ovulation, (2) inhibition of estrogen production by inhibiting follicle development, (3) endometrial differentiation and inhibition of endometrial cell growth, and (4) direct inhibition of endometriotic cell growth and inhibition of cytokine production (in vitro).
  Following a Japanese RCT that compared dienogest with the GnRH agonist intranasal buserelin,^12^ Japan became the first country worldwide to provide insurance coverage for dienogest, in January 2008.
- Dydrogesterone
  Dydrogesterone is a stereoisomer of progesterone with activity that resembles natural progesterone. It is characterized by its lack of estrogen and androgen effects and by its status as the only progestin formulation that does not increase basal body temperature. Its long history of use in Japan dates to its release on the market in 1965. Its indications include dysmenorrhea, infertility associated with luteal phase defect, threatened preterm labor, and endometriosis. At present, it is frequently used in luteal support in fertility treatment and in postmenopausal hormone replacement therapy.
  Following the emergence of danazol and GnRH agonists, which have potent therapeutic effects, the use of dydrogesterone for endometriosis has declined. Although few clinical studies have examined dydrogesterone since then, a 2007 study reported that dydrogesterone relieved pain in patients with endometriosis following laparoscopic surgery.^13^ In addition, a recent Japanese study reported that dydrogesterone had a therapeutic effect for women complaining of dysmenorrhea.^14^ Most other hormone preparations have potent therapeutic effects but inhibit ovulation and therefore cannot be used for the treatment of endometriosis in women who want to have children. Conversely, dydrogesterone has only a weak ovulation inhibition effect and has a luteal support effect, meaning that it can be used without affecting the ovulation cycle in patients. Considering these properties, dydrogesterone is being increasingly reexamined as an agent with few adverse effects and mild action.
- Levonorgestrel‐releasing intrauterine system (LNG‐IUS)
  LNG‐IUS, a progestin developed in Finland, is a T‐shaped intrauterine device (IUD) that combines a progestin with a more reliable contraceptive effect. The system continuously releases LNG 20 μg/day over a 5‐year period. The insertion of LNG‐IUS into the uterine cavity not only produces a high contraceptive effect by marked atrophying and thinning of the endometrium, but has also been shown to improve uterine fibroids, adenomyosis‐induced hypermenorrhea, and associated dysmenorrhea by markedly reducing menstrual bleeding.^15^ The ESHRE guideline^6^ recommends the use of LNG‐IUS to inhibit endometriosis‐associated pain. While LNG‐IUS prevents the recurrence of dysmenorrhea as OCs/LEPs, it does not prevent nonmenstrual pain or dyspareunia. Unlike other forms of hormone therapy, despite an increase in local drug concentration, systemic blood concentration remains low, giving LNG‐IUS the advantage of exerting few of the systemic adverse effects observed.

3 GnRH agonists.
  GnRH agonists, currently the most widely used agents in medical therapy for endometriosis in Japan, are 10 amino acid polypeptides that are not absorbed orally, but are instead administered nasally or subcutaneously.
  The continuous administration of GnRH agonists stimulates GnRH receptors in the pituitary gland, eventually reducing the number of receptors in the pituitary membranes (downregulation) and reducing the pituitary GnRH sensitivity, triggering the desensitization of gonadotropin‐producing cells. These mechanisms of action have been found to inhibit sex steroid secretion, leading to the use of GnRH agonists to treat endometriosis.
  The notable adverse effects consist of symptoms of ovarian hormone deprivation and bone loss associated with low estrogen states.
4 GnRH antagonists
  GnRH antagonists act directly on GnRH receptors to inhibit GnRH gonadotropin release. GnRH antagonists do not involve the temporary stimulation of gonadotropin secretion that occurs early in the administration of GnRH agonists (flare‐up) and inhibit gonadotropin production in a powerful and rapid manner. For these reasons, GnRH antagonists had been considered theoretically superior to GnRH agonists. However, histamine release, edema, anaphylaxis, and other problems have been reported in the early stage of the administration of the GnRH antagonist.
  The peptide GnRH antagonists, cetrorelix and ganirelix, and the nonpeptide GnRH antagonist, relugolix, are currently commercially available in Japan. However, peptide GnRH antagonists must be injected daily and are therefore difficult to use long‐term in patients with chronic conditions, such as endometriosis. Based on their indication of preventing premature ovulation under controlled ovarian stimulation, GnRH antagonists are widely used in controlled ovarian stimulation in ART but are not covered by insurance for that purpose.
  In response to this situation, Japan became the first country worldwide to provide insurance coverage for the oral medication, relugolix, for the indication of improving uterine fibroid‐based symptoms in March 2019. Phase 2 and 3 studies of relugolix intake in patients with endometriosis have been concluded.^16^, ^17^ Relugolix is now covered by insurance for treating endometriosis‐associated pelvic pain.
2 Surgical therapy

Among patients with endometriosis, surgical therapy is primarily intended for patients with pelvic pain and infertility. However, for ovarian endometriotic cysts, when patients present with the thickening of the cyst wall, mural nodules, or rapid cyst growth, surgical therapy is sometimes indicated even when there are few symptoms to allow differentiation from malignancy. In deep endometriosis, surgery is indicated for patients, such as those for whom medical therapy does not sufficiently improve symptoms, patients with severe pain who want to have children, and patients who are infertile. Surgical therapy for endometriosis involves laparotomy or laparoscopic surgery. For ovarian endometriotic cysts, laparoscopic surgery produces surgical effects equal to those of laparotomy but is superior in terms of reducing surgical invasiveness and is recommended as an option equivalent to laparotomy.^18^

Surgical therapy for endometriosis is broadly divided into conservative and radical surgery.

- Conservative surgery

Conservative surgery is selected for patients who wish to preserve their future fertility. The primary indications for conservative surgery are ovarian endometriotic cysts, peritoneal lesions, and deep lesions. Procedures for managing ovarian cysts consist of cyst removal or drainage/cyst wall ablation. Although both are reported to be effective in improving postoperative pain, cyst removal is associated with significantly lower rates of dyspareunia, pelvic pain, cyst recurrence, and additional surgery.^19^ However, it has been indicated that ovarian reserve declines after ovarian endometriotic cyst removal,^20^ which is a finding which warrants caution. The ablation and removal of deep lesions helps improve pain.^21^, ^22^ In patients with deep endometriotic lesions with painful induration primarily in the rectovaginal pouch, the removal of the lesions is effective for pain relief. In addition, the removal of the foci in infertile patients with deep endometriotic lesions has been indicated to potentially help increase fertility. However, the removal of foci in deep endometriotic lesions is difficult due to factors such as the risk of injury to the intestines, urinary system, and organs. Therefore, general healthcare facilities should consider referral to a high‐level medical facility to perform the procedure. The removal of foci also requires coordination with other departments, such as gastrointestinal and urologic surgery.

b Radical surgery

Radical surgery is typically chosen when the patient does not wish to have children, severe dysmenorrhea and pelvic pain are strongly associated with endometriotic foci, and medical therapy is not sufficiently effective. Hysterectomy is simultaneously performed when it can be expected this would eliminate these symptoms or when benign uterine diseases, such as uterine fibroids or adenomyosis, are also present. The extent of resection in radical surgery is considered on a case‐by‐case basis involving symptoms and the characteristics of the patient.

c Postoperative management and issues

1. Management after conservative surgery
  Ovarian endometriotic cysts are known to recur frequently after removal. Therefore, patients must be encouraged to make regular postoperative outpatient visits to check for recurrence. The long‐term preventive administration of OC/LEP combinations is reported to inhibit pain and recurrence.^9^ Therefore, their indication should be considered on a case‐by‐case basis. In addition, dienogest for postoperative recurrence has been reported to reduce the need for repeat surgeries.^23^
2. Management after radical surgery
  When deep lesions persist despite the removal of foci with hysterectomy, the failure to sufficiently resect the foci may prevent sufficient alleviation of pelvic pain.^24^ In such cases, dienogest or other forms of medical therapy are considered.
  When the ovaries are removed as radical surgery, estrogen replacement therapy (ERT) is often initiated shortly after oophorectomy to alleviate the symptoms of ovarian deficiency and prevent bone loss associated with low estrogen. However, it is important to keep in mind the possibilities of recurrence of residual endometriotic foci and the occurrence of secondary tumors.

References1

Nnoaham
KE
, 
Webster
P
, 
Kumbang
J
, 
Kennedy
SH
, 
Zondervan
KT
. Is early age at menarche a risk factor for endometriosis? A systematic review and meta‐analysis of case‐control studies. Fertil Steril. 2012;98:702–712.e6.2272805210.1016/j.fertnstert.2012.05.035PMC35028662

Harada
T
, 
Taniguchi
F
, 
Amano
H
, 
Kurozawa
Y
, 
Ideno
Y
, 
Hayashi
K
, et al. Adverse obstetrical outcomes for women with endometriosis and adenomyosis: a large cohort of the Japan environment and Children's study. PLoS One. 2019;14:e0220256.3137408510.1371/journal.pone.0220256PMC66773023

Horton
J
, 
Sterrenburg
M
, 
Lane
S
, 
Maheshwari
A
, 
Li
TC
, 
Cheong
Y
. Reproductive, obstetric, and perinatal outcomes of women with adenomyosis and endometriosis: a systematic review and meta‐analysis. Hum Reprod Update. 2019;25:592–632.3131842010.1093/humupd/dmz0124

Nagai
K
, 
Hayashi
K
, 
Yasui
T
, 
Katanoda
K
, 
Iso
H
, 
Kiyohara
Y
, et al. Disease history and risk of comorbidity in women's life course: a comprehensive analysis of the Japan Nurses' health study baseline survey. BMJ Open. 2015;5:e006360.10.1136/bmjopen-2014-006360PMC4360787257622305

Mu
F
, 
Rich‐Edwards
J
, 
Rimm
EB
, 
Spiegelman
D
, 
Missmer
SA
. Endometriosis and risk of coronary heart disease. Circ Cardiovasc Qual Outcomes. 2016;9:257–64.2702592810.1161/CIRCOUTCOMES.115.002224PMC49401266

Dunselman
GA
, 
Vermeulen
N
, 
Becker
C
, 
Calhaz‐Jorge
C
, 
D'Hooghe
T
, 
de Bie
B
, et al. ESHRE guideline: management of women with endometriosis. Hum Reprod. 2014;29:400–12.2443577810.1093/humrep/det4577
Japan Society of Obstetrics and Gynecology and Japan Association of Obstetricians and Gynecologists
, Guidelines for obstetric and gynecological practice in Japan: Guideline for gynecological practice in Japan 2020 [in Japanese]. Japan Society of Obstetrics and Gynecology, Tokyo, 2020.8

Vercellini
P
, 
DE Matteis
S
, 
Somigliana
E
, 
Buggio
L
, 
Frattaruolo
MP
, 
Fedele
L
. Long‐term adjuvant therapy for the prevention of postoperative endometrioma recurrence: a systematic review and meta‐analysis. Acta Obstet Gynecol Scand. 2013;92:8–16.2264629510.1111/j.1600-0412.2012.01470.x9

Takamura
M
, 
Koga
K
, 
Osuga
Y
, 
Takemura
Y
, 
Hamasaki
K
, 
Hirota
Y
, et al. Post‐operative oral contraceptive use reduces the risk of ovarian endometrioma recurrence after laparoscopic excision. Hum Reprod. 2009;24:3042–8.1968404510.1093/humrep/dep29710

Kitawaki
J
, 
Ishihara
H
, 
Kiyomizu
M
, 
Honjo
H
. Maintenance therapy involving a tapering dose of danazol or mid/low doses of oral contraceptive after gonadotropin‐releasing hormone agonist treatment for endometriosis‐associated pelvic pain. Fertil Steril. 2008;89:1831–5.1776117810.1016/j.fertnstert.2007.05.05211

Harada
T
, 
Momoeda
M
, 
Taketani
Y
, 
Hoshiai
H
, 
Terakawa
N
. Low‐dose oral contraceptive pill for dysmenorrhea associated with endometriosis: a placebo‐controlled, double‐blind, randomized trial. Fertil Steril. 2008;90:1583–8.1816400110.1016/j.fertnstert.2007.08.05112

Harada
T
, 
Momoeda
M
, 
Taketani
Y
, 
Aso
T
, 
Fukunaga
M
, 
Hagino
H
, et al. Dienogest is as effective as intranasal buserelin acetate for the relief of pain symptoms associated with endometriosis‐‐a randomized, double‐blind, multicenter, controlled trial. Fertil Steril. 2009;91:675–81.1865318410.1016/j.fertnstert.2007.12.08013

Trivedi
P
, 
Selvaraj
K
, 
Mahapatra
PD
, 
Srivastava
S
, 
Malik
S
. Effective post‐laparoscopic treatment of endometriosis with dydrogesterone. Gynecol Endocrinol. 2007;23(Suppl 1):73–6.1794354310.1080/0951359070166958314

Taniguchi
F
, 
Ota
I
, 
Iba
Y
, 
Toda
T
, 
Tagashira
Y
, 
Ohata
Y
, et al. The efficacy and safety of dydrogesterone for treatment of dysmenorrhea: an open‐label multicenter clinical study. J Obstet Gynaecol Res. 2019;45:168–75.3024627610.1111/jog.1380715

Tekin
BY
, 
Dilbaz
B
, 
Altinbas
SK
, 
Dilbaz
S
. Postoperative medical treatment of chronic pelvic pain related to severe endometriosis: levonorgestrel‐releasing intrauterine system versus gonadotropin‐releasing hormone analogue. Fertil Steril. 2011;95:492–6.2088399110.1016/j.fertnstert.2010.08.04216

Osuga
Y
, 
Seki
Y
, 
Tanimoto
M
, 
Kusumoto
T
, 
Kudou
K
, 
Terakawa
N
. Relugolix, an oral gonadotropin‐releasing hormone receptor antagonist, reduces endometriosis‐associated pain in a dose‐response manner: a randomized, double‐blind, placebo‐controlled study. Fertil Steril. 2021;115:397–405.3291263310.1016/j.fertnstert.2020.07.05517

Harada
T
, 
Osuga
Y
, 
Suzuki
Y
, 
Fujisawa
M
, 
Fukui
M
, 
Kitawaki
J
. Relugolix, an oral gonadotropin‐releasing hormone receptor antagonist, reduces endometriosis‐associated pain compared with leuprorelin in Japanese women: a phase 3, randomized, double‐blind, noninferiority study. Fertil Steril. 2022;117:583–92.3489570010.1016/j.fertnstert.2021.11.01318
Japan Society of Gynecologic and Obstetric Endoscopy and Minimally Invasive Therapy
, editor. Endometriosis. JSGOE guidelines for endoscopic surgery in obstetrics and gynecology 2019 [in Japanese]. Tokyo: KANEHARA & Co., LTD.; 2019. p. 42–62.19

Hart
RJ
, 
Hickey
M
, 
Maouris
P
, 
Buckett
W
, Cochrane Gynaecology and Fertility Group
. Excisional surgery versus ablative surgery for ovarian endometriomata. Cochrane Database Syst Rev. 2008;2:CD004992.10.1002/14651858.CD004992.pub31842590820

Iwase
A
, 
Hirokawa
W
, 
Goto
M
, 
Takikawa
S
, 
Nagatomo
Y
, 
Nakahara
T
, et al. Serum anti‐Müllerian hormone level is a useful marker for evaluating the impact of laparoscopic cystectomy on ovarian reserve. Fertil Steril. 2010;94:2846–9.2063050510.1016/j.fertnstert.2010.06.01021

Sutton
CJ
, 
Ewen
SP
, 
Whitelaw
N
, et al. Prospective, randomized, double‐blind, controlled trial of laser laparoscopy in the treatment of pelvic pain associated with minimal, mild, and moderate endometriosis. Fertil Steril. 1994;62:696–700.792607510.1016/s0015-0282(16)56990-822

Abbott
J
, 
Hawe
J
, 
Hunter
D
, et al. Laparoscopic excision of endometriosis: a randomized, placebo‐controlled trial. Fertil Steril. 2004;82:878–84.1548276310.1016/j.fertnstert.2004.03.04623

Koshiba
A
, 
Mori
T
, 
Okimura
H
, 
Akiyama
K
, 
Kataoka
H
, 
Takaoka
O
, et al. Dienogest therapy during the early stages of recurrence of endometrioma might be an alternative therapeutic option to avoid repeat surgeries. J Obstet Gynaecol Res. 2018;44:1970–6.2999267210.1111/jog.1372524

Rizk
B
, 
Fischer
AS
, 
Lotfy
HA
, 
Turki
R
, 
Zahed
HA
, 
Malik
R
, et al. Recurrence of endometriosis after hysterectomy. Facts Views Vis Obgyn. 2014;6:219–27.25593697PMC4286861

## Chapter 2

**Treatment flow chart**

1. **Pain**

Background of the treatment of endometriosis‐associated pain.

The chief clinical symptoms of endometriosis are pain, infertility, and ovarian endometriotic cysts. Pain is observed in approximately 80% of patients with endometriosis and may manifest earlier than other symptoms at any time from menarche or first intercourse.^1^ The pain interferes with studies, work, daily living, sexual relationship, and other aspects of life, thereby greatly deteriorating the quality of life of women.

Endometriosis is a chronic disease that is difficult to cure not only with medical therapy but also with surgical therapy. The primary objectives of treatment are to suppress pain, slow the disease progression, and preserve fertility. In the treatment of pain, the most suitable therapeutic strategy (medical therapy, surgical therapy, fertility treatment, or expectant management) should be selected on the basis of thorough informed consent from the patient.

For medical therapy, the 2014 ESHRE guideline^2^ recommends agents, such as NSAIDs, OCs/LEPs, progestins, and GnRH agonists to alleviate or eliminate pain, such as dysmenorrhea and dyspareunia.

In 2001, prior to the publication of the ESHRE guideline, Olive and Pritts^3^ proposed a medical therapy flowchart for pain in which they recommended NSAIDs or OCs/LEPs as the first line of treatment for endometriosis‐associated pain and GnRH agonists with add‐back therapy as an alternative choice when the first line of treatment is ineffective. Since then, other flowcharts, such as for pelvic pain and pain associated with deep endometriosis, have been reported.^4^, ^5^ However, there is currently scant evidence regarding the selection and usage of individual agents in hormone therapy or the order of choice of them. Hormone therapy will likely change in accordance with further clinical studies and the advent of novel agents arising from those studies.

On the other hand, recent results from clinical trials have demonstrated that while OCs/LEPs, progestins, and GnRH agonists are all effective for alleviating pain, and they are all equally effective.^6^, ^7^, ^8^ In addition, neither medical nor surgical therapy is statistically superior to the other.^9^ Therefore, in some cases, the selection of a treatment modality may be quite difficult.

However, these clinical situations actually demand familiarity with the characteristics of individual agents and surgical therapy as well as therapeutic strategies that are tailored to individual patients and account for differences in QOL assessments, which have clinical significance. For example, a particular consideration of the respective risks and benefits is required in the choice between surgical therapy, which can only be performed a certain number of times considering the preservation of the ovarian reserve and invasiveness, and long‐term medical therapy lasting from youth to adulthood.

In addition to comparisons between surgery and medical therapy and the superiority of the pain suppression effects among drug interactions, another important aspect in the choice between surgical therapy and medical therapy is the balance among surgical indication, choice of specific medication, adverse effects, and cost based on factors, such as whether and when individual patients desire to bear children, age, present symptom, the risk of complications, socioeconomic backgrounds, and changes in the QOL.

Therapeutic strategy for endometriosis associated pain.

**FIGURE 6:**
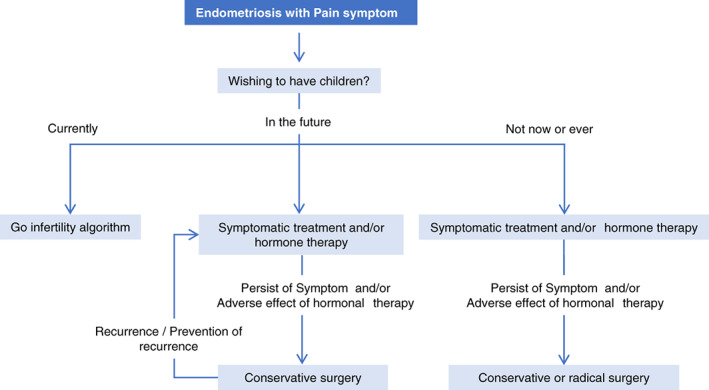
Treatment flowchart for patients with pain

Figure 6 shows a therapeutic strategy (flowchart) for pain symptom.

**FIGURE 7:**
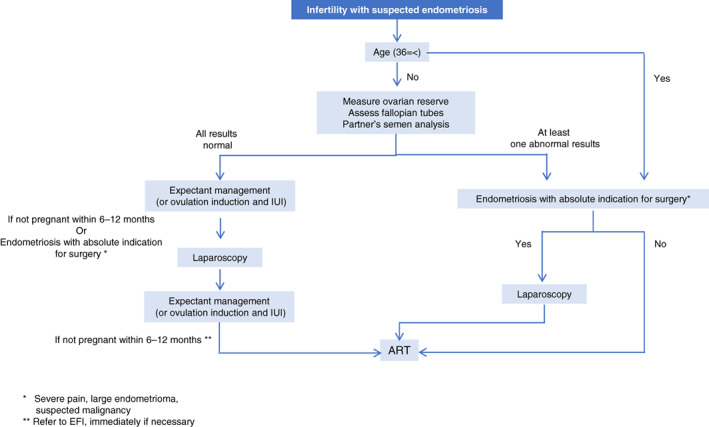
Treatment flowchart for patients with infertility. (Revised from Reference 6)

1. First, confirm whether the patient desires to bear children.
2. If the patient currently desires to bear children, proceed to the treatment according to a therapeutic strategy for infertile patients (Page 26, Figure 7) offering symptomatic treatment.
3. If the patient desires to bear children in the future but not currently, consider symptomatic treatment and/or hormone therapy. If symptoms persist or medical therapy yields adverse effects, consider conservative surgery. If symptoms or foci recur after conservative surgery, offer symptomatic treatment and/or hormone therapy. Also consider conducting hormone therapy to prevent recurrence after surgery.
4. If the patient has no desire to bear children in the future, perform symptomatic treatment and/or hormone therapy. If symptoms persist or medical therapy yields adverse effects, consider radical surgery.

References1

Sinaii
N
, 
Plumb
K
, 
Cotton
L
, 
Lambert
A
, 
Kennedy
S
, 
Zondervan
K
, et al. Differences in characteristics among 1,000 women with endometriosis based on extent of disease. Fertil Steril. 2008;89:538–45.1749871110.1016/j.fertnstert.2007.03.069PMC29399022

Dunselman
GA
, 
Vermeulen
N
, 
Becker
C
, 
Calhaz‐Jorge
C
, 
D'Hooghe
T
, 
de Bie
B
, et al. ESHRE guideline:management of women with endometriosis. Hum Reprod. 2014;29:400–12.2443577810.1093/humrep/det4573

Olive
DL
, 
Pritts
EA
. Treatment of endometriosis. N Engl J Med. 2001;345:266–75.1147466610.1056/NEJM2001072634504074

Speer
LM
, 
Mushkbar
S
, 
Erbele
T
. Chronic pelvic pain in women. Am Fam Physician. 2016;93:380–7.269269755

Vercellini
P
, 
Buggio
L
, 
Somigliana
E
. Role of medical therapy in the management of deep rectovaginal endometriosis. Fertil Steril. 2017;108:913–30.2920296510.1016/j.fertnstert.2017.08.0386

Shim
JY
, 
Laufer
MR
. Adolescent endometriosis:an update. J Pediatr Adolesc Gynecol. 2020;33:112–9.3181270410.1016/j.jpag.2019.11.0117

Bedaiwy
MA
, 
Allaire
C
, 
Yong
P
, 
Alfaraj
S
. Medical management of endometriosis in patients with chronic pelvic pain. Semin Reprod Med. 2017;35:38–53.2800285010.1055/s-0036-15973088

Vercellini
P
, 
Buggio
L
, 
Frattaruolo
MP
, 
Borghi
A
, 
Dridi
D
, 
Somigliana
E
. Medical treatment of endometriosis‐related pain. Best Pract Res Clin Obstet Gynaecol. 2018;51:68–91.2953042510.1016/j.bpobgyn.2018.01.0159

Chaichian
S
, 
Kabir
A
, 
Mehdizadehkashi
A
, 
Rahmani
K
, 
Moghimi
M
, 
Moazzami
B
. Comparing the efficacy of surgery and medical therapy for pain management in endometriosis:a systematic review and meta‐analysis. Pain Physician. 2017;20:185–95.28339429

2. **Infertility**

**Management of endometriosis‐associated infertility**

Endometriosis negatively affects fertility in both mechanically and biochemically. Mechanical mechanisms include impairment of fallopian tube oocyte capture associated with adhesion, and impairment of oocyte and sperm transport associated with abnormal uterine contraction. Biochemical mechanisms include abnormalities in the immune and endocrine environments in the peritoneal cavity, fallopian tube, ovaries, and uterus, which affect oocytes/sperms and embryos. Therefore, the control of endometriosis with surgery or medical treatment may inhibit these negative effects and thus improve fertility. ART also helps to improve fertility by bypassing the deteriorated pelvic milieu.

**Expectant management**

In women without notable pain and with normal findings of the partner's semen, expectant management with timed sex for approximately 12 months is not unreasonable, especially for young patients with only a short period of infertility with suspected minimal/mild endometriosis.

**Surgical therapy**

Surgical therapy potentially improves fertility by eliminating the mechanical factors described above through adhesiolysis, or by eliminating biochemical factors through peritoneal lavage and removal of lesions. However, surgery may not completely remove or repair these environments and might even harm reproductive function (i.e., reducing ovarian reserve) or decrease fertility by causing further adhesions. Therefore, estimating the efficacy of surgery requires balancing the benefits and risks of surgery and comparing it with alternative treatments, such as ART.

**Mild endometriosis**

It is well known that spontaneous pregnancy rates increase after laparoscopic surgery in patients with mild endometriosis. For this reason, guidelines in multiple countries recommend surgical therapy for mild endometriosis.^1^, ^2^, ^3^ However, the benefits of surgery do not necessarily outweigh those of ART as the presence of mild endometriosis cannot be determined until laparoscopy is performed, and there is the high risk and cost of surgery with respect to the pregnancy rate with ART.^4^ Taking into account the patient's age, symptoms, and social background, surgery should be planned when its benefits are evaluated to be relatively high, such as when improving pain is also an objective, or when the patient does not wish to undergo ART.

**Moderate/severe endometriosis**

Data on whether surgery for moderate/severe endometriosis helps to improve postoperative fertility have not been assessed in an RCT or elsewhere. However, compared to mild endometriosis, laparoscopic surgery has been shown to be less effective in improving postoperative fertility in patients with moderate/severe endometriosis, such as those with severe adhesions of the fallopian tubes. Therefore, ART should be considered for patients with moderate/severe endometriosis if the only goal is to improve fertility. However, surgery is recommended if it is deemed to be required for endometriosis itself, such as for relief from symptoms and pathological diagnosis.

Endometriosis fertility index (EFI).

The endometriosis fertility index (EFI) (See Reference 5, Page 1611) is a score that includes parameters such as the age of the patient, duration of infertility, pregnancy history, severity of endometriosis according to the r‐ASRM score, and the tubal, fimbrial, and ovarian appearance. This index is excellent for predicting postoperative spontaneous pregnancy rates and is useful for postoperative fertility treatment planning.^5^

This score has been shown to be reproducible and highly correlated with the pregnancy rate, making it useful when planning to switch to ART after surgery. Cumulative pregnancy rates at 1 year after operation with scores of 5, 6, and 7 or 8 are approximately 20%, 30%, and 40%, respectively (See Reference 5, Page 1611).

**General fertility treatment (non‐assisted reproductive technology, ovulation induction and/or intrauterine insemination)**

There is evidence showing the efficacy of general fertility treatment (non‐assisted reproductive technology, induction of ovulation, and/or artificial insemination) in patients with endometriosis, particularly in mild endometriosis. Therefore, this treatment is recommended in both ASRM^1^ and ESHRE^2^ guidelines (notably, the ESHRE guideline does not recommend treatment with artificial insemination alone). However, the following points should be kept in mind: the efficacy of these treatments in patients with endometriosis is lower than that in infertile patients without endometriosis; if pregnancy is not achieved during the treatment period, the patient will age for that period and endometriosis will progress further; and ovulation induction may further exacerbate endometriosis. Caution must be taken to avoid continuing general reproductive therapy without planning.

**Assisted reproductive technology (ART)**

ART yields the highest pregnancy rates per period and the shortest time to conception among any fertility treatment. Stepping up to ART should be considered according to age, duration of infertility, pain, treatment history, ovarian reserve, recurrence of endometriosis, and other infertility‐related factors.

**Therapeutic strategy for patients with endometriosis‐associated infertility**

The therapeutic strategy (flowchart) for patients with endometriosis‐associated infertility is shown in Figure 7.

In dealing with infertile couples suspected of endometriosis, the patient's age, ovarian reserve, tubal function, and partner's semen findings should be assessed first.

If the patient is older (age ≥ 36 years) or if at least one factor (ovarian reserve, tubal function, partner's semen findings) is abnormal, ART should be considered unless there is an indication for surgery for endometriosis itself. Laparoscopic surgery should be considered in cases of deep endometriosis with severe pain or large ovarian endometriotic cysts with a risk of rupture or difficulties in oocyte retrieval, or when malignancy cannot be ruled out. However, when planning surgery, it is necessary to consider its adverse effects, such as reduced ovarian reserve and complications.

If the patient is young (age ≤ 35 years) and has high ovarian reserve, and there is no severely impaired tubal function, with normal semen findings in the partner, pregnancy independent of ART can be expected. Therefore, expectant management or ovulation induction and intrauterine insemination can be considered. If pregnancy is not achieved within 12 months, laparoscopic surgery should be considered. However, even in these cases, surgery should be considered immediately if endometriosis with an absolute indication for surgery (such as a large ovarian endometriotic cyst) is present. After surgery, expectant management or ovulation induction and intrauterine insemination should be recommended for up to 12 months. If pregnancy is not achieved by then, ART is recommended. In cases such as where tubal function is determined to be severely impaired in laparoscopy, ART immediately after surgery should be considered according to factors, such as the EFI score.

References1
Practice Committee of the American Society for Reproductive Medicine
. Endometriosis and infertility: a committee opinion. Fertil Steril. 2012;98:591–8.2270463010.1016/j.fertnstert.2012.05.0312

Dunselman
GA
, 
Vermeulen
N
, 
Becker
C
, 
Calhaz‐Jorge
C
, 
D'Hooghe
T
, 
de Bie
B
, et al. ESHRE guideline: management of women with endometriosis. Hum Reprod. 2014;29:400–12.2443577810.1093/humrep/det4573

Kho
RM
, 
Andres
MP
, 
Borrelli
GM
, 
Neto
JS
, 
Zanluchi
A
, 
Abrão
MS
. Surgical treatment of different types of endometriosis: comparison of major society guidelines and preferred clinical algorithms. Best Pract Res Clin Obstet Gynaecol. 2018;51:102–10.2954511410.1016/j.bpobgyn.2018.01.0204

Tanbo
T
, 
Fedorcsak
P
. Endometriosis‐associated infertility: aspects of pathophysiological mechanisms and treatment options. Acta Obstet Gynecol Scand. 2017;96:659–67.2799800910.1111/aogs.130825

Adamson
GD
, 
Pasta
DJ
. Endometriosis fertility index: the new, validated endometriosis staging system. Fertil Steril. 2010;94:1609–15.1993107610.1016/j.fertnstert.2009.09.0356

Zondervan
KT
, 
Becker
CM
, 
Koga
K
, 
Missmer
SA
, 
Taylor
RN
, 
Viganò
P
. Endometriosis. Nat Rev Dis Primers. 2019;4:9.10.1038/s41572-018-0008-530026507

## Chapter 3

**Treatment guidelines (Clinical questions)**

Reference selection and clinical question (CQ) assessment.

The detailed procedure used for the literature search is described below.

The committee members and authors selected the keywords and the main papers associated with the CQs. To assess the evidence at the present stage from an impartial perspective, we requested the Japan Medical Library Association to conduct a comprehensive literature search by creating different queries for each CQ. When a query failed to yield enough papers, we changed or added keywords. The committee members and authors examined the sampled papers and selected important papers.

The PubMed, Cochrane Library, and Ichushi Web databases were searched for studies published from 2010 to 2019.

The selection criteria were as follows: Practice guidelines, meta‐analyses, and systematic reviews were given the highest priority. Subsequently, RCT, prospective cohort studies, case–control studies, clinical trials, and other epidemiological studies were selected.

We also selected new papers (those published from January 2020 onwards), which the editing committee determined should be cited.

For external assessment, we presented some of the CQs at the consensus meeting at the 42nd Annual Conference of the Japan Society of Endometriosis.

The levels of evidence and the strengths of the recommendations were determined in accordance with the following criteria.

The committee members and the authors reached a consensus through discussion on the assessment of each CQ.

Evidence level

I Systematic Review/Meta‐analysis

II 1 or more RCTs

III Non‐RCT

IV Clinical Trial/Cohort Study

V Descriptive Study

VI Not based on patient data, or opinion of expert panel or individual expert

Strength of recommendation.

A Strongly recommended

B Recommended

C Recommended, but no clear evidence

D Not recommended

## CQ 1 How should adolescents with suspected endometriosis be treated?

1. Prescribe analgesics and hormone preparations (OCs/LEPs, progestins).

Evidence level I

Strength of recommendation A

2. When analgesics and hormone preparations are ineffective or when obstruction is presented in reproductive tract, perform surgery.

Evidence level III

Strength of recommendation C

3. Prescribe hormone preparations after surgery to prevent the progression and recurrence of endometriosis.

Evidence level III

Strength of recommendation C

Numbers of studies referenced

Practice Guidelines 3.

Systematic Reviews 5.

RCTs 18

Commentary

Although functional dysmenorrhea is considered common in adolescent dysmenorrhea, it is crucial to differentiate it from organic dysmenorrhea. The most common cause of dysmenorrhea is endometriosis. Laparoscopic examination in adolescents with chronic pelvic pain and dysmenorrhea reveals endometriosis in at least two‐thirds of patients.^1^ For patients with chronic pelvic pain and dysmenorrhea, although macroscopic and histological diagnosis via laparoscopy is considered invalid from risk–benefit and cost–benefit standpoints, withholding medical therapy for adolescent endometriosis may lead to the worsening of the disease.^2^

Therefore, diagnostic treatment should be initiated with hormone therapy using OCs/LEPs or progestins not only when endometriosis is clinically diagnosed with specific methods, such as ultrasonography but also when endometriosis is suspected even in the absence of evident findings of the disease, in addition to symptomatic treatment with analgesics.^3^ The 2020 Japan Society of Obstetrics and Gynecology OC/LEP guideline states that OCs/LEPs “can be started after menarche; however, their effects on bone growth and bone density must be considered.” The use of OCs/LEPs does not affect the epiphyseal closure; however, when OCs/LEPs are initiated, the potential slowing of bone mineral density acquisition requires consideration of the age at which menarche occurs; a uniform minimum age for the safe use of OCs/LEPs is difficult to establish.^4^ Non‐Japanese clinical studies similarly do not guarantee the safety of OCs/LEPs under 14 years of age.

When the above described medical therapy is ineffective, laparoscopic surgery is performed to diagnose endometriosis macroscopically and histologically, and ablation of the foci, adhesiolysis, and conservative surgery, such as removal of endometriotic cysts removal can be performed.^5^ However, unlike endometriosis in adults, adolescent endometriosis often involves clear or red lesions and thus requires careful observation.^6^ Furthermore, in surgery for adolescent endometriosis, the reduction in future fertility due to diminished ovarian reserve must be avoided as much as possible. Therefore, as stated in CQ8 and CQ9, surgery for ovarian endometriotic cysts requires greater caution.

Furthermore, when there is an obstruction in the reproductive tract, the cause of organic dysmenorrhea is often discovered after menarche, and increased reflux of menstrual blood into the peritoneal cavity often also results in endometriosis.^7^ Consequently, to prevent the occurrence and progression of endometriosis, surgery is required to at least alleviate menstrual blood reflux into the peritoneal cavity.

Endometriosis recurs frequently following conservative surgery. Preventing the recurrence and progression of endometriosis is particularly important in adolescents to prevent a reduction in fertility. The use of OCs/LEPs, progestins, and the LNG‐IUS (Mirena®) are recommended to prevent recurrence. Please refer to CQ23 and CQ24 for details.

References1

Janssen
EB
, 
Rijkers
ACM
, 
Hoppenbrouwers
K
, 
Meuleman
C
, 
D'Hooghe
TM
. Prevalence of endometriosis diagnosed by laparoscopy in adolescents with dysmenorrhea or chronic pelvic pain: a systematic review. Hum Reprod Update. 2013;19:570–82.2372794010.1093/humupd/dmt0162

Unger
CA
, 
Laufer
MR
. Progression of endometriosis in non‐medically managed adolescents: a case series. J Pediatr Adolesc Gynecol. 2011;24:e21–3.2112689410.1016/j.jpag.2010.08.0023
Committee on Adolescent Health Care
. ACOG Committee opinion no. 760: dysmenorrhea and endometriosis in the adolescent. Obstet Gynecol. 2018;132:e249–58.3046169410.1097/AOG.00000000000029784
Japan Society of Obstetrics and Gynecology
. OC/LEP guideline 2020 [in Japanese]. Tokyo: Japan Society of Obstetrics and Gynecology; 2021.5
European Society of Human Reproduction and Embryology
. ESHRE guideline: management of women with endometriosis. Hum Reprod. 2014;29:400–12.2443577810.1093/humrep/det4576

Laufer
MR
. Helping “adult gynecologists” diagnose and treat adolescent endometriosis: reflections on my 20 years of personal experience. J Pediatr Adolesc Gynecol. 2011;24(5 suppl):s13–7.2185654510.1016/j.jpag.2011.07.0057

Dietrich
JE
, 
Millar
DM
, 
Quint
EH
. Obstructive reproductive tract anomalies. J Pediatr Adolesc Gynecol. 2014;27:396–402.2543870810.1016/j.jpag.2014.09.001

## CQ 2 How should ovarian endometriotic cysts be managed in view of their potential for malignant transformation?

1. Provide the patient with detailed information on the frequency and clinical characteristics of the malignant transformation of ovarian endometriotic cysts.

Evidence level I

Strength of recommendation A

2. Care should be taken toward the patients to not instill unnecessary anxiety about the malignant transformation, and a therapeutic strategy should be determined according to the menstrual status and the patient's desire to have children.

Evidence level I

Strength of recommendationA

3. In the follow‐up, a long‐term observation with ultrasound assessment of cyst size and characteristics of cyst contents should be performed.

Evidence level None

Strength of recommendation A

Number of studies referenced.

Meta‐analyses, Systematic Reviews, Practice guidelines 22.

Clinical trials 10.

Other epidemiologic studies 153.

Commentary.

A meta‐analysis reported in 2014 by Kim et al. on the risk and outcomes of ovarian cancer in patients with endometriosis (an analysis of 444 225 patients from 20 case–control studies and 15 cohort studies) showed that endometriosis increases the risk of ovarian cancer (relative ratio [RR] 1.27, 95% confidence interval [CI] 1.21–1.32).^1^ Furthermore, in comparisons of clinical findings between ovarian cancer arising from ovarian endometriotic cysts and non‐endometriosis‐associated ovarian cancer, stages I − II disease (RR 1.96, 95% CI 1.37–2.81), grade I disease (RR 1.32, 95% CI 1.15–1.51), nulliparity (RR 1.33, 95% CI 1.25–1.42), endometrioid carcinoma (RR 1.76, 95% CI 1.55–2.00), and clear cell carcinoma (RR 2.61, 95% CI 2.23–3.05) were all more common in ovarian endometriotic cysts; however, progression‐free survival and overall survival did not differ significantly.^1^ A meta‐analysis reported in 2016 by Wang et al. (12 case–control studies) similarly reported that endometriosis is associated with higher risk of ovarian cancer (OR 1.42, 95% CI 1.28–1.57).^2^ However, among women in the general population, the lifetime rate of developing ovarian cancer is only 1.3%; although this risk is increased by endometriosis, it is less than the risks of breast cancer, colon cancer, and lung cancer, leading to the opinion that patients should not be forced to worry excessively about developing ovarian cancer from endometriosis.^3^ The ESHRE guideline published in 2014 cites the following information about endometriosis and cancer overall: there is no evidence that endometriosis causes cancer, patients with endometriosis are not at an increased risk of cancer. In addition, cancers, such as ovarian cancer and non‐Hodgkin's lymphoma are slightly more common in patients with endometriosis. Therefore, the guidelines recommend that clinicians explain the incidence of only some cancers, such as ovarian cancer, in women with endometriosis.^4^

Recently, with the predominant view that endometriosis is a chronic inflammatory disease, novel discoveries at the molecular level have been accumulated according to the histological classification regarding the mechanism of malignant transformation of ovarian endometriotic cysts. In addition to DNA damage, mutation, and abnormal methylation caused by oxidative stress associated with iron metabolism in cysts, as well as gene mutations, such as ARID1A, PIK3CA, PTEN, and KRAS, research has increasingly defined the role of gene networks related to HNF1B, highly expressed in clear cell carcinoma and increased expression of estrogen receptors in endometrioid carcinoma.^5^, ^6^

Novel biomarkers and diagnostic imaging methods are currently being developed for the prevention and early detection of malignant transformation of ovarian endometriotic cysts. However, findings related to clinical management have not yielded any definitive proof that cystectomy is effective, and malignant transformation of recurrent ovarian endometriotic cyst has been reported.^7^ In addition, while OCs/LEPs reduce the overall risk of ovarian cancer, there is no evidence that they reduce the risk of malignant transformation of ovarian endometriotic cysts. There is also no clear evidence regarding the measurement of serum CA 125 levels. Therefore, regardless of which test method or treatment method is selected, a subsequent long‐term follow‐up is important. Ultrasound observation and findings, such as cyst wall hypertrophy and the presence of mural nodules and rapid cyst growth ≥7–8 cm are considered useful for the early detection of malignant transformation.^5^ The interim report of Japan Endometrioma Malignant Transformation Study (JEMS) 2020, which stated that ovarian cancer had developed in 24 of the 2900 patients enrolled in the study, cited as problems that patients had not undergone examination for long periods and patients were lost to follow‐up, indicating the difficulty of a long‐term follow‐up.^8^

References1

Kim
HS
, 
Kim
TH
, 
Chung
HH
, 
Song
YS
. Risk and prognosis of ovarian cancer in women with endometriosis: a meta‐analysis. Br J Cancer. 2014;110:1878–90.2451859010.1038/bjc.2014.29PMC39740762

Wang
C
, 
Liang
Z
, 
Liu
X
, 
Zhang
Q
, 
Li
S
. The association between endometriosis, tubal ligation, hysterectomy, and epithelial ovarian cancer: meta‐analyses. Int J Environ Res Public Health. 2016;13:1138.2785425510.3390/ijerph13111138PMC51293483

Kvaskoff
M
, 
Horne
AW
, 
Missmer
SA
. Informing women with endometriosis about ovarian cancer risk. Lancet. 2017;390:2433–4.2920829910.1016/S0140-6736(17)33049-04

Dunselman
GAJ
, 
Vermeulen
N
, 
Becker
C
, 
Calhaz‐Jorge
C
, 
D'Hooghe
T
, 
de Bie
B
, et al. ESHRE guideline: management of women with endometriosis. Hum Reprod. 2014;29:400–12.2443577810.1093/humrep/det4575

Kobayashi
H
, 
Yamada
Y
, 
Kawahara
N
, 
Ogawa
K
, 
Yoshimoto
C
. Integrating modern approaches to pathogenetic concepts of malignant transformation of endometriosis. Oncol Rep. 2019;41:1729–38.3059228910.3892/or.2018.69466

Amano
Y
, 
Mandai
M
, 
Yamaguchi
K
, 
Matsumura
N
, 
Kharma
B
, 
Baba
T
, et al. Metabolic alterations caused by HNF1β expression in ovarian clear cell carcinoma contribute to cell survival. Oncotarget. 2015;6:26002–17.2631829210.18632/oncotarget.4692PMC46948817

Haraguchi
H
, 
Koga
K
, 
Takamura
M
, 
Makabe
T
, 
Sue
F
, 
Miyashita
M
, et al. Development of ovarian cancer after excision of endometrioma. Fertil Steril. 2016;106:1432–7.2754388910.1016/j.fertnstert.2016.07.10778

Taniguchi
F
. Japan Endometrioma malignant transformation study (JEMS) interim report [in Japanese]. Journal of Japan Society of Endometriosis. 2020;41:88.

## CQ 3 Do endometriosis and adenomyosis increase the risk of obstetric complications?

1. Pregnant women who currently have or previously have had endometriosis are at an increased risk of obstetric complications, such as preterm delivery and placenta previa.

Evidence level I

Strength of recommendation None

2. Pregnant women with adenomyosis are at an increased risk of obstetric complications, specifically preterm delivery, low birth weight, and preeclampsia.

Evidence level I

Strength of recommendation None

Numbers of studies referenced.

Systematic Reviews 10.

Cohort Studies 9

Case Control Studies 3

Commentary.

In 2019, results for a cohort study were reported, using data from the Japan Environment and Children's Study conducted by the Japanese Ministry of the Environment. The study included 96 655 women with singleton pregnancies. Of the 3517 women with self‐reported previous endometriosis, 1884 (53.6%) women presented with obstetric complications, leading to the conclusion that endometriosis increases the risk of obstetric complications (odds ratio [OR] 1.32, 95% CI 1.23–1.41). When pregnancies achieved with ART, which has been indicated to increase the risk of obstetric complications, were excluded, women with previous endometriosis demonstrated increased risk of preterm premature rupture of membranes (OR 1.62, 95% CI 1.27–2.08) and placenta previa (OR 2.87, 95% CI 2.19–3.75). However, there were no increased risks of hypertensive disorders in pregnancy (HDP), gestational diabetes mellitus (GDM), placental abruption, or fetal growth restriction.^1^

A systematic review and meta‐analysis of 104 papers by Horton et al. reported in 2019 found that compared to pregnancies without endometriosis, pregnancies with endometriosis showed increased risks of preterm delivery (OR 1.38, 95% CI 1.01–1.89), cesarean section (OR 1.98, 95% CI 1.64–2.38), placental abruption (OR 1.87, 95% CI 1.65–2.13), and admission to NICU (OR 1.29, 95% CI 1.07–1.55), but did not show increased risks of HDP, GDM, or low birth weight.^2^

A systematic review and meta‐analysis reported by Lalani et al. in 2018, with 33 articles on pregnancy with endometriosis, demonstrated that even when subjects were limited to those who spontaneously conceived, endometriosis was associated with increased risks of preterm delivery (OR 1.70, 95% CI 1.38–2.10), placenta previa (OR 6.83, 95% CI 2.10–22.24), cesarean section (OR 1.76, 95% CI 1.51–2.06), and low birth weight (OR 1.52, 95% CI 1.13–2.05).^3^

None of the above large‐scale studies limited subjects to patients with pathologically confirmed endometriosis. In the Nurses' Health Study II, a large‐scale cohort study conducted in the United States, outcomes were reported in 2019 for 8875 of 196 722 subjects, who had previously been laparoscopically diagnosed with endometriosis. The study involved multivariate analyses that included not only the age at pregnancy, the time of cohort enrollment, and time from endometriosis diagnosis to pregnancy, but also other confounding variables, such as race, age at menarche, regularity of menstrual cycle, BMI, smoking status, alcohol intake, parity, and history of infertility. A history of endometriosis was shown to be associated with a higher risks of HDP (RR 1.30, 95% CI 1.16–1.45), GDM (RR 1.35, 95% CI 1.11–1.63), preterm delivery (RR 1.16, 95% CI 1.05–1.28), and low birth weight (RR 1.16, 95% CI 1.03–1.29).^4^

The association between endometriosis and obstetric complications specifically in women with deep endometriosis is unknown due to the absence of meta‐analysis.^2^, ^5^ Due to the lack of evidence, further study is also necessary to determine whether endometriosis treatment can reduce the incidence of obstetric complications.^2^ Obstetric complications must be kept in mind during the perinatal management for pregnant women with current or previous endometriosis.

The systematic review and meta‐analysis by Horton et al. cited above not only analyzed pregnancy with endometriosis, but also examined pregnancy with adenomyosis. Compared to healthy controls, pregnant women with adenomyosis (including pregnancies achieved with in vitro fertilization and embryo transfer [IVF‐ET]) demonstrated increased risks of preterm delivery (OR 2.74, 95% CI 1.89–3.97), low birth weight (OR 3.90, 95% CI 2.10–7.25), and preeclampsia (OR 7.87, 95% CI 1.26–49.2).^2^ Tan et al. conducted a systematic review of 18 studies on obstetric complications after conservative surgery for adenomyosis, which were examined separately according to the type of adenomyosis, such as focal and diffuse adenomyosis. In focal adenomyosis, although uterine rupture did not occur, preterm delivery occurred in 10.9% of cases (12/110); moreover, in diffuse adenomyosis, uterine rupture and preterm delivery were observed in 6.8% of patients (3/44) and 4.5% of patients (2/44), respectively.^6^ However, there are no reports of outcomes regarding the effects of pre‐pregnancy medical therapy in pregnant women with adenomyosis.

References1

Harada
T
, 
Taniguchi
F
, 
Amano
H
, 
Kurozawa
Y
, 
Ideno
Y
, 
Hayashi
K
, et al. Adverse obstetrical outcomes for women with endometriosis and adenomyosis: a large cohort of the Japan environment and Children's study. PLoS One. 2019;14:e0220256.3137408510.1371/journal.pone.0220256PMC66773022

Horton
J
, 
Sterrenburg
M
, 
Lane
S
, 
Maheshwari
A
, 
Li
TC
, 
Cheong
Y
. Reproductive, obstetric, and perinatal outcomes of women with adenomyosis and endometriosis: a systematic review and meta‐analysis. Hum Reprod Update. 2019;25:592–632.3131842010.1093/humupd/dmz0123

Lalani
S
, 
Choudhry
AJ
, 
Firth
B
, 
Bacal
V
, 
Walker
M
, 
Wen
SW
, et al. Endometriosis and adverse maternal, fetal, and neonatal outcomes, a systematic review and meta‐analysis. Hum Reprod. 2018;33:1854–65.3023973210.1093/humrep/dey269PMC61454204

Farland
LV
, 
Prescott
J
, 
Sasamoto
N
, 
Tobias
DK
, 
Gaskins
AJ
, 
Stuart
JJ
, et al. Endometriosis and risk of adverse pregnancy outcomes. Obstet Gynecol. 2019;134:527–36.3140358410.1097/AOG.0000000000003410PMC69220845

Nirgianakis
K
, 
Gasparri
ML
, 
Radan
AP
, 
Villiger
A
, 
McKinnon
B
, 
Mosimann
B
, et al. Obstetric complications after laparoscopic excision of posterior deep infiltrating endometriosis: a case‐control study. Fertil Steril. 2018;110:459–66.3009869810.1016/j.fertnstert.2018.04.0366

Tan
J
, 
Moriarty
S
, 
Taskin
O
, 
Allaire
C
, 
Williams
C
, 
Yong
P
, et al. Reproductive outcomes after fertility‐sparing surgery for focal and diffuse adenomyosis: a systematic review. J Minim Invasive Gynecol. 2018;25:608–21.2930523410.1016/j.jmig.2017.12.020

## CQ 4 How should adenomyosis‐associated pain be managed?

1.Medical therapy similar to that for endometriosis is effective and is to be considered.

Evidence level I

Strength of recommendation B

2. Uterine artery embolization, high‐intensity focused ultrasound, and conservative surgery for adenomyosis are suggested to be effective but require thorough informed consent.

Evidence level III

Strength of recommendation C

Numbers of studies referenced.

Systematic Reviews 11

Cohort Studies 1

Intervention Studies 13

Commentary.

Dysmenorrhea is observed in 50%–93% of patients with adenomyosis,^1^ and similar treatment selected. in cases with endometriosis is recommended.^2^ We found three systematic reviews related to the use of medications for the treatment for endometriosis‐associated pain.^1^, ^3^, ^4^ Along with the efficiency of GnRH agonists, norethisterone, danazol, dienogest, LNG‐IUS (Mirena®), OCs/LEPs, and NSAIDs, other agents under the investigation, such as aromatase inhibitors, selective progesterone receptor modulators, and GnRH antagonists have also been introduced. Few controlled trials have been conducted to provide evidence for the effects of the above agents, which have come mostly from single‐arm interventional studies.

An OC/LEP (ethinyl estradiol 30 μg plus gestodene 75 μg for 21 days followed by a 7‐day withdrawal period) demonstrated a reduction in pain 6 months after use compared to baseline (visual analog scale [VAS]: 6.5 → 3.9). An LNG‐IUS demonstrated a pain improvement not only similar to but superior to that of OCs/LEPs (VAS: 6.2 → 1.7).^5^ Following 12 weeks of oral letrozole (2.5 mg/day) or subcutaneous goserelin (3.6 mg/month), the improvement rates in pelvic pain and dysmenorrhea were 83.3% and 57.1%, respectively, in the letrozole group versus 92.8% and 100%, respectively, in the goserelin group; thus, the goserelin group demonstrated a greater improvement in pelvic pain.^6^

In one RCT, dienogest and a placebo were compared in patients with adenomyosis in which patients with a hemoglobin level <8.0 g/dl or muscle layer thickness ≥4 cm were excluded. After a period of 16 weeks, the dienogest group demonstrated significant reductions in the pain score, pain severity score, analgesic use score, and VAS.^7^ In another trial, dienogest (2 mg/day) and triptorelin (3.75 mg/4 weeks) were equally effective in reducing dyspareunia and chronic pelvic pain. At 16 weeks, triptorelin was more effective in improving dysmenorrhea (VAS: 30.6 vs. 0).^8^

Although uterine artery embolization, high‐intensity focused ultrasound therapy (HIFU), and conservative surgery have been reported as non‐medical conservative therapies for pain, all of these studies were single‐arm studies. According to a systematic review and meta‐analysis of the effects of uterine artery embolization, during an assessment at <12 months, an improvement in dysmenorrhea was observed in 89.6% of patients with pure adenomyosis versus 94.3% of patients with adenomyosis with fibroids; while at the ≥12 months assessment, an improvement in dysmenorrhea was observed in 74.0% of patients with adenomyosis alone versus 84.5% of patients with adenomyosis with fibroids.^9^ In a systematic review of HIFU, a reduction in dysmenorrhea was observed in 25%–83.3% of patients at 3 months, in 44.7%–100% of patients at 6 months, and in 64%–72.1% of patients at 12 months.^10^ In a systematic review and meta‐analysis that examined conservative surgical therapy in 1049 patients across 64 studies, a reduction in dysmenorrhea was observed in 82.0% of patients who underwent complete excision and 81.8% of patients who underwent partial excision.^11^

For patients with no desire to preserve their fertility, hysterectomy is considered as a definitive option.^1^, ^2^

References1

Benetti‐Pinto
CL
, 
Mira
TAA
, 
Yela
DA
, 
Teatin‐Juliato
CR
, 
Brito
LGO
. Pharmacological treatment for symptomatic adenomyosis: a systematic review. Rev Bras Ginecol Obstet. 2019;41:564–74.3154627810.1055/s-0039-16957372
Japan Society of Obstetrics and Gynecology and Japan Association of Obstetricians and Gynecologists
. Guidelines for obstetric and gynecological practice in Japan: guideline for gynecological practice in Japan 2017 [in Japanese]. Tokyo: Japan Society of Obstetrics and Gynecology; 2017.3

Vannuccini
S
, 
Luisi
S
, 
Tosti
C
, 
Sorbi
F
, 
Petraglia
F
. Role of medical therapy in the management of uterine adenomyosis. Fertil Steril. 2018;109:398–405.2956685210.1016/j.fertnstert.2018.01.0134

Pontis
A
, 
D'Alterio
MN
, 
Pirarba
S
, 
de Angelis
C
, 
Tinelli
R
, 
Angioni
S
. Adenomyosis: a systematic review of medical treatment. Gynecol Endocrinol. 2016;32:696–700.2737997210.1080/09513590.2016.11972005

Shaaban
OM
, 
Ali
MK
, 
Sabra
AM
, et al. Levonorgestrel‐releasing intrauterine system versus a low‐dose combined oral contraceptive for treatment of adenomyotic uteri: a randomized clinical trial. Contraception. 2015;92:301–7.2607167310.1016/j.contraception.2015.05.0156

Badawy
AM
, 
Elnashar
AM
, 
Mosbah
AA
. Aromatase inhibitors or gonadotropin‐releasing hormone agonists for the management of uterine adenomyosis: a randomized controlled trial. Acta Obstet Gynecol Scand. 2012;91:489–95.2222925610.1111/j.1600-0412.2012.01350.x7

Osuga
Y
, 
Fujimoto‐Okabe
H
, 
Hagino
A
. Evaluation of the efficacy and safety of dienogest in the treatment of painful symptoms in patients with adenomyosis: a randomized, double‐blind, multicenter, placebo‐controlled study. Fertil Steril. 2017;108:673–8.2891193410.1016/j.fertnstert.2017.07.0218

Fawzy
M
, 
Mesbah
Y
. Comparison of dienogest versus triptorelin acetate in premenopausal women with adenomyosis: a prospective clinical trial. Arch Gynecol Obstet. 2015;292:1267–71.2599048010.1007/s00404-015-3755-59

de Bruijn
AM
, 
Smink
M
, 
Lohle
PNM
, 
Huirne
JAF
, 
Twisk
JWR
, 
Wong
C
, et al. Uterine artery embolization for the treatment of adenomyosis: a systematic review and meta‐analysis. J Vasc Interv Radiol. 2017;28:1629–1642.e1.2903294610.1016/j.jvir.2017.07.03410

Cheung
VY
. Current status of high‐intensity focused ultrasound for the management of uterine adenomyosis. Ultrasonography. 2017;36:95–102.2814510910.14366/usg.16040PMC538184511

Grimbizis
GF
, 
Mikos
T
, 
Tarlatzis
B
. Uterus‐sparing operative treatment for adenomyosis. Fertil Steril. 2014;101:472–87.2428999210.1016/j.fertnstert.2013.10.025

## CQ 5 How should adenomyosis‐associated infertility be managed?

1. A higher likelihood of achieving pregnancy can be expected by GnRH agonists followed by ART

Evidence level III

Strength of recommendation C

2. An improvement of fecundability can be expected with conservative surgery for adenomyosis.

Evidence level III

Strength of recommendation C

Numbers of studies referenced.

Systematic Reviews 21

Case–Control Studies 7

Commentary.

Several systematic reviews have examined whether adenomyosis causes infertility. No study has examined whether untreated adenomyosis affects clinical pregnancy rates, live birth rates, or miscarriage rates in spontaneous pregnancy. Among patients who underwent deep endometriosis surgery, a comparison of pregnancy rates between patients with and without adenomyosis revealed that pregnancy rates were evidently lower among patients with adenomyosis (11.9% vs 43.0%, RR 0.32, 95% CI 0.16–0.66).^1^

An aggregation of the therapeutic outcomes in IVF‐ET for patients with adenomyosis revealed a clinical pregnancy rate of 36.1%, live birth rate of 29.9%, and miscarriage rate of 25.9%, all of which are favorable results.^2^ However, another study on adenomyosis showed a reduced clinical pregnancy rate (OR 0.57, 95% CI 0.43–0.76), reduced live birth rate (OR 0.45, 95% CI 0.24–0.86), and higher miscarriage rate (OR 3.49, 95% CI 1.41–8.65).^3^ These clinical results demonstrate that adenomyosis can adversely affect therapeutic outcomes in ART.

Two studies have assessed the effects of GnRH agonists before IVF in patients with adenomyosis. In a retrospective study that examined results in a group of patients who received goserelin (3.75 mg/month) for 2–3 months and underwent ovarian stimulation and oocyte retrieval following the conclusion of goserelin versus a group of patients who underwent the same procedures without GnRH agonist treatment, the number of retrieved oocytes was higher in the GnRH agonist pretreatment group (10.0 ± 8.2 vs. 7.9 ± 6.8).^4^ Another study compared clinical pregnancy rates, implantation rates, and ongoing pregnancy rates between a group of patients who underwent embryo transfer with hormone replacement therapy after receiving GnRH agonists and a group of patients who underwent the same procedure without GnRH agonists. In this study, the GnRH agonist group demonstrated a significantly higher clinical pregnancy rate (51.4% vs. 24.8%), implantation rate (32.6% vs. 16.1%), and ongoing pregnancy rate (48.9% vs. 21.4%) compared to those of patients without GnRH agonists.^5^ These outcomes suggest that pretreatment with GnRH agonists may be effective for achieving pregnancy.

Studies on the effects of conservative surgery for adenomyosis are as follows. Although the sample size was small, a comparison of the effects of conservative surgery combined with GnRH agonists versus GnRH agonists alone found that surgical therapy increased the rate of spontaneous pregnancy (OR 6.22, 95% CI 2.34–16.54).^6^ In a systematic review of the effects of conservative surgery, although the studies in question were single‐arm studies without controls, among a total of 1049 subjects in 64 studies, pregnancy rates after complete excision and partial excision of adenomyosis were 60.5% and 46.9%, respectively.^7^ A systematic review that examined the effects of conservative surgery on pregnancy rates in focal versus diffuse adenomyosis found that the pregnancy and miscarriage rates in patients with focal adenomyosis who underwent conservative surgery were 52.7% (95% CI 14.3–77.5) and 21.1% (95% CI 0–44.4), while these rates in patients with diffuse adenomyosis who underwent conservative surgery were 34.1% (95% CI 9.4–100) and 21.7% (95% CI 12.5–33.3), respectively.^8^

References1

Vercellini
P
, 
Consonni
D
, 
Barbara
G
, 
Buggio
L
, 
Frattaruolo
MP
, 
Somigliana
E
. Adenomyosis and reproductive performance after surgery for rectovaginal and colorectal endometriosis: a systematic review and meta‐analysis. Reprod Biomed Online. 2014;28:704–13.2474583110.1016/j.rbmo.2014.02.0062

Rocha
TP
, 
Andres
MP
, 
Borrelli
GM
, 
Abrão
MS
. Fertility‐sparing treatment of adenomyosis in patients with infertility: a systematic review of current options. Reprod Sci. 2018;25:480–6.2940219910.1177/19337191187567543

Horton
J
, 
Sterrenburg
M
, 
Lane
S
, 
Maheshwari
A
, 
Li
TC
, 
Cheong
Y
. Reproductive, obstetric, and perinatal outcomes of women with adenomyosis and endometriosis: a systematic review and meta‐analysis. Hum Reprod Update. 2019;25:592–632.3131842010.1093/humupd/dmz0124

Park
CW
, 
Choi
MH
, 
Yang
KM
, 
Song
IO
. Pregnancy rate in women with adenomyosis undergoing fresh or frozen embryo transfer cycles following gonadotropin‐releasing hormone agonist treatment. Clin Exp Reprod Med. 2016;43:169–73.2768904010.5653/cerm.2016.43.3.169PMC50393105

Niu
Z
, 
Chen
Q
, 
Sun
Y
, 
Feng
Y
. Long‐term pituitary downregulation before frozen embryo transfer could improve pregnancy outcomes in women with adenomyosis. Gynecol Endocrinol. 2013;29:1026–30.2400690610.3109/09513590.2013.8249606

Younes
G
, 
Tulandi
T
. Effects of adenomyosis on in vitro fertilization treatment outcomes: a meta‐analysis. Fertil Steril. 2017;108:483–490.e3.2886554810.1016/j.fertnstert.2017.06.0257

Grimbizis
GF
, 
Mikos
T
, 
Tarlatzis
B
. Uterus‐sparing operative treatment for adenomyosis. Fertil Steril. 2014;101:472–87.2428999210.1016/j.fertnstert.2013.10.0258

Tan
J
, 
Moriarty
S
, 
Taskin
O
, 
Allaire
C
, 
Williams
C
, 
Yong
P
, et al. Reproductive outcomes after fertility‐sparing surgery for focal and diffuse adenomyosis: a systematic review. J Minim Invasive Gynecol. 2018;25:608–21.2930523410.1016/j.jmig.2017.12.020

## CQ 6 Is endometriosis a risk factor for cardiovascular events?

Endometriosis is suggested to be a risk factor for future angina, myocardial infarction, and cerebral infarction.

Evidence level III

Strength of recommendation None

Numbers of studies referenced

Systematic Reviews 1

Cohort Studies 2

Case–Control Studies 2

Commentary

Endometriosis is currently recognized as a chronic systemic inflammatory disease associated with oxidative stress. The pathological mechanism of endometriosis has been linked to atherosclerosis, which causes conditions such as unstable angina, myocardial infarction, and cerebral infarction.^1^, ^2^

The Japan Nurses' Health Study, a cross‐sectional study that enrolled approximately 50 000 participants between 2001 and 2007, showed that the OR for comorbid endometriosis in cerebral infarction is 2.10 (95% CI 1.15–3.85).^3^ According to the Nurses' Health Study II, a large prospective study conducted in the United States that was reported in 2016, patients surgically diagnosed with endometriosis were at risk of coronary heart disease until an age of 55 years; in particular, women with endometriosis aged ≤40 years were found to be 3.08 times more likely (95% CI 2.02–4.70) than women without endometriosis to suffer events, such as myocardial infarction and angina.^2^ However, the latter study stated that hysterectomy/oophorectomy explained 42% of the association between endometriosis and coronary heart disease. In other words, radical surgery (surgical therapy) performed on young patients with endometriosis may have increased their risk of coronary heart disease until the age of menopause.

A systematic review^4^ in 2019 that covered the above studies reported that among patients surgically diagnosed with endometriosis, the relative risks of atherosclerotic cardiovascular disease were the following: 1.52 for myocardial infarction (95% CI 1.17–1.98), 1.91 for angina (1.59–2.29), and 1.35 for coronary artery bypass graft surgery (1.08–1.69). This review also reported a 1.25‐fold risk of hypercholesterolemia (1.21–1.30) and a 1.14‐fold risk of hypertension (1.09–1.18).

Studies on endometriosis and atherosclerotic lesions have been reported as follows. In two studies that examined flow‐mediated vasodilation (FMD), a marker of vascular endothelial function used for early detection of atherosclerosis, women with endometriosis demonstrated reduced FMD,^6^ suggesting vascular endothelial dysfunction. However, FMD is reversible. A 1% increase in FMD is associated with a 13% (95% CI 9–17) reduction in the risk of future cardiovascular events.^7^ Conversely, women with endometriosis show reduced FMD prior to surgical treatment, but one study reported a 5% increase in FMD 2 years after surgery, which is no longer different from controls.^8^

Additionally, while persistent vascular endothelial dysfunction can be evaluated with common carotid intima‐media thickness (ccIMT) 1–20 years before the onset of evident atherosclerosis,^9^ multiple studies have reported that ccIMT in women with endometriosis does not differ from ccIMT in women without endometriosis.^6^, ^10^ Conversely, a study measuring brachial‐ankle pulse wave velocity (baPWV), a marker of arteriosclerosis, found that baPWV was significantly higher in women aged ≥30 years with endometriosis than in women aged ≥30 years without endometriosis.^11^

Integrating the above studies yields the hypothesis that endometriosis patients already have functional vascular endothelial injury at a young age, which may contribute to an increased risk of future coronary heart disease.

The problem is how endometriosis treatment affects this risk. As stated above, while surgical therapy can improve FMD, radical surgery is indicated to increase the risk of coronary heart disease. Although early diagnosis of endometriosis and early initiation of medical therapy can help avoid the need for hysterectomy and oophorectomy, thus reducing the risk of coronary heart disease, the effects of individual forms of medical therapy are unknown.

Therefore, while it may be ideal to advise women with endometriosis, even if they are young, to continue with a lifestyle that reduces their risk of future coronary heart disease, there is not yet sufficient evidence to make that recommendation. More research is necessary to determine whether early diagnosis and treatment of endometriosis reduce the incidence of future cardiovascular events and what treatments reduce the risk of such events.

References1

Santoro
L
, 
D'Onofrio
F
, 
Flore
R
, 
Gasbarrini
A
, 
Santoliquido
A
. Endometriosis and atherosclerosis: what we already know and what we have yet to discover. Am J Obstet Gynecol. 2015;213:326–31.2593577710.1016/j.ajog.2015.04.0272

Mu
F
, 
Rich‐Edwards
J
, 
Rimm
EB
, 
Spiegelman
D
, 
Missmer
SA
. Endometriosis and risk of coronary heart disease. Circ Cardiovasc Qual Outcomes. 2016;9:257–64.2702592810.1161/CIRCOUTCOMES.115.002224PMC49401263

Nagai
K
, 
Hayashi
K
, 
Yasui
T
, et al. Disease history and risk of comorbidity in women's life course: a comprehensive analysis of the Japan Nurses' health study baseline survey. BMJ Open. 2015;5:e006360.10.1136/bmjopen-2014-006360PMC4360787257622304

Tan
J
, 
Taskin
O
, 
Iews
M
, 
Lee
AJ
, 
Kan
A
, 
Rowe
T
, et al. Atherosclerotic cardiovascular disease in women with endometriosis: a systematic review of risk factors and prospects for early surveillance. Reprod Biomed Online. 2019;39:1007–16.3173554910.1016/j.rbmo.2019.05.0215

Kinugasa
S
, 
Shinohara
K
, 
Wakatsuki
A
. Increased asymmetric dimethylarginine and enhanced inflammation are associated with impaired vascular reactivity in women with endometriosis. Atherosclerosis. 2011;219:784–8.2188031610.1016/j.atherosclerosis.2011.08.0056

Santoro
L
, 
D'Onofrio
F
, 
Campo
S
, 
Ferraro
PM
, 
Tondi
P
, 
Campo
V
, et al. Endothelial dysfunction but not increased carotid intima‐media thickness in young European women with endometriosis. Hum Reprod. 2012;27:1320–6.2241600910.1093/humrep/des0627

Inaba
Y
, 
Chen
JA
, 
Bergmann
SR
. Prediction of future cardiovascular outcomes by flow‐mediated vasodilatation of brachial artery: a meta‐analysis. Int J Cardiovasc Imaging. 2010;26:631–40.2033992010.1007/s10554-010-9616-18

Santoro
L
, 
D'Onofrio
F
, 
Campo
S
, 
Ferraro
PM
, 
Flex
A
, 
Angelini
F
, et al. Regression of endothelial dysfunction in patients with endometriosis after surgical treatment: a 2‐year follow‐up study. Hum Reprod. 2014;29:1205–10.2477784810.1093/humrep/deu0749

Bisoendial
RJ
, 
Hovingh
GK
, 
de Groot
E
, 
Kastelein
JJ
, 
Lansberg
PJ
, 
Stroes
ES
. Measurement of subclinical atherosclerosis: beyond risk factor assessment. Curr Opin Lipidol. 2002;13:595–603.1244188310.1097/00041433-200212000-0000210

Pretta
S
, 
Remorgida
V
, 
Abbamonte
LH
, 
Anserini
P
, 
Ragni
N
, 
del Sette
M
, et al. Atherosclerosis in women with endometriosis. Eur J Obstet Gynecol Reprod Biol. 2007;132:226–31.1668211210.1016/j.ejogrb.2006.04.01511

Tani
A
, 
Yamamoto
S
, 
Maegawa
M
, 
Kunimi
K
, 
Matsui
S
, 
Keyama
K
, et al. Arterial stiffness is increased in young women with endometriosis. J Obstet Gynaecol. 2015;35:711–5.2554352610.3109/01443615.2014.992871

## CQ 7 Does endometriosis affect quality of life?

Endometriosis negatively affects QOL. Surgical and medical treatment (surgery) may improve QOL.

Evidence level I

Strength of recommendation A

Numbers of studies referenced.

Systematic Reviews 19

Cohort studies 20

Case–Control Studies 10

Commentary.

Based on the recent concept of patient‐centered medicine, patient‐reported outcomes (PROs), which assess symptoms by having patients answer a questionnaire themselves, have been attracting attention. One such PRO is the Health‐related QOL (HR‐QOL), in which one assesses how the disease affects the patient's QOL. HR‐QOL includes comprehensive measures that enable comparisons with diseases in other fields and disease‐specific measures that enable the assessment of symptoms specific to each disease. HR‐QOL evaluation can also allow the assessment of disease‐related economic loss.

In a systematic review of 201 studies involving HR‐QOL evaluations among endometriosis patients, Bourdel et al. found that the comprehensive scales Short Form‐36 (SF‐36), EurQol 5 Dimension (EQ‐5D), and the disease‐specific scales Endometriosis Health Profile (EHP)‐30 and EHP‐5 were commonly used and were high‐quality methods to assess disease.^1^

The SF‐36 is a scale composed of eight subscales that assesses QOL in physical, social, and psychological dimensions. Among women with endometriosis, almost all eight subscales of the SF‐36 are reported to be statistically significantly lower than in women without endometriosis, signifying a tendency toward a diminished QOL.^2^, ^3^, ^4^, ^5^ Upon a comparison of SF‐36 scores among patients with rheumatoid arthritis, patients with endometriosis demonstrated statistically significantly lower scores on five of the eight subscales.^5^

The EQ‐5D is also used to examine economic effects. According to a study by Arakawa et al. that used data for Japanese people, endometriosis, and dysmenorrhea result in productivity losses of approximately 360 000 JPY per year per capita, meaning that proactive treatment with LEPs or dienogest, for example, could improve QOL relatively cheaply.^6^

In a systematic review of 30 articles on the treatment and improvement of HR‐QOL in endometriosis, Jia et al. found that GnRH agonist therapy, GnRH agonist + add‐back therapy, progestin therapy, and OC/LEP combination therapy all improve QOL, with no differences in improvement in QOL between hormone preparations.^7^ In a systematic review of 38 articles related to surgical therapy‐associated changes in QOL, Arcoverde et al. examined improvements in the Mental Component Score (MCS) and the Physical Component Score (PCS). Surgery to remove endometriosis significantly improved MCS but did not produce significant differences in PCS. In contrast, surgery for deep endometriosis and intestinal endometriosis was found to significantly improve both MCS and PCS.^8^

In Japan, there is a low quantity of data that evaluate the QOL of endometriosis patients based on SF‐36 or other similar measures. Several research questions remain to be addressed, including the examination of the minimally important difference that determines the degree of change in the SF‐36 scores that is clinically significant.

References1

Bourdel
N
, 
Chauvet
P
, 
Billone
V
, 
Douridas
G
, 
Fauconnier
A
, 
Gerbaud
L
, et al. Systematic review of quality of life measures in patients with endometriosis. PLoS One. 2019;14:e0208464.3062959810.1371/journal.pone.0208464PMC63281092

Petrelluzzi
KFS
, 
Garcia
MC
, 
Petta
CA
, 
Grassi‐Kassisse
DM
, 
Spadari‐Bratfisch
RC
. Salivary cortisol concentrations, stress, and quality of life in women with endometriosis and chronic pelvic pain. Stress. 2008;11:390–7.1880031010.1080/102538907018406103

Nunes
FR
, 
Ferreira
JM
, 
Bahamondes
L
. Prevalence of fibromyalgia and quality of life in women with and without endometriosis. Gynecol Endocrinol. 2014;30:307–10.2441033310.3109/09513590.2013.8764014

Lövkvist
L
, 
Boström
P
, 
Edlund
M
, 
Olovsson
M
. Age‐related differences in quality of life in Swedish women with endometriosis. J Womens Health (Larchmt). 2016;25:646–53.2678898210.1089/jwh.2015.54035

Verket
NJ
, 
Uhlig
T
, 
Sandvik
L
, 
Andersen
MH
, 
Tanbo
TG
, 
Qvigstad
E
. Health‐related quality of life in women with endometriosis, compared with the general population and women with rheumatoid arthritis. Acta Obstet Gynecol Scand. 2018;97:1339–48.3000708010.1111/aogs.134276

Arakawa
I
, 
Momoeda
M
, 
Osuga
Y
, 
Ota
I
, 
Koga
K
. Cost‐effectiveness of the recommended medical intervention for the treatment of dysmenorrhea and endometriosis in Japan. Cost Eff Resour Alloc. 2018;16:12.2964374410.1186/s12962-018-0097-8PMC58918937

Jia
SZ
, 
Leng
JH
, 
Shi
JH
, 
Sun
PR
, 
Lang
JH
. Health‐related quality of life in women with endometriosis: a systematic review. J Ovarian Res. 2012;5:29.2307881310.1186/1757-2215-5-29PMC35077058

Arcoverde
FVL
, 
Andres
MP
, 
Borrelli
GM
, et al. Surgery for endometriosis improves major domains of quality of life: a systematic review and meta‐analysis. J Minim Invasive Gynecol. 2019;26:266–78.3024415310.1016/j.jmig.2018.09.774

## CQ 8 What to e Aware of when Performing Conservative Surgery for Ovarian Endometriotic Cyst?

1. Cystectomy is superior to ablation/vaporization in terms of postoperative pregnancy and recurrence; however, be aware that cystectomy may decrease the ovarian reserve.

Evidence level I

Strength of recommendation A

2. Care must be taken to minimize ovarian damage when performing hemostasis at cystectomy.

Evidence level I

Strength of recommendation B

Numbers of studies referenced

Systematic Reviews 4

RCTs 4

Prospective cohort Studies 20

Commentary.

Since the early 2000s, studies have indicated that the number of oocytes retrieved in IVF/intracytoplasmic sperm injection (ICSI) treatment cycles may decrease following surgery for ovarian endometriotic cysts. In 2010, Iwase et al. reported that serum levels of anti‐Müllerian hormone (AMH), an indicator of ovarian reserve, decrease after ovarian cystectomy and that this tendency is exaggerated in bilateral cystectomy.^1^, ^2^ A subsequent study comparing AMH levels before and after surgery, and antral follicle count (AFC), another indicator of ovarian reserve, between the affected ovary and the contralateral ovary^3^ demonstrated that ovarian reserve decreases after cystectomy. A 2012 meta‐analysis of 237 patients from seven prospective cohort studies and one RCT reported that the mean preoperative mean AMH of the patients (including cases of unilateral and bilateral cysts) was 3.0 ng/ml, while the mean postoperative difference (at the time of the last follow‐up in that study) difference [95% CI] was −1.13 ng/ml [−0.37, −1.88].^4^ Furthermore, a 2019 meta‐analysis of 12 prospective cohort studies (two of which were also included in the meta‐analysis discussed above) found that postoperative AMH levels were significantly lower in patients with bilateral ovarian endometriotic cysts than in patients with a unilateral ovarian endometriotic cyst at 1 week–1 month and 9–12 months post‐operation.^5^

Multiple retrospective studies and RCTs have examined hemostasis methods during cystectomy to prevent reductions in the ovarian reserve. Three meta‐analyses of these studies reported that nonthermal hemostasis methods (suture or hemostasis) were favorable according to postoperative AMH levels.^6^, ^7^, ^8^ One of these three meta‐analyses, which included three RCTs (105 patients), found that postoperative AMH levels were significantly lower in a bipolar electrocoagulation group than in nonthermal hemostasis.^7^

Two RCTs have compared cystectomy with ablation/vaporization in terms of ovarian reserve. In a study that compared cystectomy and bipolar ablation for patients with unilateral single ovarian endometriotic cysts, a subgroup analysis of cyst diameter ≥5 cm versus <5 cm showed that AMH levels at post‐operative 3 months were significantly reduced in both cystectomy and bipolar ablation. A comparison between cystectomy and bipolar ablation showed that postoperative AMH levels were significantly reduced in cystectomy, but only in the ≥5 cm subgroup.^9^ An RCT, which compared cystectomy and CO_2_ fiber laser vaporization for unilateral and bilateral endometriotic cysts found that AMH levels at post‐operative 3 months were significantly lower than at baseline, only in the cystectomy group.^10^ Although the above suggests that ablation/vaporization is superior in terms of maintaining ovarian reserve, these studies did not evaluate subsequent pregnancy or recurrence rates; therefore, the clinical interpretation of these studies requires caution. Furthermore, in a 2008 meta‐analysis, which compared cystectomy and ablation as surgical treatments for ovarian endometriotic cysts ≥3 cm, the postoperative spontaneous pregnancy rate, rate of recurrence of pain symptoms (including dysmenorrhea), and rate of ovarian endometriotic cysts recurrence were all superior in the cystectomy group (ovarian endometriotic cysts recurrence: OR 0.41, 95% CI 0.18–0.93).^11^

Based on the above, we recommend that conserving surgery for ovarian endometriotic cysts be considered on a case‐by‐case basis, such as the selection of ablation/vaporization in cases of recurrence and cases where the reduction of ovarian reserve needs to be avoided as much as possible, while keeping the possible reduction of ovarian reserve in mind and properly considering hemostasis and other operative procedures.

References1

Iwase
A
, 
Hirokawa
W
, 
Goto
M
, 
Takikawa
S
, 
Nagatomo
Y
, 
Nakahara
T
, et al. Serum anti‐Müllerian hormone level is a useful marker for evaluating the impact of laparoscopic cystectomy on ovarian reserve. Fertil Steril. 2010;94:2846–9.2063050510.1016/j.fertnstert.2010.06.0102

Hirokawa
W
, 
Iwase
A
, 
Goto
M
, 
Takikawa
S
, 
Nagatomo
Y
, 
Nakahara
T
, et al. The post‐operative decline in serum anti‐Mullerian hormone correlates with the bilaterality and severity of endometriosis. Hum Reprod. 2011;26:904–10.2129263910.1093/humrep/der0063

Muzii
L
, 
Di Tucci
C
, 
Di Feliciantonio
M
, 
Marchetti
C
, 
Perniola
G
, 
Panici
PB
. The effect of surgery for endometrioma on ovarian reserve evaluated by antral follicle count: a systematic review and meta‐analysis. Hum Reprod. 2014;29:2190–8.2508580010.1093/humrep/deu1994

Raffi
F
, 
Metwally
M
, 
Amer
S
. The impact of excision of ovarian endometrioma on ovarian reserve: a systematic review and meta‐analysis. J Clin Endocrinol Metab. 2012;97:3146–54.2272332410.1210/jc.2012-15585

Younis
JS
, 
Shapso
N
, 
Fleming
R
, 
Ben‐Shlomo
I
, 
Izhaki
I
. Impact of unilateral versus bilateral ovarian endometriotic cystectomy on ovarian reserve: a systematic review and meta‐analysis. Hum Reprod Update. 2019;25:375–91.3071535910.1093/humupd/dmy0496

Ata
B
, 
Turkgeldi
E
, 
Seyhan
A
, 
Urman
B
. Effect of hemostatic method on ovarian reserve following laparoscopic endometrioma excision: comparison of suture, hemostatic sealant, and bipolar dessication. A systematic review and meta‐analysis. J Minim Invasive Gynecol. 2015;22:363–72.2557318310.1016/j.jmig.2014.12.1687

Deckers
P
, 
Ribeiro
SC
, 
Simões
RDS
, 
CBDF
M
, 
Baracat
EC
. Systematic review and meta‐analysis of the effect of bipolar electrocoagulation during laparoscopic ovarian endometrioma stripping on ovarian reserve. Int J Gynaecol Obstet. 2018;140:11–7.2898031710.1002/ijgo.123388

Ding
W
, 
Li
M
, 
Teng
Y
. The impact on ovarian reserve of haemostasis by bipolar coagulation versus suture following surgical stripping of ovarian endometrioma: a meta‐analysis. Reprod Biomed Online. 2015;30:635–42.2591324710.1016/j.rbmo.2015.02.0129

Giampaolino
P
, 
Bifulco
G
, 
Di Spiezio
SA
, 
Mercorio
A
, 
Bruzzese
D
, 
Di Carlo
C
. Endometrioma size is a relevant factor in selection of the most appropriate surgical technique: a prospective randomized preliminary study. Eur J Obstet Gynecol Reprod Biol. 2015;195:88–93.2649216710.1016/j.ejogrb.2015.09.04610

Candiani
M
, 
Ottolina
J
, 
Posadzka
E
, 
Ferrari
S
, 
Castellano
LM
, 
Tandoi
I
, et al. Assessment of ovarian reserve after cystectomy versus ‘one‐step’ laser vaporization in the treatment of ovarian endometrioma: a small randomized clinical trial. Hum Reprod. 2018;33:2205–11.3029948210.1093/humrep/dey305PMC623836811

Hart
RJ
, 
Hickey
M
, 
Maouris
P
, 
Buckett
W
. Excisional surgery versus ablative surgery for ovarian endometriomata. Cochrane Database Syst Rev. 2008;2:CD004992.10.1002/14651858.CD004992.pub318425908

## CQ 9 Is surgical therapy for ovarian endometriotic cysts effective in improving fertility?

1. Postoperative spontaneous pregnancy rates are more favorable with cystectomy than with ablation.

Evidence level I

Strength of recommendation A

2. Surgical therapy for stage III/IV endometriosis may be effective in improving fertility.

Evidence level II

Strength of recommendation B

3. Cystectomy does not improve the pregnancy rate in IVF/ICSI.

Evidence level I

Strength of recommendation A

Number of studies referenced (Non‐ART pregnancy: ART pregnancy).

Systematic Reviews 2:7.

RCTs 5:2

Prospective cohort studies 2: 0.

Case–Control, Retrospective Studies 1: 18

Commentary

1. Cystectomy with the expectation of spontaneous postoperative pregnancy

A 2008 meta‐analysis comparing excision and ablation as surgical treatments for ovarian endometriotic cysts ≥3 cm concluded that excisional surgery was significantly more favorable in terms of postoperative spontaneous pregnancy in women who were infertile prior to surgery (OR 5.21, 95% CI 2.04–13.29).^1^ This meta‐analysis covered two RCTs. The following should be noted: these RCTs were conducted with relatively young women, with small sample sizes; the postoperative observation periods were 24 and 12 months; the numbers of patients (totals from both groups) were 64 and 100; and the mean ages ± standard deviation of the patients in the excision surgery group versus the ablation group were 29.1 ± 4.3 years vs. 30.2 ± 5.1 years and 28.4 ± 5.8 years vs. 28.5 ± 5.5 years, respectively.^2^, ^3^

A similar meta‐analysis published in 2013 compared cystectomy with coagulation or laser vaporization.^4^ This meta‐analysis selected the same two RCTs as the above meta‐analysis and reached the same conclusion regarding cystectomy versus coagulation. Only one RCT has compared cystectomy with laser vaporization.^5^ In this RCT, the mean ages ± SD of participants in the cystectomy group versus the laser vaporization group were 32.5 ± 6 years vs. 32.3 ± 5.9 years, respectively. At 12 months and 60 months of follow‐up, the cumulative pregnancy rates in the cystectomy group versus the laser vaporization group were 19.2% vs. 20.8% and 38.1% vs. 44.%, respectively; therefore, the differences between the groups were not significant. Although comparisons of ablation and laser vaporization versus cystectomy yielded different results, these differences may have arisen from differences in the ages of the participants.

2. Cystectomy for stage III/IV endometriosis and its impact on fertility

In a prospective cohort study, Nezhat et al. reported that in women with endometriosis and no other factors of infertility, postoperative pregnancy rates by stage of endometriosis (ASRM classification) were as follows: 72% in stage I, 70% in stage II, 67% in stage III and 69% in stage IV.^6^ In a similar study, Vercellini et al. reported postoperative pregnancy rates of 42% (stage I), 40% (stage II), 57% (stage III), and 52% (stage IV), respectively. In this study, the postoperative pregnancy rates in cases of peritoneal lesions only, concomitant unilateral ovarian endometriotic cysts, and concomitant bilateral ovarian endometriotic cysts were 40%, 54%, and 62%, respectively. According to a multivariate analysis, age was the only factor affecting postoperative pregnancy. Although no RCT has ever compared the fertility improvement achieved by surgical treatment for stage III/IV endometriosis of ASRM with an expectant management group, one study reported that pregnancy rates in expectant management for women with endometriosis and no other infertility factors were 25% for moderate endometriosis and 0% for severe endometriosis.^8^ Taken together, these results suggest that surgical therapy for stage III/IV endometriosis, which often involves ovarian endometriotic cysts, may be useful in improving fertility. However, there are no studies directly comparing the efficacy between surgery and ART for stage III/IV endometriosis, so surgery should be carefully planned for the sole purpose of improving fertility.

3. Cystectomy prior to ART

A 2010 meta‐analysis, which analyzed how ART outcomes are affected by the preART therapeutic intervention for endometriotic ovarian cysts, included one RCT on cystectomy.^9^ In this RCT, which compared 49 patients who underwent cystectomy prior to ICSI for ovarian endometriotic cysts with 60 patients who were treated expectantly (no surgery), the OR for the clinical pregnancy rate was 1.15 (95% CI 0.52–2.55); thus, there was no significant difference.^10^

Since then, several meta‐analyses on this issue have been reported. In a 2015 meta‐analysis, which included retrospective studies and RCTs, comparisons between a group of patients without surgical treatment and a group of patients who underwent surgery prior to IVF and/or ICSI revealed no significant differences in live birth or pregnancy rates. Although the number of oocytes retrieved did not differ significantly between groups, the surgical treatment group demonstrated a significant decrease in AFC and an increase in the FSH dose, suggesting effects on ovarian reserve and responsiveness.^11^

Two meta‐analyses published in 2017, which examined the outcomes of IVF/ICSI similarly found no significant differences in the live birth rate or pregnancy rate between a surgical treatment group and control group (no surgical treatment).^12,13^ However, one of these meta‐analyses reported a significant decrease in the number of oocytes retrieved in a cystectomy group.^13^

Another two meta‐analyses published in 2018 reported nearly identical results.^14^, ^15^ The above meta‐analyses selected several of the same papers and therefore reached similar conclusions. It should be noted that, at present, only two relevant RCTs have been reported.

Based on the above, surgical therapy for ovarian endometriotic cysts can be expected to improve fertility in patients who are reasonably likely to achieve postoperative spontaneous pregnancy. However, the selection of patients requires thorough consideration of their characteristics, such as their ages and other infertility factors.

References1

Hart
RJ
, 
Hickey
M
, 
Maouris
P
, 
Buckett
W
. Excisional surgery versus ablative surgery for ovarian endometriomata. Cochrane Database Syst Rev. 2008;2:CD004992.10.1002/14651858.CD004992.pub3184259082

Beretta
P
, 
Franchi
M
, 
Ghezzi
F
, 
Busacca
M
, 
Zupi
E
, 
Bolis
P
. Randomized clinical trial of two laparoscopic treatments of endometriomas: cystectomy versus drainage and coagulation. Fertil Steril. 1998;70:1176–80.984831610.1016/s0015-0282(98)00385-93

Alborzi
S
, 
Momtahan
M
, 
Parsanezhad
ME
, 
Dehbashi
S
, 
Zolghadri
J
, 
Alborzi
S
. A prospective, randomized study comparing laparoscopic ovarian cystectomy versus fenestration and coagulation in patients with endometriomas. Fertil Steril. 2004;82:1633–7.1558987010.1016/j.fertnstert.2004.04.0674

Dan
H
, 
Limin
F
. Laparoscopic ovarian cystectomy versus fenestration/coagulation or laser vaporization for the treatment of endometriomas: a meta‐analysis of randomized controlled trials. Gynecol Obstet Invest. 2013;76:75–82.2375125010.1159/0003511655

Carmona
F
, 
Martinez‐Zamora
MA
, 
Rabanal
A
, 
Martínez‐Román
S
, 
Balasch
J
. Ovarian cystectomy versus laser vaporization in the treatment of ovarian endometriotic cysts: a randomized clinical trial with a five‐year follow‐up. Fertil Steril. 2011;96:251–4.2157594110.1016/j.fertnstert.2011.04.0686

Nezhat
C
, 
Crowgey
S
, 
Nezhat
F
. Videolaseroscopy for the treatment of endometriosis associated with infertility. Fertil Steril. 1989;51:237–40.291277010.1016/s0015-0282(16)60483-17

Vercellini
P
, 
Fedele
L
, 
Aimi
G
, 
de Giorgi
O
, 
Consonni
D
, 
Crosignani
PG
. Reproductive performance, pain recurrence and disease relapse after conservative surgical treatment for endometriosis: the predictive value of the current classification system. Hum Reprod. 2006;21:2679–85.1679060810.1093/humrep/del2308

Olive
DL
, 
Stohs
GF
, 
Metzger
DA
, 
Franklin
RR
. Expectant management and hydrotubations in the treatment of endometriosis‐associated infertility. Fertil Steril. 1985;44:35–41.315959910.1016/s0015-0282(16)48674-79

Demirol
A
, 
Guven
S
, 
Baykal
C
, 
Gurgan
T
. Effect of endometrioma cystectomy on IVF outcome: a prospective randomized study. Reprod Biomed Online. 2006;12:639–43.1679011410.1016/s1472-6483(10)61192-310

Benschop
L
, 
Farquhar
C
, 
van der Poel
N
, 
Heineman
MJ
, Cochrane Gynaecology and Fertility Group
. Interventions for women with endometrioma prior to assisted reproductive technology. Cochrane Database Syst Rev. 2010;11:CD008571.10.1002/14651858.CD008571.pub2PMC116088152106970611

Hamdan
M
, 
Dunselman
G
, 
Li
TC
, 
Cheong
Y
. The impact of endometrioma on IVF/ICSI outcomes: a systematic review and meta‐analysis. Hum Reprod Update. 2015;21:809–25.2616879910.1093/humupd/dmv03512

Laursen
BJ
, 
Schroll
JB
, 
Macklon
KT
, 
Rudnicki
M
. Surgery versus conservative management of endometriomas in subfertile women. A systematic review. Acta Obstet Gynecol Scand. 2017;96:727–35.2842159910.1111/aogs.1315413

Tao
X
, 
Chen
L
, 
Ge
S
, 
Cai
L
. Weigh the pros and cons to ovarian reserve before stripping ovarian endometriomas prior to IVF/ICSI: a meta‐analysis. PLoS One. 2017;12:e0177426.2857499310.1371/journal.pone.0177426PMC545603314

Nickkho‐Amiry
M
, 
Savant
R
, 
Majumder
K
, 
Edi‐O'sagie
E
, 
Akhtar
M
. The effect of surgical management of endometrioma on the IVF/ICSI outcomes when compared with no treatment? A systematic review and meta‐analysis. Arch Gynecol Obstet. 2018;297:1043–57.2934484710.1007/s00404-017-4640-1PMC584966415

Wu
CQ
, 
Albert
A
, 
Alfaraj
S
, 
Taskin
O
, 
Alkusayer
GM
, 
Havelock
J
, et al. Live birth rate after surgical and expectant management of endometriomas after in vitro fertilization: a systematic review, meta‐analysis, and critical appraisal of current guidelines and previous meta‐analyses. J Minim Invasive Gynecol. 2019;26:299–311. E3.Commentary.3071786410.1016/j.jmig.2018.08.029

## CQ 10 Is surgical therapy for deep rectovaginal endometriosis effective for improving fertility?

Surgery for deep rectovaginal endometriosis may be effective in improving fertility.

Evidence level III

Strength of recommendation C

Numbers of studies referenced

Systematic Reviews 4

Cohort Studies 2

Case Control Studies 23

Practice Guidelines 2

Commentary

Deep rectovaginal endometriosis triggers severe dyspareunia, leading to a reduction in the frequency of intercourse. Furthermore, deep lesions are reported to negatively affect the ovarian reserve, and the number of oocytes recovered in IVF‐ET,^1^ possibly due to the exacerbation of the peritoneal environment in various forms, such as an increase in mediators that trigger inflammation.^2^ Although there are no studies with a high level of evidence on improving fertility through surgical treatment for deep rectovaginal endometriosis, a succession of recent studies has indicated the efficacy of surgical therapy in this regard.

In a prospective cohort study of 130 patients with deep endometriosis without involvement of the digestive or urinary tract, Vallée et al. reported a cumulative postoperative pregnancy rate of 53.7% at 3 years, 66% of which were spontaneous conceptions. The authors considered an increase in the frequency of intercourse associated with an improvement in dyspareunia to be a factor in this high postoperative spontaneous conception rate.^3^ In outcomes limited to women with infertility, spontaneous postoperative pregnancy was achieved in 70% of women with deep retrocervical endometriosis who underwent laparoscopic cul‐de‐sac dissection.^4^ Furthermore, a cumulative pregnancy rate of 39.5% was achieved among infertile women without ovarian endometriotic cysts after resection of deep endometriosis lesions.^5^ To limit cases to lesions infiltrating the uterosacral ligaments, where deep lesions are most found, patients without a cause of infertility for reasons other than endometriosis underwent laparoscopic lesion removal; at 1 year, 48.5% of patients had achieved spontaneous pregnancy.^6^ In another study, 32% of women with lesions in the rectovaginal septum achieved spontaneous pregnancy after conservative surgery.^7^ In a surgery to improve the intraperitoneal environment, postoperative pregnancy rates were higher in infertile women with deep lesions in several sites than at a single site^8^ and higher in women who underwent complete surgeries than in women who underwent incomplete surgeries.^9^ However, in a 2014 meta‐analysis of five studies by Vercellini et al., among women seeking pregnancy, the postoperative pregnancy rate after excision of deep lesions in the rectovaginal septum and colon was 43% (74/172) for women without adenomyosis, but only 11.9% (7/59) for women with adenomyosis. Thus, adenomyosis was associated with a 68% reduction in pregnancy rate, a fact that warrants caution.^10^

Regarding whether to perform deep endometriosis surgery prior to IVF‐ET, a study that prospectively compared patients who underwent deep endometriosis excision prior to IVF‐ET with patients who did not undergo excision found that pregnancy rates were significantly higher in the group that underwent excision.^11^ In an examination by lesion site, the pregnancy rates were significantly low among patients with rectouterine nodules.^12^ Conversely, in a cohort study of 177 patients with deep endometriosis, the outcomes of IVF‐ET did not differ between a group that underwent complete resection of the lesions, a group that underwent incomplete resection, and a group that underwent no surgery.^13^ As these divergent results show, the effect of removal of deep endometriosis lesions on IVF‐ET success rates remains open to debate; RCTs are needed to answer the question definitively.

This suggests that surgical therapy for deep rectovaginal endometriosis may help improve fertility. However, there is little evidence to suggest that surgery improves fertility in infertile women with deep endometriosis. A suitable treatment must be determined according to a comprehensive assessment that includes a clinical history, laboratory findings, pain symptoms, the risk of pregnancy complications, and the patient's wishes.^14^ Surgical therapy is likely indicated for patients with intense pain arising from deep lesions, patients with ovarian endometriotic cysts for which surgery is indicated, patients for whom IVF‐ET has failed repeatedly and patients who desire spontaneous pregnancy, among others.^15^ However, due to the possibility of urinary/intestinal complications even when this surgery is performed by experts, it should be performed by thoroughly experienced physicians in facilities where cooperation can be obtained from surgeons and others.^16^

References1

Papaleo
E
, 
Ottolina
J
, 
Viganò
P
, 
Brigante
C
, 
Marsiglio
E
, 
De Michele
F
, et al. Deep pelvic endometriosis negatively affects ovarian reserve and the number of oocytes retrieved for in vitro fertilization. Acta Obstet Gynecol Scand. 2011;90:878–84.2154280910.1111/j.1600-0412.2011.01161.x2

Harb
HM
, 
Gallos
ID
, 
Chu
J
, 
Harb
M
, 
Coomarasamy
A
. The effect of endometriosis on in vitro fertilisation outcome: a systematic review and meta‐analysis. BJOG. 2013;120:1308–20.2383450510.1111/1471-0528.123663

Vallée
A
, 
Ploteau
S
, 
Abo
C
, 
Stochino‐Loi
E
, 
Moatassim‐Drissa
S
, 
Marty
N
, et al. Surgery for deep endometriosis without involvement of digestive or urinary tracts: do not worry the patients!
Fertil Steril. 2018;109:1079–1085.e1.2993564410.1016/j.fertnstert.2018.02.1244

Reich
H
, 
McGlynn
F
, 
Salvat
J
. Laparoscopic treatment of cul‐de‐sac obliteration secondary to retrocervical deep fibrotic endometriosis. J Reprod Med. 1991;36:516–22.18348405

Shervin
A
, 
Mohazzab
A
, 
Aminlou
M
, 
Kamali
K
, 
Padmehr
R
, 
Shadjoo
K
, et al. Fertility outcome after laparoscopic treatment of advanced endometriosis in two groups of infertile patients with and without ovarian endometrioma. Eur J Obstet Gynecol Reprod Biol. 2016;201:46–50.2705496510.1016/j.ejogrb.2016.03.0096

Chapron
C
, 
Fritel
X
, 
Dubuisson
JB
. Fertility after laparoscopic management of deep endometriosis infiltrating the uterosacral ligaments. Hum Reprod. 1999;14:329–32.1009997310.1093/humrep/14.2.3297

Vercellini
P
, 
Pietropaolo
G
, 
De Giorgi
O
, 
Daguati
R
, 
Pasin
R
, 
Crosignani
PG
. Reproductive performance in infertile women with rectovaginal endometriosis: is surgery worthwhile?
Am J Obstet Gynecol. 2006;195:1303–10.1670707510.1016/j.ajog.2006.03.0688

Centini
G
, 
Afors
K
, 
Murtada
R
, 
Argay
IM
, 
Lazzeri
L
, 
Akladios
CY
, et al. Impact of laparoscopic surgical management of deep endometriosis on pregnancy rate. J Minim Invasive Gynecol. 2016;23:113–9.2642770310.1016/j.jmig.2015.09.0159

Stepniewska
A
, 
Pomini
P
, 
Bruni
F
, 
Mereu
L
, 
Ruffo
G
, 
Ceccaroni
M
, et al. Laparoscopic treatment of bowel endometriosis in infertile women. Hum Reprod. 2009;24:1619–25.1935713610.1093/humrep/dep08310

Vercellini
P
, 
Consonni
D
, 
Barbara
G
, 
Buggio
L
, 
Frattaruolo
MP
, 
Somigliana
E
. Adenomyosis and reproductive performance after surgery for rectovaginal and colorectal endometriosis: a systematic review and meta‐analysis. Reprod Biomed Online. 2014;28:704–13.2474583110.1016/j.rbmo.2014.02.00611

Bianchi
PH
, 
Pereira
RM
, 
Zanatta
A
, 
Alegretti
JR
, 
Motta
EL
, 
Serafini
PC
. Extensive excision of deep infiltrative endometriosis before in vitro fertilization significantly improves pregnancy rates. J Minim Invasive Gynecol. 2009;16:174–80.1924970510.1016/j.jmig.2008.12.00912

Rubod
C
, 
Fouquet
A
, 
Bartolo
S
, 
Lepage
J
, 
Capelle
A
, 
Lefebvre
C
, et al. Factors associated with pregnancy after in vitro fertilization in infertile patients with posterior deep pelvic endometriosis: a retrospective study. J Gynecol Obstet Hum Reprod. 2019;48:235–9.2990895110.1016/j.jogoh.2018.06.00213

Capelle
A
, 
Lepage
J
, 
Langlois
C
, 
Lefebvre
C
, 
Dewailly
D
, 
Collinet
P
, et al. Surgery for deep infiltrating endometriosis before in vitro fertilization: no benefit for fertility?
Gynecol Obstet Fertil. 2015;43:109–16.2559594510.1016/j.gyobfe.2014.12.00314

Somigliana
E
, 
Garcia‐Velasco
JA
. Treatment of infertility associated with deep endometriosis: definition of therapeutic balances. Fertil Steril. 2015;104:764–70.2634224410.1016/j.fertnstert.2015.08.00315

Johnson
NP
, 
Hummelshoj
L
, World Endometriosis Society Montpellier Consortium
. Consensus on current management of endometriosis. Hum Reprod. 2013;28:1552–68.2352891610.1093/humrep/det05016
Japan Society of Gynecologic and Obstetric Endoscopy and Minimally Invasive Therapy
, editor. JSGOC guidelines for endoscopic surgery in obstetrics and gynecology 2019 [in Japanese]. Tokyo: KANEHARA & Co., LTD.; 2019. p. 53–7.10.4103/gmit.GMIT-D-25-00031PMC1233411940786668

## CQ 11 Is surgery effective in improving fertility in women with peritoneal lesions?

Surgical removal of peritoneal lesions improve the postoperative pregnancy rates.

Evidence level I

Strength of recommendation B

Numbers of studies referenced

Systematic Reviews 4

Cohort studies 10

Case–Control Studies 1

Commentary

In a meta‐analysis of three RCTs, which compared postoperative pregnancy rates between a group of patients who underwent surgical therapy for peritoneal lesions and a group of patients who underwent only diagnostic laparoscopy,^1^ live birth rate and the postoperative pregnancy rate were significantly higher in the surgery group (OR 1.89, 95% CI 1.25–2.86), while the clinical pregnancy rate in two RCTs was also significantly higher in the surgery group (OR 1.94, 95% CI 1.20–3.16). Several guidelines based on these results also recommend surgical therapy for infertile women with suspected r‐ASRM stage I/II endometriosis.^2^ Moreover, there is an opinion that laparoscopic examination for women with unexplained infertility should be used not only for diagnosis but also for women with suspected endometriosis with symptoms, such as pain.^2^ In a meta‐analysis, which included these two RCTs and four cohort studies, patients who underwent laparoscopic surgery demonstrated a significantly increased live birth rate (OR 1.52, 95% CI 1.26–1.84) and postoperative pregnancy rate (OR 1.44, 95% CI 1.24–1.68).^3^ In another study, the cumulative pregnancy rates at 12 months after surgical treatment for rAFS stages I and II endometriosis were 53.6% and 32.0%, respectively.^4^ In a cohort study that evaluated postoperative IVF/ICSI results in a group of 399 patients who underwent surgical removal of endometriosis and a group of 262 patients who underwent diagnostic laparoscopy only, the results of IVF/ICSI were all more favorable in the surgery group, which demonstrated a significantly higher implantation rate (30.9% vs 23.9%), pregnancy rate (40.1% vs. 29.4%), and live birth rate (27.7% vs 20.6%).^5^

The above studies demonstrate that the surgical removal of peritoneal lesions in infertile women may improve the postoperative spontaneous pregnancy rates and IVF outcomes. In addition, excision or ablation of peritoneal lesions discovered incidentally in fertility‐preserving surgery for conditions such as uterine fibroids can lead to improved fertility in the future. However, no studies have yet compared postoperative pregnancy rates in infertile women who underwent surgical therapy for peritoneal lesions versus those for whom fertility treatment was prioritized. Age, symptoms, and other aspects of the patient's background may be needed to be considered in these respective therapeutic options.

References1

Duffy
JM
, 
Arambage
K
, 
Correa
FJ
, et al. Laparoscopic surgery for endometriosis. Cochrane Database Syst Rev. 2014;4:CD011031.10.1002/14651858.CD011031.pub2246962652

Kho
RM
, 
Andres
MP
, 
Borrelli
GM
, 
Neto
JS
, 
Zanluchi
A
, 
Abrão
MS
. Surgical treatment of different types of endometrioses: comparison of major society guidelines and preferred clinical algorithms. Best Pract Res Clin Obstet Gynaecol. 2018;51:102–10.2954511410.1016/j.bpobgyn.2018.01.0203

Jin
X
, 
Ruiz Beguerie
JR
. Laparoscopic surgery for subfertility related to endometriosis: a meta‐analysis. Taiwan J Obstet Gynecol. 2014;53:303–8.2528678110.1016/j.tjog.2013.02.0044

Zeng
C
, 
Xu
JN
, 
Zhou
Y
, 
Zhou
YF
, 
Zhu
SN
, 
Xue
Q
. Reproductive performance after surgery for endometriosis: predictive value of the revised American Fertility Society classification and the endometriosis fertility index. Gynecol Obstet Invest. 2014;77:180–5.2460363210.1159/0003583905

Opøien
HK
, 
Fedorcsak
P
, 
Byholm
T
, 
Tanbo
T
. Complete surgical removal of minimal and mild endometriosis improves outcome of subsequent IVF/ICSI treatment. Reprod Biomed Online. 2011;23:389–95.2176438210.1016/j.rbmo.2011.06.002

## CQ 12 Is assisted reproductive technology (ART ) effective for endometriosis‐associated infertility?

Assisted reproductive technology (ART) for endometriosis‐associated infertility is as effective as that for other infertility factors; however, the initiation of ART must be properly timed.

Evidence level I

Strength of recommendation B

Numbers of studies referenced.

Systematic Reviews 8

Cohort studies 6

Case–Control Studies 5

Commentary

In Japan, the decline in the birth rate, and women marrying and having children at a later age in life are lifestyle changes that have made ART more common. ART is an evidence‐based treatment of unexplained infertility and intractable infertility, particularly in young women.^1^ Women with unexplained infertility are often diagnosed with endometriosis. ART yields the highest pregnancy rate per cycle and results in pregnancy in a shorter period than all other fertility treatments.

In 2014, Barbosa et al.^2^ reported the results of a large‐scale analysis of the effect of endometriosis on the outcomes of ART based on a systematic review of 92 studies and a meta‐analysis of 78 studies. Comparisons of ART outcomes between 20 167 women with endometriosis and 121 931 women without endometriosis showed no difference in clinical pregnancy rate (RR 0.95, 95% CI 0.89–1.02) or live birth rate (0.99, 95% CI 0.92–1.06); thus, the authors did not find that the presence of endometriosis had any effect. In a 2016 systematic review and meta‐analysis of 13 studies, Rossi and Prefumo^3^ compared 980 women with endometriosis with 5934 controls. The women with endometriosis demonstrated a lower clinical pregnancy rate (OR 0.65, 95% CI 0.44–0.96) but no change in the delivery rate (OR 1.17, 95% CI 0.69–1.98). These outcomes suggest that ART for infertility associated with endometriosis is as effective as ART for other infertility factors.

Regarding the effect of ovarian endometriotic cysts on ART outcomes, Hamdan et al.^4^ conducted a meta‐analysis of 33 studies in 2015. The presence of ovarian endometriotic cysts increased the cycle cancelation rate (OR 2.83, 95% CI 1.32–6.06) and reduced the number of retrieved but did not affect the clinical pregnancy rate of ART (OR 1.17, 95% CI 0.87–1.58) or live birth rate (OR 0.98, 95% CI 0.71–1.36). Regarding the reduction in the number of retrieved oocytes, Muzii et al.^5^ demonstrated in a 2018 meta‐analysis that AMH levels, an indicator of ovarian reserve, were reduced in the presence of ovarian endometriotic cysts.

In 2019, Somigliana et al.^6^ conducted a systematic review of 16 articles on the effect of ART on endometriosis. The review showed that ART does not exacerbate pain symptoms associated with endometriosis and does not increase the risk of endometriosis recurrence.

In a fertility treatment for women with endometriosis, the progression to ART is considered as per the patient's age, duration of infertility, presence of pain, treatment history, ovarian reserve, recurrence of endometriosis, and other infertility factors.^1^, ^7^ Due to the major effect of age on ART outcomes, the start of ART must be properly timed for infertile women aged ≥36 years.

References1
Japan Society for Reproductive Medicine
, editor. Essential knowledge in reproductive medicine 2020 [in Japanese]. Tokyo: Japan Society for Reproductive Medicine; 2020.2

Barbosa
MA
, 
Teixeira
DM
, 
Navarro
PA
, et al. Impact of endometriosis and its staging on assisted reproduction outcome: systematic review and meta‐analysis. Ultrasound Obstet Gynecol. 2014;44:261–78.2463908710.1002/uog.133663

Rossi
AC
, 
Prefumo
F
. The effects of surgery for endometriosis on pregnancy outcomes following in vitro fertilization and embryo transfer: a systematic review and meta‐analysis. Arch Gynecol Obstet. 2016;294:647–55.2730000210.1007/s00404-016-4136-44

Hamdan
M
, 
Dunselman
G
, 
Li
TC
, 
Cheong
Y
. The impact of endometrioma on IVF/ICSI outcomes: a systematic review and meta‐analysis. Hum Reprod Update. 2015;21:809–25.2616879910.1093/humupd/dmv0355

Muzii
L
, 
Di Tucci
C
, 
Di Feliciantonio
M
, et al. Antimüllerian hormone is reduced in the presence of ovarian endometriomas: a systematic review and meta‐analysis. Fertil Steril. 2018;110:932–940.e1.3031644010.1016/j.fertnstert.2018.06.0256

Somigliana
E
, 
Viganò
P
, 
Benaglia
L
, 
Busnelli
A
, 
Paffoni
A
, 
Vercellini
P
. Ovarian stimulation and endometriosis progression or recurrence: a systematic review. Reprod Biomed Online. 2019;38:185–94.3060997010.1016/j.rbmo.2018.11.0217

Dunselman
GA
, 
Vermeulen
N
, 
Becker
C
, 
Calhaz‐Jorge
C
, 
D'Hooghe
T
, 
De Bie
B
, et al. ESHRE guideline: management of women with endometriosis. Hum Reprod. 2014;29:400–12.2443577810.1093/humrep/det457

## CQ 13 Are hormone therapies effective for endometriosis‐associated infertility?

1. There is no evidence that hormone therapy is effective before general infertility treatment.

Evidence level I

Strength of recommendation D

2. Although there is scattered evidence that hormone therapy is effective prior to ART, more research is needed on optimal timing and agents.

Evidence level I

Strength of recommendation C

Numbers of studies referenced.

Systematic Reviews 2

RCTs 10

Case–Control Studies 2

Commentary

Studies evaluating hormone therapy for infertility associated with endometriosis are often divided into hormone therapy prior to general fertility treatment and hormone therapy prior to ART.

Regarding the effectiveness of hormone therapy for endometriosis before general infertility treatment, in three comparative studies (GnRH agonists versus antagonists, suppression of ovulation versus placebo, and presurgical medical therapy versus no treatment) reviewed in a 2014 Cochrane review,^1^ no significant difference in pregnancy rate was observed between these treatments. We identified four relevant RCTs, three of which (2 months of GnRH agonists versus aromatase inhibitors versus no treatment, one postoperative dose of GnRH agonist 3.75 mg versus no GnRH agonist, and 6 months of mifepristone versus Chinese herbal medicine) did not show any effect of hormone therapy. In a 2018 RCT that compared three postoperative treatments (6 months of GnRH agonists, 3 months of gestrinone and 3 months of mifepristone), the GnRH agonist group demonstrated a significantly increased pregnancy rate and a significantly reduced rate of complications during pregnancy; however, this RCT did not include a non‐treatment group. To summarize the above, there is no evidence that hormone therapy is effective for endometriosis prior to general infertility treatments, which is often viewed negatively; for example, the ESHRE guideline recommends against pretreatment hormone therapy because it delays the start of fertility treatment.

As for the effectiveness of hormone therapy prior to ART, in one study cited in the above Cochrane review,^1^ 3 months of GnRH agonists prior to ART yielded a higher pregnancy rate than no GnRH agonists (OR 4.28, 95% CI 2.00–9.15). Although this study is used as evidence to recommend medical therapy before ART in the ESHRE guidelines and elsewhere, the Cochrane review considers the quality of evidence in this study to be “very low.” We identified six relevant RCTs. In an RCT that examined the effect of 3 months of GnRH agonists on pregnancy rates, the treatment group did not show any change in the number of oocytes or pregnancy rate but did demonstrate a significantly low dose of FSH during controlled ovarian stimulation and a significantly shorter stimulation period.^2^ In an RCT, which compared 6 months of administration of dienogest (2 mg/day) and triptorelin (3.75 mg/month) to no treatment, the dienogest group had significantly higher pregnancy rate and delivery rate compared to the non‐treatment group (44.7% vs. 16.7% and 36.8% vs. 11.1%, respectively), while the triptorelin group did not have significant differences with the no‐treatment group.^3^ In addition to these studies, a Chinese group published a protocol for and began an RCT involving 1–2 months of GnRH agonists in 2018,^4^ while a Dutch group published a protocol for and began an RCT involving 6 months of OCs/LEPs or GnRH agonists in 2019^5^; reports of results from these RCTs are awaited. Although not a hormone preparation, an RCT reported that the PPAR‐γ agonist, pioglitazone, during the ovarian stimulation cycles resulted in significantly higher implantation rates than in patients who did not receive pioglitazone. In another RCT in which atosiban (an oxytocin receptor antagonist) was administered to patients with endometriosis prior to frozen–thawed embryo transfer, the treatment group demonstrated a significantly higher clinical pregnancy rate and implantation rate than the control group.^6^ In a case–control study, which examined the effect of the anti‐TNF‐α inhibitor etanercept (also not a hormone preparation), the administration of this agent 1 month before the ovarian stimulation cycle significantly increased the pregnancy rate. In a case–control study, Tamura et al. reported that GnRH agonists for 3 months before ART tended to increase implantation rates and pregnancy rates in the treatment group compared to those in the control group. As the authors discuss, these results are conceivably due to GnRH agonist therapy reducing cytotoxic cytokine secretion and oxidative stress in the ovary.^7^ Thus, medical therapy for endometriosis prior to ART could help improve pregnancy rates, if performed in a certain window of time. However, further research results are expected regarding the optimal timing and agents.

References1

Brown
J
, 
Farquhar
C
. Endometriosis: an overview of Cochrane reviews. Cochrane Database Syst Rev. 2014;3:CD009590.10.1002/14651858.CD009590.pub2PMC6984415246100502

Decleer
W
, 
Osmanagaoglu
K
, 
Verschueren
K
, 
Comhaire
F
, 
Devroey
P
. RCT to evaluate the influence of adjuvant medical treatment of peritoneal endometriosis on the outcome of IVF. Hum Reprod. 2016;31:2017–23.2737035910.1093/humrep/dew1483

Muller
V
, 
Kogan
I
, 
Yarmolinskaya
M
, 
Niauri
D
, 
Gzgzyan
A
, 
Aylamazyan
E
. Dienogest treatment after ovarian endometrioma removal in infertile women prior to IVF. Gynecol Endocrinol. 2017;33(Suppl 1):18–21.2926498510.1080/09513590.2017.14156764

Kong
H
, 
Hu
L
, 
Nie
L
, 
Yu
X
, 
Dai
W
, 
Li
J
, et al. A multi‐center randomized controlled clinical trial of the application of a shortened protocol of long‐acting Triptorelin down‐regulated prior to IVF/ICSI among patients with endometriosis: a protocol. Reprod Health. 2018;15:213.3057291610.1186/s12978-018-0639-8PMC63024815

van der Houwen
LEE
, 
Lier
MCI
, 
Schreurs
AMF
, 
van Wely
M
, 
Hompes
PGA
, 
Cantineau
AEP
, et al. Continuous oral contraceptives versus long‐term pituitary desensitization prior to IVF/ICSI in moderate to severe endometriosis: study protocol of a non‐inferiority randomized controlled trial. Hum Reprod Open. 2019;2019:hoz001.3089526610.1093/hropen/hoz001PMC63966446

He
Y
, 
Wu
H
, 
He
X
, 
Xing
Q
, 
Zhou
P
, 
Cao
Y
, et al. Administration of atosiban in patients with endometriosis undergoing frozen‐thawed embryo transfer: a prospective, randomized study. Fertil Steril. 2016;106:416–22.2714351810.1016/j.fertnstert.2016.04.0197

Tamura
H
, 
Takasaki
A
, 
Nakamura
Y
, 
Numa
F
, 
Sugino
N
. A pilot study to search possible mechanisms of ultralong gonadotropin‐releasing hormone agonist therapy in IVF‐ET patients with endometriosis. J Ovarian Res. 2014;7:100.2533106610.1186/s13048-014-0100-8PMC4207622

## CQ 14 Is surgery effective for endometriosis‐associated pain (excluding deep lesions)?

Excision of peritoneal lesions and removal of ovarian endometriotic cysts reduces endometriosis‐associated pain.

Evidence level I

Strength of recommendation B

Numbers of studies referenced.

Systematic Reviews 3

Cohort studies 6

Commentary

Although hormone therapy is generally effective for endometriosis‐associated pain, it is ineffective in approximately 30% of patients.^1^ For these patients who do not respond to medical therapy, surgical therapy is considered. In a meta‐analysis, which evaluated pain over 6 months in three RCTs, which divided patients with r‐ASRM stage I/II endometriosis into a group that underwent laparoscopic lesion excision and a group that underwent only diagnostic laparoscopy, pain improved significantly in the lesion excision group (OR 6.58, 95% CI 3.31–13.10). In addition, a Cochrane review also found that pain similarly improved in studies that examined pain over 12 months (OR 10.0, 95% CI 3.21–31.17), thus demonstrating that the surgical removal of endometriotic foci is effective for improving pain in r‐ASRM stage I/II endometriosis.^2^ Although one study found that excision and ablation of peritoneal lesions are equally effective in reducing pain,^3^ another study has reported that patients who underwent excision experienced greater reduction in chronic pelvic pain and dyspareunia.^4^ However, in another study, lesions recurred in 37% of cases of excision or ablation of peritoneal lesions, possibly due to incomplete excision of the foci.^5^

In a meta‐analysis, which compared laparoscopic excision of ovarian endometriotic cysts with ablation, the excision group demonstrated significantly lower frequencies of dysmenorrhea (OR 0.15, 95% CI 0.06–0.38), dyspareunia (OR 0.08, 95% CI 0.01–0.51), chronic pelvic pain (OR 0.10, 95% CI 0.02–0.56), recurrence of ovarian endometriotic cysts (OR 0.41, 95% CI 0.18–0.93), and reoperation (OR 0.21, 95% CI 0.05–0.79),^6^ suggesting that excision is a more effective operative procedure for ovarian endometriotic cysts. However, caution is required regarding the recurrence of pain following surgical therapy for ovarian endometriotic cysts.

References1

Budden
A
, 
Ravendran
K
, 
Abbott
JA
. Identifying the problems of randomized controlled trials for the surgical management of endometriosis‐associated pelvic pain. J Minim Invasive Gynecol. 2020;27:419–32.3171216110.1016/j.jmig.2019.11.0022

Duffy
JM
, 
Arambage
K
, 
Correa
FJ
, 
Olive
D
, 
Farquhar
C
, 
Garry
R
, et al. Laparoscopic surgery for endometriosis. Cochrane Database Syst Rev. 2014;4:CD011031.10.1002/14651858.CD011031.pub2246962653

Riley
KA
, 
Benton
AS
, 
Deimling
TA
, 
Kunselman
AR
, 
Harkins
GJ
. Surgical excision versus ablation for superficial endometriosis‐associated pain: a randomized controlled trial. J Minim Invasive Gynecol. 2019;26:71–7.2960903210.1016/j.jmig.2018.03.0234

Pundir
J
, 
Omanwa
K
, 
Kovoor
E
, 
Pundir
V
, 
Lancaster
G
, 
Barton‐Smith
P
. Laparoscopic excision versus ablation for endometriosis‐associated pain: an updated systematic review and meta‐analysis. J Minim Invasive Gynecol. 2017;24:747–56.2845661710.1016/j.jmig.2017.04.0085

Taylor
E
, 
Williams
C
. Surgical treatment of endometriosis: location and patterns of disease at reoperation. Fertil Steril. 2010;93:57–61.1900679210.1016/j.fertnstert.2008.09.0856

Hart
RJ
, 
Hickey
M
, 
Maouris
P
, 
Buckett
W
, Cochrane Gynaecology and Fertility Group
. Excisional surgery versus ablative surgery for ovarian endometriomata. Cochrane Database Syst Rev. 2008;2:CD004992.10.1002/14651858.CD004992.pub318425908

## CQ 15 Is surgery effective for deep rectovaginal endometriosis‐associated pain?

Surgical therapy reduces pain but may require attention to complications.

Evidence level III

Strength of recommendation C

Numbers of studies referenced.

Systematic Reviews 6

Cohort studies 4

Case–Control Studies 14

Practice Guidelines 3.

Commentary

Deep endometriosis is considered as adenomyosis externa observed in the rectum, sigmoid colon, rectovaginal pouch, uterosacral ligaments, rectovaginal septum and vesicouterine pouch and is observed in 1%–2% of cases of endometriosis.^1^, ^2^, ^3^ Deep endometriosis results in chronic pelvic pain symptoms, such as severe dysmenorrhea, dyspareunia, and dyschezia, and infertility.^4^ In some cases, deep endometriosis also triggers hydronephrosis and intestinal obstruction associated with stenosis of the ureter and rectum.

Although pain associated with deep endometriosis can be alleviated with medical therapy,^5^ the lesions themselves do not disappear.^2^ Surgical therapy is indicated for cases in which medical therapy does not adequately improve symptoms and cases in which hormone therapy cannot be performed due to the patient's desire to have children.^5^

Surgical therapy is highly effective for deep endometriosis. The opening of the rectovaginal pouch and the excision of deep lesions in 114 patients with deep rectovaginal endometriosis significantly reduced VAS scores for dysmenorrhea compared to baseline, while the percentages of patients with dyspareunia and dyschezia before surgery (52.6% and 42.4%, respectively) decreased to 4.6% and 4.9%, respectively, after surgery.^6^ According to a systematic review of 23 studies on deep endometriosis excision, surgery markedly improved patient QOL and pain and is considered the first‐line option, especially for patients with severe pain (VAS >7).^7^

Deep lesions expand laterally and dorsally around the uterosacral ligaments, meaning that the resection of deep lesions may injure the ureter and rectum. Furthermore, injury to the hypogastric nerve and pelvic plexus, which are lateral to the uterosacral ligaments, triggers dysuria. Therefore, ensuring sufficient surgical space through means such as confirming the course of the ureter based on a systematic approach and opening of the pararectal space, and identifying and preserving the hypogastric nerve and pelvic plexus in advance are important for removing the foci safely and thoroughly.^8^ Several studies have reported nerve‐sparing surgery for deep endometriosis. A meta‐analysis of four studies found a significant improvement for nerve‐sparing surgery compared to non‐nerve‐sparing surgery with a RR of 0.19 for the need for self‐catheterization at discharge and 0.16 for persistent urinary retention 90 days postoperatively.^9^ However, even with nerve‐sparing surgery, resection of deep lesions in the uterosacral ligaments results in incomplete transient emptying and other urinary complications in approximately 30% of cases, with resection of bilateral uterosacral ligament lesions considered to involve particularly elevated risks.^10^

Complications have been reported to occur in 1.5%–3.4% of cases after surgical removal of ovarian endometriotic ovarian cysts without rectal surgery.^11^ Kondo et al. reported that in 568 cases of deep endometriosis surgery, postoperative complications occurred in 9.3% of cases with rectal surgery and in 1.5% of cases without rectal surgery.^11^ In a study that included 130 patients with deep endometriosis without involvement of the digestive or urinary tracts, Vallée et al. divided patients into groups based on the size of the nodule size (<1 cm, 1–3 cm, > 3 cm) and reported that postoperative complications occurred in 0.8%, 4.6% and 5.4% of the patients in these respective groups, without severe cases. The authors concluded that surgical treatment is recommended when medical treatment does not produce sufficient results.^12^

The above studies suggest that laparoscopic surgery is effective for pain associated with deep rectovaginal endometriosis. However, serious complications, albeit relatively infrequent, do occur. We recommend that laparoscopic surgery be performed by an experienced physician in a facility, which allows multidisciplinary team coordination with other departments.^13^

References1

Chapron
C
, 
Fauconnier
A
, 
Vieira
M
, 
Barakat
H
, 
Dousset
B
, 
Pansini
V
, et al. Anatomical distribution of deeply infiltrating endometriosis: surgical implications and proposition for a classification. Hum Reprod. 2003;18:157–61.1252545910.1093/humrep/deg0092

Koninckx
PR
, 
Oosterlynck
D
, 
D'Hooghe
T
, et al. Deeply infiltrating endometriosis is a disease whereas mild endometriosis could be considered a non‐disease. Ann N Y Acad Sci. 1994;734:333–41.797893510.1111/j.1749-6632.1994.tb21763.x3

Koninckx
PR
, 
Ussia
A
, 
Adamyan
L
, 
Wattiez
A
, 
Donnez
J
. Deep endometriosis: definition, diagnosis, and treatment. Fertil Steril. 2012;98:564–71.2293876910.1016/j.fertnstert.2012.07.10614

Fauconnier
A
, 
Chapron
C
, 
Dubuisson
JB
, 
Vieira
M
, 
Dousset
B
, 
Bréart
G
. Relation between pain symptoms and the anatomic location of deep infiltrating endometriosis. Fertil Steril. 2002;78:719–26.1237244610.1016/s0015-0282(02)03331-95

Vercellini
P
, 
Buggio
L
, 
Somigliana
E
. Role of medical therapy in the management of deep rectovaginal endometriosis. Fertil Steril. 2017;108:913–30.2920296510.1016/j.fertnstert.2017.08.0386

Takeuchi
H
. Pathology of complete cul de sac obliteration in endometriosis and the treatment strategy based on laparoscopic operation [in Japanese]. Acta Obstet Gynaecol Jpn. 2003;55:903–14.7

de Paula
AM
, 
Borrelli
GM
, 
Kho
RM
, et al. The current management of deep endometriosis: a systematic review. Minerva Ginecol. 2017;69:587–96.2854529310.23736/S0026-4784.17.04082-58

Kavallaris
A
, 
Banz
C
, 
Chalvatzas
N
, 
Hornemann
A
, 
Luedders
D
, 
Diedrich
K
, et al. Laparoscopic nerve‐sparing surgery of deep infiltrating endometriosis: description of the technique and patients' outcome. Arch Gynecol Obstet. 2011;284:131–5.2068030910.1007/s00404-010-1624-99

de Resende
JA
Júnior
, 
Cavalini
LT
, 
Crispi
CP
, et al. Risk of urinary retention after nerve‐sparing surgery for deep infiltrating endometriosis: a systematic review and meta‐analysis. NeurourolUrodyn. 2017;36:57–61.10.1002/nau.229152647915810

Dubernard
G
, 
Rouzier
R
, 
David‐Montefiore
E
, 
Bazot
M
, 
Daraï
E
. Urinary complications after surgery for posterior deep infiltrating endometriosis are related to the extent of dissection and to uterosacral ligaments resection. J Minim Invasive Gynecol. 2008;15:235–40.1831300010.1016/j.jmig.2007.10.00911

Kondo
W
, 
Bourdel
N
, 
Tamburro
S
, 
Cavoli
D
, 
Jardon
K
, 
Rabischong
B
, et al. Complications after surgery for deeply infiltrating pelvic endometriosis. BJOG. 2011;118:292–8.2108386310.1111/j.1471-0528.2010.02774.x12

Vallée
A
, 
Ploteau
A
, 
Abo
C
, et al. Surgery for deep endometriosis without involvement of digestive or urinary tracts: do not worry the patients!
Fertil Steril. 2018;109:1079–1085.e1.2993564410.1016/j.fertnstert.2018.02.12413
Japan Society of Gynecologic and Obstetric Endoscopy and Minimally Invasive Therapy
, editor. JSGOC guidelines for endoscopic surgery in obstetrics and gynecology 2019 [in Japanese]. Tokyo: KANEHARA & Co., LTD.; 2019. p. 53–7.10.4103/gmit.GMIT-D-25-00031PMC1233411940786668

## CQ 16 Are oral contraceptive/low dose estrogen‐progestin (OCs /LEPs ) effective for endometriosis‐associated pain?

OCs/LEPs are effective for reducing endometriosis‐associated pain.

Evidence level I

Strength of recommendation B

Numbers of studies referenced

Systematic Reviews 3

RCTs 9

Practice Guidelines 2

Commentary

Endometriosis‐associated pain is broadly divided into types, such as menstrual pain, chronic pelvic pain, dyschezia, and dyspareunia. We selected three relevant systematic reviews,^1^, ^2^, ^3^ all of which concluded that OCs/LEPs are effective for menstrual pain, pelvic pain, and dyspareunia associated with endometriosis.

In a Cochrane review on OCs/LEPs and endometriosis‐associated pain,^1^ five RCTs met the selection criteria, and three of these RCTs^4^, ^5^, ^6^ were ultimately selected and analyzed.

Two of these RCTs were double‐blind trials comparing OCs/LEPs with a placebo.^5^, ^6^ In the first ever placebo‐controlled trial, conducted in Japan, an OC/LEP (ethinylestradiol 0.035 mg + norethisterone 1 mg) significantly reduced the VAS for menstrual pain compared to placebo.^5^ In addition, a flexible regimen of ethinylestradiol 0.020 mg + drospirenone 3 mg for 120 consecutive days with a tablet‐free interval in the event of ≥3 consecutive days of irregular vaginal bleeding significantly improved menstrual pain, dyspareunia, and dyschezia compared to a placebo group.^1^, ^6^

In a double‐blind RCT of a continuous OC/LEP and GnRH agonist + add‐back therapy, both treatments were equally effective for menstrual pain, pelvic pain, and dyspareunia.^7^

There is no clear evidence on whether cyclic or a continuous OC/LEP combination is more effective for pain associated with endometriosis. However, in an RCT, which compared a cyclic OC/LEP (ethinylestradiol 0.02 mg + levonorgestrel 0.09 mg), an extended 84‐day regimen of OC/LEP, and a placebo for patients with dysmenorrhea, the extended regimen group demonstrated significantly reduced pain compared to the cyclic regimen group.^8^ Approximately 70% of subjects had endometriosis, suggesting that an extended regimen of OC/LEP is also more effective for endometriosis‐associated pain.

References1

Brown
J
, 
Crawford
TJ
, 
Datta
S
, 
Prentice
A
, Cochrane Gynaecology and Fertility Group
. Oral contraceptives for pain associated with endometriosis. Cochrane Database Syst Rev. 2018;5:CD001019.2978682810.1002/14651858.CD001019.pub3PMC64946342

Jensen
JT
, 
Schlaff
W
, 
Gordon
K
. Use of combined hormonal contraceptives for the treatment of endometriosis‐related pain: a systematic review of the evidence. Fertil Steril. 2018;110:137–152.e1.2993715210.1016/j.fertnstert.2018.03.0123

Grandi
G
, 
Barra
F
, 
Ferrero
S
, 
Sileo
FG
, 
Bertucci
E
, 
Napolitano
A
, et al. Hormonal contraception in women with endometriosis: a systematic review. Eur J Contracept Reprod Health Care. 2019;24:61–70.3066438310.1080/13625187.2018.15505764

Vercellini
P
, 
Trespidi
L
, 
Colombo
A
, 
Vendola
N
, 
Marchini
M
, 
Crosignani
PG
. A gonadotropin‐releasing hormone agonist versus a low‐dose oral contraceptive for pelvic pain associated with endometriosis. Fertil Steril. 1993;60:75–9.85139625

Harada
T
, 
Momoeda
M
, 
Taketani
Y
, 
Hoshiai
H
, 
Terakawa
N
. Low‐dose oral contraceptive pill for dysmenorrhea associated with endometriosis: a placebo‐controlled, double‐blind, randomized trial. Fertil Steril. 2008;90:1583–8.1816400110.1016/j.fertnstert.2007.08.0516

Harada
T
, 
Kosaka
S
, 
Elliesen
J
, 
Yasuda
M
, 
Ito
M
, 
Momoeda
M
. Ethinylestradiol 20 μg/drospirenone 3 mg in a flexible extended regimen for the management of endometriosis‐associated pelvic pain: a randomized controlled trial. Fertil Steril. 2017;108:798–805.2891192510.1016/j.fertnstert.2017.07.11657

Guzick
DS
, 
Huang
LS
, 
Broadman
BA
, 
Nealon
M
, 
Hornstein
MD
. Randomized trial of leuprolide versus continuous oral contraceptives in the treatment of endometriosis‐associated pelvic pain. Fertil Steril. 2011;95:1568–73.2130033910.1016/j.fertnstert.2011.01.027PMC42717948

Harada
T
, 
Momoeda
M
. Efficacy of cyclic and extended regimens of ethinylestradiol 0.02mg‐ levonorgestrel 0.09mg for dysmenorrhea: a placebo‐controlled, double‐blind, randomized trial. Reprod Med Biol. 2021;20:215–23.3385045510.1002/rmb2.12373PMC8022088

## CQ 17 Are GnRH agonists effective for endometriosis‐associated pain?

GnRH agonists are effective for reducing endometriosis‐associated pain.

Evidence level I

Strength of recommendation B

Numbers of studies referenced

Systematic Reviews 10

RCTs 11

Practice Guidelines 1

Commentary

In multiple RCTs, GnRH agonists have demonstrated therapeutic effects equal to danazol, OCs/LEPs, dienogest, and LNG‐IUS (Mirena®). Due to the characteristic differences in clinical and adverse effects among the above, their use should be tailored to individual patients based on an understanding of their respective benefits and disadvantages and factors, such as the patient's age, the additional performance of surgical therapy, and whether the patient wants to have children.

Several RCTs have examined the therapeutic effects of GnRH agonists for pain associated with endometriosis. In a 2010 systematic review by Brown et al., an analysis showed that GnRH agonists have a greater therapeutic effect on pain than no treatment or a placebo.^1^ In an RCT, which examined GnRH agonist doses, different doses did not produce differences in therapeutic effects. In an RCT that examined the duration of the GnRH agonist (3 vs. 6 months), the only improvement was dyspareunia at 3 months of therapy. There was also no difference in therapeutic effects between intranasal administration and injections.

In Japan, GnRH agonists have been used to treat endometriosis since 1994. Currently, GnRH agonists are used intranasally and as injections; for injections, there are high‐dose and low‐dose formulations. Each formulation differs in terms of adverse effects and frequency of administration.

Add‐back therapy, which combines estrogen preparations and progestin preparations, is used to alleviate adverse drug reactions and increase the therapeutic effects of GnRH agonists.^2^, ^3^ However, this add‐back therapy is not covered by medical insurance in Japan. Add‐back therapy is considered not to affect the therapeutic effects of pain. Switching to another agent after GnRH agonist administration^4^ has also been considered; the construction of evidence based on future RCTs is anticipated. The criteria for selecting patients eligible for GnRH agonists must be developed according to factors, such as the additional performance of surgical treatment, the patient's age, and whether the patient wishes to have children in the future.

References1

Brown
J
, 
Pan
A
, 
Hart
RJ
. Gonadotrophin‐releasing hormone analogues for pain associated with endometriosis. Cochrane Database Syst Rev. 2010;12:CD008475.10.1002/14651858.CD008475.pub2PMC7388859211543982

Wu
D
, 
Hu
M
, 
Hong
L
, 
Hong
S
, 
Ding
W
, 
Min
J
, et al. Clinical efficacy of add‐back therapy in treatment of endometriosis: a meta‐analysis. Arch Gynecol Obstet. 2014;290:513–23.2472814510.1007/s00404-014-3230-83

Granese
R
, 
Perino
A
, 
Calagna
G
, 
Saitta
S
, 
de Franciscis
P
, 
Colacurci
N
, et al. Gonadotrophin‐releasing hormone analogue or dienogest plus estradiol valerate to prevent pain recurrence after laparoscopic surgery for endometriosis: a multi‐center randomized trial. Acta Obstet Gynecol Scand. 2015;94:637–45.2576158710.1111/aogs.126334

Kitawaki
J
, 
Kusuki
I
, 
Yamanaka
K
, 
Suganuma
I
. Maintenance therapy with dienogest following gonadotropin‐releasing hormone agonist treatment for endometriosis‐associated pelvic pain. Eur J Obstet Gynecol Reprod Biol. 2011;157:212–6.2147423210.1016/j.ejogrb.2011.03.012

## CQ 18 Are oral progestins effective for endometriosis‐associated pain?

Dienogest is effective for reducing endometriosis‐associated pain.

Evidence level I

Strength of recommendation B

Numbers of studies referenced.

Systematic Reviews 13

RCTs 11

Case–Control Studies 34

Commentary

Progestins are known to trigger endometrial decidualization and cause endometrial atrophy. Consequently, they have long been known to be effective for endometriosis. However, they also trigger irregular vaginal bleeding as an adverse effect and are therefore often combined with estrogen preparations. As a result, the use of progestins alone has become uncommon. Furthermore, due to the low level of progesterone receptor expression in endometriotic lesions, it is now considered necessary to take high doses of progestin to achieve a therapeutic effect.

A 2021 systematic review identified eight RCTs, which involved the use of progestins alone^1^; however, only two of these RCTs were placebo‐controlled. In 1994, Overton et al. examined the effects of 6 months of two high doses of dydrogesterone (40 or 60 mg/day from days 16 to 27 of the menstruation cycle) on post‐treatment endometriotic lesion scores and pain for 62 patients with endometriosis (mild) who had pain symptoms or wished to have children compared to a placebo group. Consequently, a dose of 60 mg/day of dydrogesterone was found to reduce pain.^2^ However, although this study is an RCT, its evidence level is not high; for example, lesions were assessed in only 39 of the 62 patients enrolled in the study. Since then, two other clinical trials, although single arm trials, have been conducted regarding dydrogesterone alone for endometriosis and dysmenorrhea,^3^, ^4^ both of which found dydrogesterone to reduce pain. Going forward, meta‐analyzes and RCTs will be necessary to verify the efficacy of dydrogesterone in reducing endometriosis‐associated pain.

Dienogest is an oral progestin, which was made commercially available in Japan in January 2008, earlier than any other country worldwide. It is characterized by its high selectivity for progesterone receptors and its absence of androgen effects. Additionally, dienogest is known to inhibit ovulation and ovarian function and reduce endometriotic foci by acting directly on endometriotic tissue. Several RCTs have examined the therapeutic effect of dienogest on endometriosis‐associated pain.^5^ One of these RCTs was placebo‐controlled. This trial, which was conducted in Europe, examined the effects of dienogest (2 mg/day for 12 weeks) on endometriosis‐associated pain in 198 patients with endometriosis (stages I − IV). Although dienogest improved pain scores, the following adverse effects were observed: prolonged irregular vaginal bleeding (24.5% in the dienogest group versus 5.4% in the placebo group), headache (11% vs. 5%), and cystitis (3% vs. 0%).^6^ However, a Japanese RCT that predated the above‐cited European trial obtained different results. This trial, which was conducted with 271 patients with endometriosis, examined the effects of 24 weeks of dienogest (2 mg/day), with buserelin (900 μg/day) administered to controls. The pain improvement effect and the change in QOL obtained at 24 weeks of dienogest were similar to those obtained with buserelin.^7^ A follow‐up study to the above‐mentioned European RCT and a trial examining long‐term treatment (dienogest 2 mg/day for 52 weeks) also found that dienogest significantly reduced scores for lower abdominal pain and low back pain.^7^, ^8^ Furthermore, another study has reported that the number of days of metrorrhagia gradually decreases as the dienogest treatment period progresses, while the bone density reduction rate achieved only −1.7% at 52 weeks.^9^ Thus, although dienogest is associated with frequent metrorrhagia, it reduces pain and has been shown to be safe in the long term.

To date, no RCT has examined the effectiveness of progestins in endometriosis at less common sites. In a retrospective study, dienogest for rectosigmoid endometriosis improved pain symptoms and reduced the size of lesions.^10^

References1

Brown
J
, 
Kives
S
, 
Akhtar
M
. Progestagens and anti‐progestagens for pain associated with endometriosis. Cochrane Database Syst Rev. 2012;3:CD002122.10.1002/14651858.CD002122.pub2PMC6885053224192842

Overton
CE
, 
Lindsay
PC
, 
Johal
B
. A randomized, double‐blind, placebo‐controlled study of luteal phase dydrogesterone in women with minimal to mild endometriosis. Fertil Steril. 1994;62:701–7.792607610.1016/s0015-0282(16)56991-x3

Trivedi
P
, 
Selvaraj
K
, 
Mahapatra
PD
, 
Srivastava
S
, 
Malik
S
. Effective post‐laparoscopic treatment of endometriosis with dydrogesterone. Gynecol Endocrinol. 2007;23(Suppl 1):73–6.1794354310.1080/095135907016695834

Taniguchi
F
, 
Ota
I
, 
Iba
Y
, 
Toda
T
, 
Tagashira
Y
, 
Ohata
Y
, et al. The efficacy and safety of dydrogesterone for treatment of dysmenorrhea: an open‐label multicenter clinical study. J Obstet Gynaecol Res. 2019;45:168–75.3024627610.1111/jog.138075

Ferrero
S
, 
Remorgida
V
, 
Venturini
PL
, 
Bizzarri
N
. Endometriosis: the effect of dienogest. BMJ Clin Evid. 2015;2015:0802.PMC4461025260571016

Strowizki
T
, 
Faustmann
T
, 
Gerlinger
C
, 
Seitz
C
. Dienogest in the treatment of endometriosis‐associated pelvic pain: a 12 week, randomized, double‐blind, placebo‐controlled study. Eur J Obstet Gynecol Reprod Biol. 2010;151:193–8.2044453410.1016/j.ejogrb.2010.04.0027

Harada
T
, 
Momoeda
M
, 
Taketani
Y
, 
Aso
T
, 
Fukunaga
M
, 
Hagino
H
, et al. Dienogest is as effective as intranasal buserelin acetate for the relief of pain symptoms associated with endometriosis‐ a randomized, double, multi‐Centre trial. Fertil Steril. 2009;91:675–81.1865318410.1016/j.fertnstert.2007.12.0808

Petraglia
F
, 
Hornung
D
, 
Seitz
C
, 
Faustmann
T
, 
Gerlinger
C
, 
Luisi
S
, et al. Reduced pelvic pain in women with endometriosis: efficacy of long‐term dienogest treatment. Arch Gynecol Obstet. 2012;285:167–73.2168151610.1007/s00404-011-1941-7PMC32492039

Momoeda
M
, 
Harada
T
, 
Terakawa
N
, 
Aso
T
, 
Fukunaga
M
, 
Hagino
H
, et al. Long‐term use of dienogest for the treatment of endometriosis. J Obstet Gynaecol Res. 2009;35:1069–76.2002563310.1111/j.1447-0756.2009.01076.x10

Harada
M
, 
Osuga
Y
, 
Izumi
G
, 
Takamura
M
, 
Takemura
Y
, 
Hirata
T
, et al. Dienogest, a new conservative strategy for extragenital endometriosis: a pilot study. Gynecol Endocrinol. 2011;27:717–20.2115849410.3109/09513590.2010.533800

## CQ 19 Is the levonorgestrel intrauterine system (LNG‐IUS ) effective for endometriosis‐associated pain?

LNG‐IUS is effective for reducing endometriosis‐associated pain.

Evidence level I

Strength of recommendation B

Numbers of studies referenced.

Systematic Reviews, Meta‐analyses 1.

RCTs 2.

Commentary

In a study that included 12 months of LNG‐IUS (Mirena®) for 45 patients with endometriosis, the patients showed reductions in dyspareunia, dysmenorrhea symptoms, the size of the endometriotic cyst of the ovary, and CA 125 levels. In particular, the discontinuation rate (6.7%) was low.^1^ In a prospective study evaluating the efficacy of LNG‐IUS for patients with treatment‐resistant endometriosis, improvement in pain symptoms was observed in nearly 50% of patients at 3 months, 60% of patients at 6 months, and almost 70% of patients at the end of the follow‐up at 22 months. The above results demonstrate that LNG‐IUS should be considered for treatment‐resistant endometriosis before radical surgery is performed.^2^ Although the results of a retrospective cohort study suggest that LNG‐IUS insertion is an option for adolescents and young women (age range: 14–22 years), a prospective study is currently needed.^3^ According to a prospective study of 28 women with mild endometriosis, the mechanism by which LNG‐IUS reduces pain involves a reduction in mast cell numbers in eutopic and ectopic endometrium.^4^

A meta‐analysis of five RCTs found that both LNG‐IUS and GnRH agonists reduced VAS pain scores, reduced CA125 levels, reduced r‐ASRM scores, and increased well‐being scores, with no differences between the two treatments. On LNG‐IUS further reduced other hand, the low‐density lipoprotein (LDL) cholesterol levels. Based on the above, while LNG‐IUS and GnRH agonists have equivalent clinical efficacy, the greater reduction in LDL cholesterol levels with LNG‐IUS suggests that it has clinical advantages over GnRH agonists.^5^

References1

Yucel
N
, 
Baskent
E
, 
Karamustafaoglu Balci
B
, 
Goynumer
G
. The levonorgestrel‐releasing intrauterine system is associated with a reduction in dysmenorrhoea and dyspareunia, a decrease in CA 125 levels, and an increase in quality of life in women with suspected endometriosis. Aust N Z J Obstet Gynaecol. 2018;58:560–3.2935945710.1111/ajo.127732

Matorras
R
, 
Ballesteros
A
, 
Prieto
B
, 
Ocerin
I
, 
Expósito
A
, 
Pijoan
JI
, et al. Efficacy of the levonorgestrel‐releasing intrauterine device in the treatment of recurrent pelvic pain in multitreated endometriosis. J Reprod Med. 2011;56:497–503.221953333

Yoost
J
, 
LaJoie
AS
, 
Hertweck
P
, 
Loveless
M
. Use of the levonorgestrel intrauterine system in adolescents with endometriosis. J Pediatr Adolesc Gynecol. 2013;26:120–4.2351819010.1016/j.jpag.2012.11.0024

Engemise
SL
, 
Willets
JM
, 
Emembolu
JO
, 
Konje
JC
. The effect of the levonorgestrel‐releasing intrauterine system, Mirena® on mast cell numbers in women with endometriosis undergoing symptomatic treatment. Eur J Obstet Gynecol Reprod Biol. 2011;159:439–42.2201883110.1016/j.ejogrb.2011.09.0075

Lan
S
, 
Ling
L
, 
Jianhong
Z
, 
Xijing
J
, 
Lihui
W
. Analysis of the levonorgestrel‐releasing intrauterine system in women with endometriosis. J Int Med Res. 2013;41:548–58.2366008710.1177/0300060513479865

## CQ 20 Is there evidence of a superior of efficacy between oral contraceptive/low dose estrogen‐progestin (OCs /LEPs ), GnRH agonists, and progestins for endometriosis‐associated pain?

OC/LEPs, GnRH agonists, dienogest, and LNG‐IUS all have equivalent pain reduction effects.

Evidence level I

Strength of recommendation B

Numbers of studies referenced

Systematic Reviews 7

RCTs 15

Case–Control Studies 9

Commentary.

Medical therapy for endometriosis‐associated pain includes not only conventional danazol, OCs/LEPs, and GnRH agonists, but also the more recently commercially available oral progestin dienogest and LNG‐IUS (Mirena®), which is designed to release progestins in the uterine. If these agents are used accurately with an understanding of their respective characteristics according to the condition and status of an individual patient, they can be expected to further improve the QOL of patients with endometriosis. The following are RCTs that compared the above agents.

1. OCs/LEPs vs. GnRH agonists.^1^

In a trial that compared the efficacy between goserelin (3.6 mg every 4 weeks) and OC/LEP (ethinylestradiol 0.02 mg/day + desogestrel 0.15 mg/day) treatments at 6 months in 57 endometriosis patients, both groups demonstrated a significant improvement in pelvic pain and dyspareunia.^2^ An RCT (*n* = 47) that compared the efficacy between leuprolide (11.25 mg every 12 weeks) with add‐back using norethindrone acetate (5 mg/day) versus OC/LEP treatments (ethinylestradiol 0.035 mg/day + norethindrone 1 mg/day) at 48 weeks also found equivalent pain reduction effects between the two treatments.^3^

2. GnRH agonists vs. progestins.

(1) GnRH agonists vs. oral progestins.^4^

Two relevant RCTs have been reported. An RCT conducted in Japan compared the efficacies of dienogest (2 mg/day) versus buserelin (900 μg/day) at 24 weeks in 271 patients with endometriosis. The trial found that the pain reduction effect and the change in QOL associated with dienogest were equivalent to those associated with buserelin.^5^ A European RCT, albeit an open‐label trial, compared dienogest (2 mg/day) and leuprolide (3.75 every 4 weeks). When the pain reduction effects of the respective agents were compared in 252 patients with endometriosis at 24 weeks, the two agents demonstrated equivalent efficacy. The results of adverse drug reactions in this RCT were similar to those of the Japanese RCT.^6^

(2) GnRH agonists vs. LNG‐IUS^7^

A relevant meta‐analysis identified five RCTs. Of these, four RCTs assessed endometriosis‐associated pain and used leuprolide or goserelin as a GnRH agonist. In all of these RCTs, LNG‐IUS demonstrated a pain‐reduction effect equivalent to that of GnRH agonists.

3. OCs/LEPs vs. progestins^4^

A placebo‐controlled RCT (with the actual drug administered from 24 weeks onwards) for an OC/LEP (ethinylestradiol 0.020 mg + drospirenone 3 mg in a flexible regimen lasting 120 consecutive days or with a tablet‐free interval in the event of ≥3 consecutive days of bleeding) was conducted in Japan. In this RCT, a dienogest arm (2 mg/day for 52 weeks) was established as a control arm.^8^ A comparison (albeit not randomized) between the flexible LEP regimen and dienogest demonstrated equivalent pain reduction effects.

An Italian group reported an RCT comparing progestin cyproterone acetate (12.5 mg/day) with an OC/LEP (ethinylestradiol 0.02 mg/day + desogestrel 0.15 mg/day); both treatments produced a pain reduction effect, with approximately 70% of the patients reporting satisfaction with their treatment.^9^

References1

Brown
J
, 
Crawford
TJ
, 
Datta
S
, 
Prentice
A
, Cochrane Gynaecology and Fertility Group
. Oral contraceptives for pain associated with endometriosis. Cochrane Database Syst Rev. 2018;5:CD001019.2978682810.1002/14651858.CD001019.pub3PMC64946342

Vercellini
P
, 
Trespidi
L
, 
Colombo
A
, 
Vendola
N
, 
Marchini
M
, 
Crosignani
PG
. A gonadotropin‐releasing hormone agonist versus a low‐dose oral contraceptive for pelvic pain associated with endometriosis. Fertil Steril. 1993;60:75–9.85139623

Guzick
DS
, 
Huang
LS
, 
Broadman
BA
, 
Nealon
M
, 
Hornstein
MD
. Randomized trial of leuprolide versus continuous oral contraceptives in the treatment of endometriosis‐associated pelvic pain. Fertil Steril. 2011;95:1568–73.2130033910.1016/j.fertnstert.2011.01.027PMC42717944

Brown
J
, 
Kives
S
, 
Akhtar
M
. Progestagens and anti‐progestagens for pain associated with endometriosis. Cochrane Database Syst Rev. 2012;3:CD002122.10.1002/14651858.CD002122.pub2PMC6885053224192845

Harada
T
, 
Momoeda
M
, 
Taketani
Y
, 
Aso
T
, 
Fukunaga
M
, 
Hagino
H
, et al. Dienogest is as effective as intranasal buserelin acetate for the relief of pain symptoms associated with endometriosis‐a randomized, double‐blind, multicenter, controlled trial. Fertil Steril. 2009;91:675–81.1865318410.1016/j.fertnstert.2007.12.0806

Strowitzki
T
, 
Marr
J
, 
Gerlinger
C
, 
Faustmann
T
, 
Seitz
C
. Dienogest is as effective as leuprolide acetate in treating the painful symptoms of endometriosis; a 24 week, randomized, multicenter, open‐label trial. Hum Reprod. 2010;25:633–41.2008952210.1093/humrep/dep4697

Lan
S
, 
Ling
L
, 
Jianhong
Z
, 
Xijing
J
, 
Lihui
W
. Analysis of the levonorgestrel‐releasing intrauterine system in women with endometriosis. J Int Med Res. 2013;41:548–58.2366008710.1177/03000605134798658

Harada
T
, 
Kosaka
S
, 
Elliesen
J
, 
Yasuda
M
, 
Ito
M
, 
Momoeda
M
. Ethinylestradiol 20μg/drospirenone 3 mg in a flexible extended regimen for the management of endometriosis‐associated pelvic pain: a randomized controlled trial. Fertil Steril. 2017;108:798–805.2891192510.1016/j.fertnstert.2017.07.11659

Vercellini
P
, 
De Giorgi
O
, 
Mosconi
P
, 
Stellato
G
, 
Vicentini
S
, 
Crosignani
PG
. Cyproterone acetate versus a continuous monophasic oral contraceptive in the treatment of recurrent pelvic pain after conservative surgery for symptomatic endometriosis. Fertil Steril. 2002;77:52–61.1177959110.1016/s0015-0282(01)02951-x

## CQ 21 Is medical therapy effective for pain associated with deep endometriosis?

Norethisterone, LNG‐IUS, dienogest, and GnRH agonists are all effective for reducing pain.

Evidence level II

Strength of recommendation B

Numbers of studies referenced

Meta‐analyses, Systematic Reviews, Practice Guidelines 2.

RCTs, clinical trials11

Other epidemiologic studies 39

Commentary

Although there are several studies on medical therapy for pain associated with endometriosis, there are no systematic reviews on deep endometriosis. Six RCTs have examined medical therapy for deep endometriosis. Of these, two RCTs compared different medical therapies, two compared medical therapy with surgery, and two examined postoperative maintenance therapy.

1. RCTs comparing medical therapies

The two RCTs that compared medical therapies without surgery for deep endometriosis included agents not covered by medical insurance in Japan. In an RCT, which compared an implant releasing etonogestrel (synthetic progesterone)‐releasing implant (ENG implant) with LNG‐IUS (Mirena®),^1^ deep endometriosis was present in 45% of the patients in both groups. Pain and HR‐QOL improved in both groups, with no difference between groups. The other RCT treated pain associated with deep rectovaginal endometriosis with the aromatase inhibitor letrozole (2.5 mg/day) and then randomized the patients to also receive norethisterone (2.5 mg/day) or the GnRH agonist triptorelin (11.25 mg every 3 months) for 6 months.^2^ The norethisterone group reported significantly high satisfaction, while the triptorelin group demonstrated significantly greater reduction in endometriotic nodule volume. Nonmenstrual pain and dyspareunia decreased in both groups, with no differences between the groups. Discontinuation due to adverse effects was significantly more common in the triptorelin group. Bone density decreased significantly in the triptorelin group. This suggests that considering medical insurance coverage in Japan, LNG‐IUS and norethisterone are effective forms of medical therapy and can be used relatively safely.

2. RCTs comparing medical therapy and surgery

A RCT has compared surgical therapy with oral norethisterone for recurrent deep endometriosis.^3^ Patients who received low‐dose norethisterone (2.5 mg/day) and patients who underwent laparoscopic surgery demonstrated equivalent effects. The surgery group demonstrated early reduction of dyspareunia but partial recurrence of pain, while the analgesic effect of progestin was more gradual. The frequency of sexual intercourse was significantly higher in the norethisterone group than in the surgery group. Another RCT that included comparisons between surgery and medical therapy included comparisons between hormone therapy, surgery, and combined treatment, with pain as an outcome measure for patients with endometriosis, including deep endometriosis.^4^ Hormone therapy included progesterone, danazol, and GnRH agonists. Pregnancy rates did not differ significantly among the three groups. At 1 year, dysmenorrhea, dyspareunia, and abdominal pain recurred significantly less frequently in the combined treatment group than in the hormone therapy group or the surgery group. However, this study did not include patients with rectal or bladder endometriosis who had undergone rectal or bladder resection. Although the above results suggest that medical therapy with norethisterone is as effective as surgery for deep endometriosis, combined postoperative maintenance therapy and surgery may be the most effective treatment.

3. RCTs comparing postoperative maintenance therapies

Two RCTs related to agents used in postoperative maintenance therapy have examined GnRH agonists, dienogest, and OC/LEP. In a 2015 controlled trial, among patients who underwent complete laparoscopic excision, patients who underwent GnRH agonist treatment did not show any difference from those who did not undergo GnRH agonist treatment, while patients who underwent incomplete surgery without subsequent treatment demonstrated severe pain compared to patients who underwent complete excision and patients who underwent incomplete excision but subsequently received GnRH agonists.^5^ At 1 year, the complete excision group demonstrated less severe pain and better QOL than the incomplete excision group regardless of GnRH agonist treatment. These results lead to the conclusion that complete surgical excision is the most effective option to treat pain in deep endometriosis, while postoperative GnRH agonist treatment leads to temporary improvement in pain in patients who have undergone incomplete surgical treatment.

In an RCT, which divided patients into a dienogest group, goserelin group (GnRH agonist), and non‐treatment group to compare their efficacy in preventing endometriosis recurrence after laparoscopic surgery, the goserelin group and non‐treatment group did not differ significantly in terms of recurrence rate, while the recurrence rate differed significantly between the dienogest group and non‐treatment group.^6^ The dienogest group and the goserelin group demonstrated significant reductions in menstrual pain and chronic pelvic pain. Adverse effects were markedly more frequent in the goserelin group than in the dienogest group. This suggests that dienogest is effective in preventing postoperative recurrence of deep endometriosis and managing associated pain, while GnRH agonists offer promise for the temporary improvement of pain after incomplete excision of deep endometriosis.

References1

Carvalho
N
, 
Margatho
D
, 
Cursino
K
, 
Benetti‐Pinto
CL
, 
Bahamondes
L
. Control of endometriosis‐associated pain with etonogestrel‐releasing contraceptive implant and 52‐mg levonorgestrel‐releasing intrauterine system: randomized clinical trial. Fertil Steril. 2018;110:1129–36.3039655710.1016/j.fertnstert.2018.07.0032

Ferrero
S
, 
Venturini
PL
, 
Gillott
DJ
, 
Remorgida
V
. Letrozole and norethisterone acetate versus letrozole and triptorelin in the treatment of endometriosis related pain symptoms: a randomized controlled trial. Reprod Biol Endocrinol. 2011;9:88.2169303710.1186/1477-7827-9-88PMC31416453

Vercellini
P
, 
Somigliana
E
, 
Consonni
D
, 
Frattaruolo
MP
, 
de Giorgi
O
, 
Fedele
L
. Surgical versus medical treatment for endometriosis‐associated severe deep dyspareunia: I. effect on pain during intercourse and patient satisfaction. Hum Reprod. 2012;27:3450–9.2292684110.1093/humrep/des3134

Mettler
L
, 
Ruprai
R
, 
Alkatout
I
. Impact of medical and surgical treatment of endometriosis on the cure of endometriosis and pain. Biomed Res Int. 2014;2014:264653–9.2558042810.1155/2014/264653PMC42792625

Angioni
S
, 
Pontis
A
, 
Dessole
M
, 
Surico
D
, 
de Cicco Nardone
C
, 
Melis
I
. Pain control and quality of life after laparoscopic en‐block resection of deep infiltrating endometriosis (DIE) vs. incomplete surgical treatment with or without GnRHa administration after surgery. Arch Gynecol Obstet. 2015;291:363–70.2515102710.1007/s00404-014-3411-56

Takaesu
Y
, 
Nishi
H
, 
Kojima
J
, 
Sasaki
T
, 
Nagamitsu
Y
, 
Kato
R
, et al. Dienogest compared with gonadotropin‐releasing hormone agonist after conservative surgery for endometriosis. J Obstet Gynaecol Res. 2016;42:1152–8.2722533610.1111/jog.13023

## CQ 22 Are complementary and alternative therapies effective for endometriosis‐associated pain?

There are no relevant studies with a high level of evidence, and there are no reliably effective complementary or alternative therapies.

Evidence levelII

Strength of recommendation C

Numbers of studies referenced.

Meta‐analyses, systematic reviews, practice guidelines 30.

RCTs, clinical trials 36

Other epidemiological studies 22

Commentary

There are two meta‐analyses of Chinese herbal medicine that have examined Xuefu Zhuyu and Wenjing‐tang. There is also a systematic review of the effects of the Xuefu Zhuyu decoction (XZD) and Hyeolbuchukeo‐tang.^1^ A group, which received a combination of XZD and western medication, such as NSAIDs, demonstrated a significant effective response compared to a group that received only western medication (RR 1.18, 95% CI 1.11–1.25). The combined treatment group also showed significantly lower VAS scores at 3 months. In a comparison between the XZD‐only group and the western medication‐only group, the XZD‐only group showed a significantly higher response rate (RR 1.26, 95% CI 1.06–1.49). In a systematic review and meta‐analysis of 1736 patients selected from 18 RCTs for Wenjing decoction, while Wenjing decoction was significantly better than NSAIDs to suppress pain, the low quality of the trial precludes conclusions about the efficacy of Wenjing decoction.^2^ A Cochrane review related to Chinese herbal medicine for endometriosis reviewed two RCTs of the effects of Nei Yi Wan in China.^3^ Nei Yi Wan did not differ from gestrinone (affinity for androgen receptors and progesterone receptors) in terms of analgesic effects or effects on pregnancy rates, but demonstrated a better analgesic effect than danazol.

Multiple studies, several of them in Iran, have been conducted on complementary therapy for dysmenorrhea with herbs and dietary supplements. We will summarize the results for 12 herbs [chamomile (scientific names: *Matricaria chamomilla*, *M. recutita*, *Chamomilla recutita*)^*1^, cinnamon (scientific names: *Cinnamomum zeylanicum*, *C. verum*), Damask rose^*2^ (scientific name: *Rosa damascena*), dill^*3^ (scientific name: *Anethum graveolens*), fennel^*4^ (scientific name: *Foeniculum vulgare*), fenugreek^*5^ (scientific name: *Trigonella foenum‐graecum*), ginger (scientific name: *Zingiber officinale*), guava (scientific name: *Psidium guajava*), rhubarb^*6^ (scientific name: *Rheum emodi*), uzara^*7^ (scientific name: *Xysmalobium undulatum*), valerian^*8^ (scientific name: *Valeriana officinalis*), and zataria^*9^ (scientific name: *Zataria multiflora*)] and for dietary supplements (fish oil, melatonin, vitamin B1, vitamin D, vitamin E, and zinc sulfate). Fennel, chamomile, and zataria have demonstrated equivalent efficacy to analgesics for dysmenorrhea.^4^ In a meta‐analysis of ginger for dysmenorrhea, ginger demonstrated an analgesic effect superior to that of a placebo and equivalent to that of mefenamic acid, an NSAID.^5^ A Cochrane review included 27 RCTs (3101 participants) on dietary supplements for dysmenorrhea.^6^ The effectiveness of vitamin E, dill, guava, and fennel did not differ from that of a placebo or no treatment. The efficacy of fenugreek, fish oil, fish oil + vitamin B1, ginger, valerian, vitamin B1, zataria, and zinc sulfate was very limited. Dill, fennel, guava, valerian, rhubarb, and Damask rose did not show significant efficacy compared to NSAIDs. Chamomile was more effective than NSAIDs; however, the evidence was very limited. Regarding comparisons of different supplements, ginger and zinc sulfate did not differ in efficacy. Vitamin B1 was more effective than fish oil. Melatonin did not differ in efficacy from a placebo for endometriosis‐associated dysmenorrhea. Based on the above, there may be multiple dietary supplements that are effective.

Complementary physical therapy includes acupuncture, acupressure, transcutaneous electrical nerve stimulation (TENS), exercise therapy, and yoga. In systematic reviews and meta‐analyses for pain and dysmenorrhea associated with endometriosis, acupuncture and electroacupuncture were significantly more effective in reducing pain in comparison with a placebo or no treatment, while manual acupuncture and warm acupuncture were more effective for dysmenorrhea than NSAIDs and Chinese herbal medicine.^7^, ^8^, ^9^, ^10^ In a Cochrane review, although the evidence of low quality, auricular acupuncture was more effective than Chinese medicine, while Chinese herbal medicine and danazol did not differ significantly in efficacy.^11^ In another study, acupuncture reduced dysmenorrhea, while auricular acupuncture was three times as effective as Chinese herbal medicine and demonstrated a significant analgesic effect in severe dysmenorrhea.^12^ Furthermore, acupressure produced a stronger analgesic effect than a placebo.^10^ When acupuncture is used, the use of analgesics and perceived stress decrease, while QOL increases significantly.^13^ The above results demonstrate that acupuncture, acupressure, and electroacupuncture may be as effective as or more effective than analgesics.

TENS is a non‐invasive technique that is said to produce an analgesic effect by releasing endogenous opioids. In a Cochrane review, high‐frequency TENS was more effective for pain relief compared to a placebo.^14^ Regarding the guidelines of various academic societies, the Society of Obstetricians and Gynecologists of Canada (SOGC) gives a recommendation grade II‐B for high‐frequency TENS for dysmenorrhea in women for whom conventional treatment is ineffective^15^; the World Endometriosis Society (WES) reached a consensus grade of γ (majority) for the short‐term analgesic effect of TENS for dysmenorrhea^16^; and the American College of Obstetricians and Gynecologists (ACOG) states that although TENS is effective, there is not enough evidence to recommend it as a first‐line therapy of complementary/alternative therapies.^17^ Although the efficacy of exercise therapy and yoga is inconclusive, they tend to improve symptoms, while endometriosis tends to be less common among women who exercise.^7^ Heat therapy for the lower abdomen is commercially available. The WES guidelines give topical heat a consensus grade of γ and state that there is no evidence,^16^ while the SOGC gives heat therapy a recommendation of I‐A.^15^ The ACOG states that while evidence for heat therapy is limited, the data are promising, and recommends it due to its low cost and low risk of harm.^17^

A systematic review of 28 studies reported on the effects of aromatherapy on dysmenorrhea.^18^ In comparisons of a no treatment group and a placebo group with an aromatherapy group, the aromatherapy group demonstrated a significant improvement in dysmenorrhea compared to the other two groups. In a Cochrane review of nonsurgical interventions for chronic pelvic pain,^19^ women who underwent counseling after ultrasound demonstrated an improvement in pain compared to women who were treated with a standard “wait and see” policy (OR 6.77, 95% CI 2.83–16.19). Women who underwent writing therapy as a form of psychotherapy reported an improvement in pain more frequently than women who did not undergo writing therapy (OR 4.47, 95% CI 1.41–14.13). No other psychotherapies demonstrated a significant difference with a placebo or standard therapy. As a complementary therapy, the relaxation of the pelvic structures by pushing the pelvic floor muscles and the sacrococcygeal joint from the back after compression of the sacral nodules and longitudinal ligaments was more effective than counseling. However, to reduce variation, pelvic relaxation was performed for a single subject in this study, indicating that its technical difficulty is a concern. Magnetic therapy with a magnetic therapy device was ineffective for pain compared to placebo. As the above results show, counseling through writing therapy and pelvic relaxation may be effective for chronic pelvic pain.

In summary, Chinese herbal medicine (Hyeolbuchukeo‐tang and Wenjing‐tang), acupuncture, acupressure, and electroacupuncture can be as effective as or more effective than analgesics for pain associated with endometriosis. Herbs/supplements (fennel, chamomile, zataria, ginger, fenugreek, fish oil, valerian, vitamin B1, and zinc sulfate), exercise therapy, TENS, heat therapy, yoga, aromatherapy, psychotherapy with writing therapy, counseling + ultrasound scans, and pelvic relaxation can all be effective.

*1:Japanese name: Kamitsure

*2: The raw material for rose oil

*3:Umbellifer. Herbs, seasonings, herbal medicines. Chinese name: Jira

*4: Foeniculum. Used as seasoning. Chinese name: Uikyo

*5:Faboideae. An herb and spice originally from the Mediterranean region.

*6:Polygonaceae. Used as an herb in China, was also used as an herb in ancient Greece, and is considered edible. Japanese name: Shokuyou daiou

*7:An African plant of the family Apocynaceae. Also known as milkwort

*8:Caprifoliaceae. From Europe. Long used as an herb, effective for conditions such as insomnia and mania. Approved in Germany for insomnia and dysphoria.

*9:A plant of the family Lamiaceae. A herb used in Persia

References1

Leem
J
, 
Jo
J
, 
Kwon
CY
, 
Lee
H
, 
Park
KS
, 
Lee
JM
. Herbal medicine (Hyeolbuchukeo‐tang or Xuefu Zhuyu decoction) for treating primary dysmenorrhea: a systematic review and meta‐analysis of randomized controlled trials. Medicine (Baltimore). 2019;98:e14170.3070256910.1097/MD.0000000000014170PMC63808292

Gao
L
, 
Jia
C
, 
Zhang
H
, 
Ma
C
. Wenjing decoction (herbal medicine) for the treatment of primary dysmenorrhea: a systematic review and meta‐analysis. Arch Gynecol Obstet. 2017;296:679–89.2879147110.1007/s00404-017-4485-73

Flower
A
, 
Liu
JP
, 
Lewith
G
, 
Little
P
, 
Li
Q
, Cochrane Gynaecology and Fertility Group
. Chinese herbal medicine for endometriosis. Cochrane Database Syst Rev. 2012;5:CD006568.10.1002/14651858.CD006568.pub3PMC12817023225927124

Sharghi
M
, 
Mansurkhani
SM
, 
Larky
DA
, 
Kooti
W
, 
Niksefat
M
, 
Firoozbakht
M
, et al. An update and systematic review on the treatment of primary dysmenorrhea. JBRA Assist Reprod. 2019;23:51–7.3052115510.5935/1518-0557.20180083PMC63642815

Chen
CX
, 
Barrett
B
, 
Kwekkeboom
KL
. Efficacy of oral ginger (Zingiber officinale) for dysmenorrhea: a systematic review and meta‐analysis. Evid Based Complement Alternat Med. 2016;2016:6295737–10.2727475310.1155/2016/6295737PMC48719566

Pattanittum
P
, 
Kunyanone
N
, 
Brown
J
, 
Sangkomkamhang
US
, 
Barnes
J
, 
Seyfoddin
V
, et al. Dietary supplements for dysmenorrhoea. Cochrane Database Syst Rev. 2016;3:CD002124.2700031110.1002/14651858.CD002124.pub2PMC73871047

Mira
TAA
, 
Buen
MM
, 
Borges
MG
, 
Yela
DA
, 
Benetti‐Pinto
CL
. Systematic review and meta‐analysis of complementary treatments for women with symptomatic endometriosis. Int J Gynaecol Obstet. 2018;143:2–9.10.1002/ijgo.12576299447298

Woo
HL
, 
Ji
HR
, 
Pak
YK
, 
Lee
H
, 
Heo
SJ
, 
Lee
JM
, et al. The efficacy and safety of acupuncture in women with primary dysmenorrhea: a systematic review and meta‐analysis. Medicine (Baltimore). 2018;97:e11007.2987906110.1097/MD.0000000000011007PMC59994659

Yu
SY
, 
Lv
ZT
, 
Zhang
Q
, 
Yang
S
, 
Wu
X
, 
Hu
Y‐P
, et al. Electroacupuncture is beneficial for primary dysmenorrhea: the evidence from meta‐analysis of randomized controlled trials. Evid Based Complement Alternat Med. 2017;2017:1791258.2935896010.1155/2017/1791258PMC573563710

Smith
CA
, 
Zhu
X
, 
He
L
, 
Song
J
. Acupuncture for primary dysmenorrhoea. Cochrane Database Syst Rev. 2011;1:CD007854.10.1002/14651858.CD007854.pub22124969711

Brown
J
, 
Farquhar
C
. Endometriosis: an overview of cochrane reviews. Cochrane Database Syst Rev. 2014;3:CD009590.10.1002/14651858.CD009590.pub2PMC69844152461005012

Zhu
X
, 
Hamilton
KD
, 
McNicol
ED
. Acupuncture for pain in endometriosis. Cochrane Database Syst Rev. 2011;9:CD007864.10.1002/14651858.CD007864.pub2PMC100105962190171313

Lund
I
, 
Lundeberg
T
. Is acupuncture effective in the treatment of pain in endometriosis?
J Pain Res. 2016;9:157–65.2706937110.2147/JPR.S55580PMC481804414

Proctor
ML
, 
Smith
CA
, 
Farquhar
CM
, 
Stones
RW
. Transcutaneous electrical nerve stimulation and acupuncture for primary dysmenorrhoea. Cochrane Database Syst Rev. 2002;1:CD002123.10.1002/14651858.CD002123PMC80785211186962415

Burnett
M
, 
Lemyre
M
. No. 345‐primary dysmenorrhea consensus guideline. J Obstet Gynaecol Can. 2017;39:585–95.2862528610.1016/j.jogc.2016.12.02316

Johnson
NP
, 
Hummelshoj
L
, World Endometriosis Society Montpellier Consortium
. Consensus on current management of endometriosis. Hum Reprod. 2013;28:1552–68.2352891610.1093/humrep/det05017
ACOG Committee
. Opinion no. 760: dysmenorrhea and endometriosis in the adolescent. Obstet Gynecol. 2018;132:e249–58.3046169410.1097/AOG.000000000000297818

Song
JA
, 
Lee
MK
, 
Min
E
, 
Kim
ME
, 
Fike
G
, 
Hur
MH
. Effects of aromatherapy on dysmenorrhea: a systematic review and meta‐analysis. Int J Nurs Stud. 2018;84:1–11.2972955610.1016/j.ijnurstu.2018.01.01619

Cheong
YC
, 
Smotra
G
, 
Williams
AC
. Non‐surgical interventions for the management of chronic pelvic pain. Cochrane Database Syst Rev. 2014;3:CD008797.10.1002/14651858.CD008797.pub2PMC1098179124595586

## CQ 23 Are pre‐operative or post‐operative medical therapies effective at surgery for endometriosis‐associated pain?

1.There is not enough evidence to determine whether preoperative medical therapy is effective in improving the effects of surgical therapy.

Evidence level II

Strength of recommendation C

2. Performing medical therapy for as long as possible after surgery is effective in preventing recurrence of pain; however, short‐term medical therapy has not been shown to be effective.

Evidence level I

Strength of recommendation A

Numbers of studies referenced

Meta‐analysis 2

Systematic Reviews 4

RCTs 2

Practice Guidelines 1

Commentary

Although surgical therapy is effective for reducing pain symptoms associated with endometriosis, recurrence after surgical therapy (subjective symptoms; or objective findings in laparoscopy, ultrasound, etc.) is common. Hormone therapy for endometriosis directly or indirectly reduces endometriosis activity, thus alleviating symptoms. When function‐sparing surgery is chosen as surgical therapy, the question of whether the addition of preoperative and postoperative medical therapy helps to improve postoperative pain symptoms or inhibit recurrence is notable.

The 2014 ESHRE guideline examines postoperative medical therapy in terms of short‐term therapy (6 months postoperatively) versus long‐term therapy (≥6 months). This distinction is made according to the questions of whether short‐term therapy enhances the efficacy of surgery and whether long‐term therapy is effective in inhibiting the recurrence of symptoms.^1^ A Cochrane review on preoperative and postoperative medical therapy for endometriosis surgery cited in the ESHRE guideline primarily examined the effects of short‐term therapy on surgical treatment. Most of the RCTs identified in the review are related to the short‐term use of GnRH agonists.^2^ Two of these RCTs compared the use versus non‐use of preoperative medical therapy with GnRH agonists; however, these comparisons involved preoperative medical therapy versus surgery alone, leading to the conclusion that there is not enough evidence to determine whether preoperative medical therapy improved the efficacy of surgery. Additionally, while short‐term postoperative medical therapy tended to improve pelvic pain, dysmenorrhea, and dyspareunia at 12 months, a meta‐analysis of three RCTs did not show a significant association between symptoms recurrence and the use or non‐use of medical therapy. This review concluded that there was insufficient evidence to determine whether postoperative medical therapy reduces pain symptoms or inhibits recurrence.^2^ In contrast, in an RCT, which used postoperative desogestrel for 40 patients with endometriosis‐associated pain, the desogestrel group demonstrated a significant reduction in pain and low VAS scores at 6 months postoperatively.^3^

However, a review of four RCTs and three cohort studies that examined patients with ≥6 months of postoperative medical therapy found that while OCs/LEPs significantly suppressed the recurrence of dysmenorrhea, there was little evidence regarding their efficacy for inhibiting the recurrence of chronic pelvic pain and dyspareunia.^4^ In a meta‐analysis that identified and examined three RCTs and one prospective cohort study regarding how differences in oral OCs/LEPs following surgery for ovarian endometriotic cysts affected cyst recurrence, the postoperative recurrence of dysmenorrhea was less frequent with a continuous OC/LEP schedule than with a cyclic schedule. Furthermore, although the differences were not statistically significant, ovarian endometriotic cyst recurrence, non‐cyclic (chronic) pelvic pain, and dyspareunia recurrence also tended to be less common with a continuous schedule.^5^

LNG‐IUS (Mirena®) has been evaluated in terms of the efficacy of postoperative administration of progestin. A Cochrane review identified three RCTs, which assessed the efficacy of postoperative LNG‐IUS insertion for no more than 1 year. Two of these RCTs compared LNG‐IUS insertion to no postoperative treatment and found that the LNG‐IUS group demonstrated a significant inhibition of the recurrence of pain symptoms, a significant improvement in the duration of menstruation, and a trend toward more patients who were satisfied with their treatment. The other RCT, which compared LNG‐IUS with GnRH agonists, found that the two treatments were roughly equally effective, with no significant differences between them. Although the evidence was limited, the authors of this review concluded that postoperative LNG‐IUS insertion is effective for reducing recurrence of pain symptoms in endometriosis.^6^ Examinations of long‐term maintenance therapy with LNG‐IUS are as follows. In an RCT, which compared symptoms 30 months after cystectomy based on the use versus non‐use of LNG‐IUS after 6 months of postoperative GnRH agonist therapy, although endometriotic cyst recurrence did not differ significantly between groups, LNG‐IUS insertion was associated with significant reductions in dysmenorrhea recurrence, pain symptoms, and chronic pelvic pain.^7^ In a meta‐analysis, which compared LNG‐IUS with other forms of medical therapy (four RCTs, one prospective cohort study, and two retrospective studies), the pain reduction effect of LNG‐IUS was similar to that of GnRH agonists, the recurrence inhibition effect was similar to that of an OC/LEP and danazol, patient satisfaction was higher than with OC/LEP, and vaginal bleeding was significantly more frequent than with GnRH agonists.^8^

A recent review of 28 studies on the effects of various combined hormonal contraceptives and progestin‐only contraceptives for endometriosis did not demonstrate any evident superiority among any of the agents compared.^9^ No RCT has examined whether the long‐term postoperative administration of dienogest, which has been used frequently in Japan, helps reduce symptoms or recurrence.

References1

Dunselman
GA
, 
Vermeulen
N
, 
Becker
C
, 
Calhaz‐Jorge
C
, 
D'Hooghe
T
, 
De Bie
B
, et al. ESHRE guideline: management of women with endometriosis. Hum Reprod. 2014;29:400–12.2443577810.1093/humrep/det4572

Yap
C
, 
Furness
S
, 
Farquhar
C
. Pre and post operative medical therapy for endometriosis surgery. Cochrane Database Syst Rev. 2004;3:CD003678.10.1002/14651858.CD003678.pub2PMC6984629152664963

Tanmahasamut
P
, 
Saejong
R
, 
Rattanachaiyanont
M
, 
Angsuwathana
S
, 
Techatraisak
K
, 
Sanga‐Areekul
N
. Postoperative desogestrel for pelvic endometriosis‐related pain: a randomized controlled trial. Gynecol Endocrinol. 2017;33:534–9.2826623410.1080/09513590.2017.12961244

Koga
K
, 
Takamura
M
, 
Fujii
T
, 
Osuga
Y
. Prevention of the recurrence of symptom and lesions after conservative surgery for endometriosis. Fertil Steril. 2015;104:793–801.2635409310.1016/j.fertnstert.2015.08.0265

Muzii
L
, 
Di Tucci
C
, 
Achilli
C
, 
Di Donato
V
, 
Musella
A
, 
Palaia
I
, et al. Continuous versus cyclic oral contraceptives after laparoscopic excision of ovarian endometriomas: a systematic review and metaanalysis. Am J Obstet Gynecol. 2016;214:203–11.2636483210.1016/j.ajog.2015.08.0746

Abou‐Setta
AM
, 
Houston
B
, 
Al‐Inany
HG
, 
Farquhar
C
. Levonorgestrel‐releasing intrauterine device (LNG‐IUD) for symptomatic endometriosis following surgery. Cochrane Database Syst Rev. 2013;1:CD005072.10.1002/14651858.CD005072.pub3234407987

Chen
YJ
, 
Hsu
TF
, 
Huang
BS
, 
Tsai
HW
, 
Chang
YH
, 
Wang
PH
. Postoperative maintenance levonorgestrel‐releasing intrauterine system and endometrioma recurrence: a randomized controlled study. Am J Obstet Gynecol. 2017;216:582.e1–9.10.1016/j.ajog.2017.02.008282094888

Song
SY
, 
Park
M
, 
Lee
GW
, 
Lee
KH
, 
Chang
HK
, 
Kwak
SM
, et al. Efficacy of levonorgestrel releasing intrauterine system as a postoperative maintenance therapy of endometriosis: a meta‐analysis. Eur J Obstet Gynecol Reprod Biol. 2018;231:85–92.3033630910.1016/j.ejogrb.2018.10.0149

Grandi
G
, 
Barra
F
, 
Ferrero
S
, 
Sileo
FG
, 
Bertucci
E
, 
Napolitano
A
, et al. Hormonal contraception in women with endometriosis: a systematic review. Eur J Contracept Reprod Health Care. 2019;24:61–70.3066438310.1080/13625187.2018.1550576

## CQ 24 Is medical therapy following conservative surgery effective in preventing the recurrence of ovarian endometriotic cysts?

1. The long‐term administration of OC/LEP combinations is effective.

Long‐term administration of OCs/LEPs is effective.

Evidence level II

Strength of recommendation B

2. The intrauterine system releasing levonorgestrel (LNG‐IUS) does not evidently reduce the recurrence of ovarian endometriotic cysts.

Evidence level II

Strength of recommendation B

3. Long‐term administration of dienogest may reduce the recurrence of ovarian endometriotic cysts.

Evidence level IV

Strength of recommendation C

Numbers of studies referenced

1. Systematic Reviews 1, RCTs 1, Cohort Studies 3

2. Systematic Reviews 1, RCTs 2, Cohort Studies 1

3. Cohort studies 2

Commentary.

Studies of medical therapy following conservative surgery for ovarian endometriotic cysts are as follows. A systematic review and meta‐analysis have shown that the long‐term administration of an OC/LEP (≥ 12 months) inhibits the recurrence of ovarian endometriotic cysts (long‐term OC/LEP group: *n* = 423, and no treatment group: *n* = 341; RR 0.12, 95% CI 0.05–0.29).^1^ However, this meta‐analysis included only one RCT and three cohort studies. With the cyclic administration of OCs/LEPs for 24 months following surgery, recurrence rates did not differ significantly according to the type of progestin included in the OC/LEP regimen (desogestrel, gestodene, and dienogest) (one RCT).^2^

In a systematic‐review and meta‐analysis that compared cyclic versus continuous administration of OCs/LEPs, although the recurrence of ovarian endometriotic cysts tended to be less frequent with continuous administration, a significant difference was not observed with the sample size to date (continuous: *n* = 156, cyclic: n = 187; RR 0.54, 95% CI 0.28–1.05).^3^ This meta‐analysis included two RCTs and one cohort study.

The reported effects of the LNG‐IUS (Mirena®) following conservative surgery for ovarian endometriotic cysts are as follows. In a meta‐analysis of two RCTs (LNG‐IUS group: *n* = 68, and expectant management group: *n* = 66), the LNG‐IUS did not demonstrate evident efficacy in terms of reducing recurrence of ovarian endometriotic cysts (RR 0.60, 95% CI 0.31–1.14).^4^ The durations of postoperative observation in these two RCTs were 12 and 30 months. Although LNG‐IUS has not demonstrated a significant effect in terms of reducing the recurrence of ovarian endometriotic cysts, it has shown significant efficacy in reducing the recurrence of endometriosis compared to no treatment, and significant efficacy in reducing menstrual pain.^4^

In cohort studies, postoperative dienogest (2 mg/day) significantly reduced the rate of ovarian endometrioma compared to expectant management.^5^, ^6^ In comparisons between a dienogest group and an expectant management group, the rate of recurrence 12 and 24 months after surgery in the expectant management group was 16.5% and 24.0%, respectively, whereas the dienogest group had no recurrences.^5^ Studies with higher levels of evidence, such as RCTs, are desirable to assess the efficacy of dienogest in reducing postoperative recurrence of ovarian endometriotic cysts.

References1

Vercellini
P
, 
DE Matteis
S
, 
Somigliana
E
, 
Buggio
L
, 
Frattaruolo
MP
, 
Fedele
L
. Long‐term adjuvant therapy for the prevention of postoperative endometrioma recurrence: a systematic review and meta‐analysis. Acta Obstet Gynecol Scand. 2013;92:8–16.2264629510.1111/j.1600-0412.2012.01470.x2

Cucinella
G
, 
Granese
R
, 
Calagna
G
, 
Svelato
A
, 
Saitta
S
, 
Tonni
G
, et al. Oral contraceptives in the prevention of endometrioma recurrence: does the different progestins used make a difference?
Arch Gynecol Obstet. 2013;288:821–7.2358001110.1007/s00404-013-2841-93

Muzii
L
, 
Di Tucci
C
, 
Achilli
C
, 
Di Donato
V
, 
Musella
A
, 
Palaia
I
, et al. Continuous versus cyclic oral contraceptives after laparoscopic excision of ovarian endometriomas: a systematic review and metaanalysis. Am J Obstet Gynecol. 2016;214:203–11.2636483210.1016/j.ajog.2015.08.0744

Song
SY
, 
Park
M
, 
Lee
GW
, 
Lee
KH
, 
Chang
HK
, 
Kwak
SM
, et al. Efficacy of levonorgestrel releasing intrauterine system as a postoperative maintenance therapy of endometriosis: a meta‐analysis. Eur J Obstet Gynecol Reprod Biol. 2018;231:85–92.3033630910.1016/j.ejogrb.2018.10.0145

Adachi
K
, 
Takahashi
K
, 
Nakamura
K
, 
Otake
A
, 
Sasamoto
N
, 
Miyoshi
Y
, et al. Postoperative administration of dienogest for suppressing recurrence of disease and relieving pain in subjects with ovarian endometriomas. Gynecol Endocrinol. 2016;32:646–9.2689094810.3109/09513590.2016.11475476

Takaesu
Y
, 
Nishi
H
, 
Kojima
J
, 
Sasaki
T
, 
Nagamitsu
Y
, 
Kato
R
, et al. Dienogest compared with gonadotropin‐releasing hormone agonist after conservative surgery for endometriosis. J Obstet Gynaecol Res. 2016;42:1152–8.2722533610.1111/jog.13023

## Funding Information

This work was supported by the Japan Society of Obstetrics and Gynecology.

## Disclosure

The authors declare no conflicts of interests for this article.
